# Exploration of Benzenesulfonamide-Bearing Imidazole Derivatives Activity in Triple-Negative Breast Cancer and Melanoma 2D and 3D Cell Cultures

**DOI:** 10.3390/ph14111158

**Published:** 2021-11-13

**Authors:** Benas Balandis, Vytautas Mickevičius, Vilma Petrikaitė

**Affiliations:** 1 Department of Organic Chemistry, Kaunas University of Technology, Radvilėnų pl. 19, LT-50254 Kaunas, Lithuania; benas.balandis@ktu.lt; 2 Laboratory of Drug Targets Histopathology, Institute of Cardiology, Lithuanian University of Health Sciences, Sukilėlių pr. 13, LT-50162 Kaunas, Lithuania; vilmapetrikaite@gmail.com; 3 Institute of Physiology and Pharmacology, Faculty of Medicine, Lithuanian University of Health Sciences, A. Mickevičiaus g. 9, LT-44307 Kaunas, Lithuania

**Keywords:** benzenesulfonamides, imidazoles, alkylated, anticancer activity, colony formation, tumor spheroids

## Abstract

Heterocyclic compounds are one of the main groups of organic compounds possessing wide range of applications in various areas of science and their derivatives are present in many bioactive structures. They display a wide variety of biological activities. Recently, more and more attention has been focused to such heterocyclic compounds as azoles. In this work, we have synthesized a series of new imidazole derivatives incorporating a benzenesulfonamide moiety in their structure, which then were evaluated for their cytotoxicity against human triple-negative breast cancer MDA-MB-231 and human malignant melanoma IGR39 cell lines by MTT assay. Benzenesulfonamide-bearing imidazole derivatives containing 4-chloro and 3,4-dichlorosubstituents in benzene ring, and 2-ethylthio and 3-ethyl groups in imidazole ring have been determined as the most active compounds. Half-maximal effective concentration (EC_50_) of the most cytotoxic compound was 27.8 ± 2.8 µM against IGR39 cell line and 20.5 ± 3.6 µM against MDA-MB-231 cell line. Compounds reduced cell colony formation of both cell lines and inhibited the growth and viability of IGR39 cell spheroids more efficiently compared to triple-negative breast cancer spheroids.

## 1. Introduction

**Figure 1:**
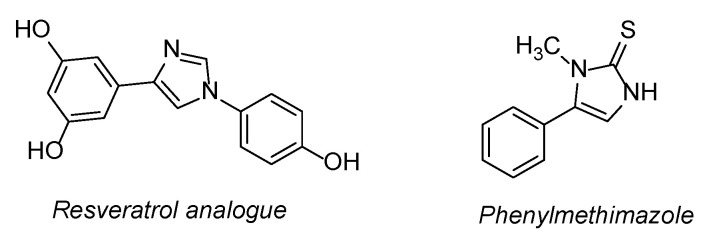
Imidazole molecules.

After huge breakthrough in pharmaceutical chemistry in early twentieth century, treating more and more previously incurable or difficult-to-treat diseases has become common practice in doctor’s offices nowadays. Research and synthesis of various functionalized heterocycle compounds have contributed significantly to this progress. Heterocycles are one of the largest classical divisions in organic chemistry and they play important role in modern drug design due to their wide range of biological properties [1]. A lot of marketed drugs contains aromatic or saturated, fused or spirocyclic heterocyclic moieties. The heteroatoms can provide potential binding interactions to the target and the cyclic nature limits structural flexibility, thus reducing the entropic penalty of binding [2]. Imidazole derivatives showed a wide scope of applicability in medicinal chemistry in recent years. Molecules bearing an imidazole moiety have become a promising antifungal [3], antibacterial [4,5], antituberculosis [6], and anti-inflammatory [7] agents. However, oncological diseases remain a major threat to the society due to their mutagenic and insidious nature [8,9]. Thus, there is an urgent need to develop new anticancer agents to combat these illnesses. Recently, imidazole derivatives gained a lot of interest in the development of lead molecules and drug candidates for cancer treatment [10,11,12]. For instance, newly designed trans-restricted analogues of resveratrol were synthesized in which the C–C double bond of the natural derivative has been replaced by 1,4-diaryl-substituted imidazole analogues [13]. One of the resveratrol analogues (Figure 1) showed very high cytotoxic activity and potency against NCI-60 tumor cells. Furthermore, a study was conducted, in which phenylmethimazole was tested against triple negative breast cancer cells MDA-MB-231, Hs578T, MDA-MB-468 (Figure 1) [14]. This research showed that phenylmethimazole significantly inhibited interleukin 6 (IL-6) expression by MDA-MB-231 and other triple-negative breast cancer (TNBC) cell lines.

Therefore, based on recent studies, benzenesulfonamide-bearing imidazole derivatives were synthesized in this work. Two cancer cell lines, namely TNBC cell line MDA-MB-231 and human melanoma cell line IGR39 were selected to test cytotoxic activity and compound effect on cell colony-forming ability. Both cancer types are considered very aggressive and have a low survival prognosis. Resistance to drugs occurs quite often in the case of melanoma treatment with targeted therapeutics, due to BRAF mutations [15]. More than half of patients with TNBC at an early stage have a recurrence of the disease, and about 40% of them die within the first 5 years [16]. We decided to explore synthesized compounds in clonogenic assay, which shows a single cell ability to grow into a colony [17] and mimics the cell ability to survive and proliferate after chemotherapy. We also tested novel compound effect on cancer cell spheroid (three-dimensional cultures) growth, as this model much better represents the real spatial connections between cells found in real tumor, compared to monolayers cell cultures [18,19]. As a continuation of our interest in further search for the nitrogen-containing heterocyclic molecules possessing biological activity [20,21,22], we report the synthesis of a series of compounds bearing benzenesulfonamide and imidazole moieties, and evaluation of their anticancer activity.

## 2. Results and Discussion

### 2.1. Chemistry

**Scheme 1:**
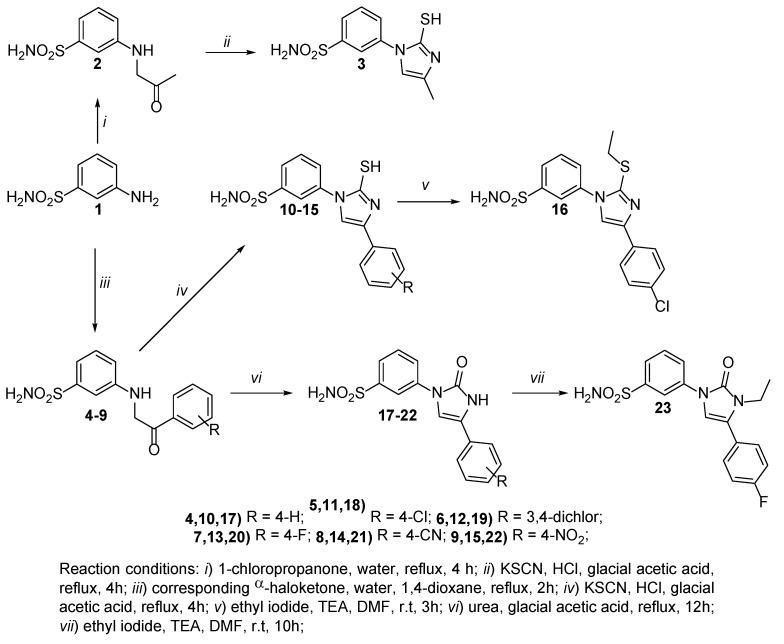
Synthesis of compounds **2**–**23**.

The 3-((2-oxopropyl)amino)benzenesulfonamide (**2**) was prepared in the reaction of amine **1** with 1-chloropropanone in small amount of water at reflux (Scheme 1). The structures of **2** and all other compounds have been confirmed by the data of FT-IR, ^1^H and ^13^C NMR spectroscopy as well as elemental analysis data. Later, compound **2** has been cyclized to imidazolethiol **3** during the reaction with KSCN in glacial acetic acid in a presence of HCl as a catalyst. In a ^1^H NMR spectrum of compound **3**, the singlets assigned to the protons in the CH group at 8.20 ppm and in the SH group at 12.39 ppm have proven the presence of 1*H*-imidazolethiol moiety in the molecule. It is widely known that similar thiolamides can exist in theirs thione/thiol forms due to ongoing tautomerism [23]. However, the carbon attributed to the C-SH group resonated at 161.29 ppm in the ^13^C NMR spectrum, which shows, that in this case, compound **3** exists as thiol in DMSO-d_6_ solution [24]. Moreover, in FT-IR spectrum, absorption at 2664 cm^−1^ was attributed to S-H bond stretching, which shows that thiol form is also predominant in the crystal. Furthermore, 3-aminobenzenesulfonamide (**1**) was treated with various α-haloketones in water/1,4-dioxane solution to obtain compounds **4**–**9**, which were later used as precursors for synthesis of corresponding imidazoles **10**–**15** in similar reaction conditions as were in product **3** synthesis (Scheme 1) [25]. We also found out, that these imidazole derivatives **10**–**15** (or the rest in this work) can be purified without using any organic solvents. For purification, these imidazole derivatives can be dissolved into 5% sodium hydroxide aqueous solution instead. Then, after filtration, the acidification of the obtained solution with glacial acetic acid will provide purer forms of the desired products without any significant loss to the purified amount of it. We also wanted to modify some of the pharmacokinetic properties of 1*H*-imidazole derivatives **10**–**15** to see, how it would affect the cancer cells. We decided to perform S-alkylation of 1*H*-imidazole **11** to reach higher solubility in organic solvents and lipophilicity. As a result, in the first attempt, imidazole **11** was treated with ethyl iodide in DMF overnight to obtain compound **16**. In a ^1^H NMR spectrum for **16**, the triplet assigned to the protons in the CH_3_ group at 1.30 ppm and quartet assigned to the protons in the CH_2_ group at 3.15 ppm have proved the presence of ethyl moiety in the molecule. Elemental analysis data of **16** confirmed that imidazole **16** did not form hydroiodide salt. Moreover, to increase reaction rate, we used triethylamine as a base catalyst, which shortened the reaction time to 3 h.

Furthermore, compounds **4**–**9** were dissolved in glacial acetic acid and treated with urea to afford oxoimidazoles **17**–**22** (Scheme 1). For instance, in a ^1^H NMR spectrum for **17**, the singlet was assigned to the proton of the NH group at 11.23 ppm. The resonances in ^13^C NMR spectrum at 106.27 ppm and 152.34 were attributed to CH or C=O groups respectively, which have proven the presence of oxoimidiazole moiety in the molecule. In addition, typical absorption of C=O bond stretching is also present in the FT-IR spectrum of compound **17** at 1703 cm^−1^. Similar results were also observed analyzing compounds **18**–**22** NMR spectra. Keto tautomer form was also justified after the reaction of imidazole **20** with ethyl iodide in DMF at room temperature. In the presence of triethylamine as base catalyst, after 10 h compound **23** was obtained. In a ^1^H NMR spectrum for **23**, the triplet at 1.30 ppm was assigned to the protons in the CH_3_ group and quartet at 3.14 ppm was assigned to the protons in the CH_2_ group to confirm that an alkylation of compound **20** was successful. However, in ^13^C NMR spectrum, the carbon attributed to the N-CH_2_ group resonated at 27.56 ppm. The chemical shift of this carbon atom was observed more towards the high field, than O-CH_2_ groups carbon would have resonated [26]. This confirms that N-alkylation occurred during the alkylation of imidazole **23**.

**Scheme 2:**
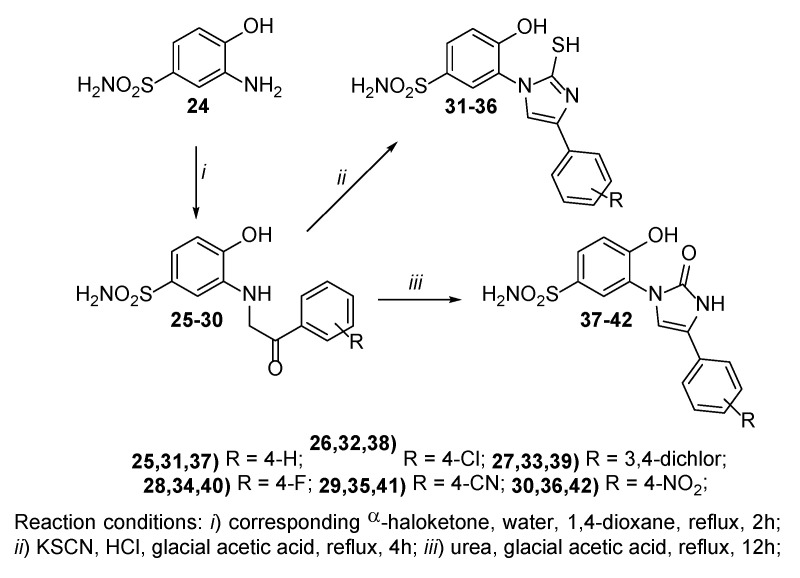
Synthesis of compounds **25**–**42**.

3-Amino-4-hydroxybenzenesulfonamide (**24**) was used in reactions with α-haloketones in water/1,4-dioxane mixture to obtain precursors **25**–**30** for further synthesis of imidazoles **31**–**36** (Scheme 2). Compounds **25**–**30** were treated with potassium thiocyanate and HCl in glacial acetic acid to afford 2-mercaptomidazoles **31**–**36**. For instance, in ^1^H NMR spectrum for **31**, the singlets at 10.89 ppm and 12.92 ppm were assigned to the protons in the OH at SH groups, respectively. In the ^13^C NMR spectrum for **31**, carbon attributed to the C-SH group resonated at 163.65 ppm. Furthermore, 2-oxo-1*H*-imidazoles **37**–**42** were obtained during the corresponding reactions between compounds **25**–**30** and urea in glacial acetic acid (Scheme 2). In a ^1^H NMR spectrum for **37**, the singlets were assigned to the protons in the OH group at 10.84 ppm and in the NH group at 11.11 ppm. Moreover, the resonances in ^13^C NMR spectrum at 109.71 ppm and 154.82 was attributed to CH and C=O groups respectively, which have proven the presence of 2-oxoimidiazole moiety in the molecule.

### 2.2. Anticancer Activity

**Figure 2:**
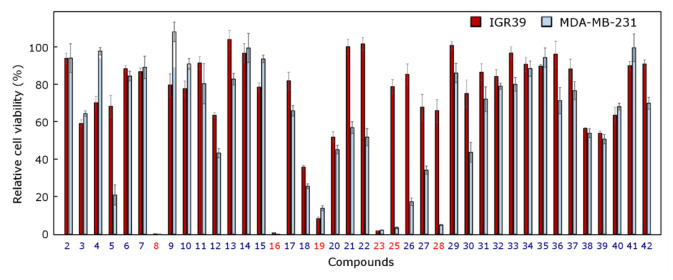
Effect of tested compounds on cancer cell viability at 100 µM concentration after 72 h of incubation, *n* = 3. Experiments were repeated three times, calculating the mean and standard deviation. The number of the most active compounds selected for further experiments are shown in red.

Tested imidazole derivatives showed different activity against human triple-negative breast cancer and human melanoma cell lines at 100 µM concentration (Figure 2). Almost half of compounds were more active against MDA-MB-231 cell line, and eight compounds showed higher cytotoxicity against IGR39 cell line. Other compounds possessed a similar activity against both types of cancer cells. More active compounds (**5**, **8**, **12**, **16**, **18**, **19**, **23**), that reduced cell viability below 50%, have been identified between non-hydroxylated compounds, especially with imidazolone fragment. 4-Hydroxybenzenesulfonamide derivatives with thioimidazole ring (compounds **31**–**36**) were practically inactive against both tested cell lines. It is worthy to notice, that the most active compounds in separate groups contained either 4-chloro (**5**, **18**, **26**, **38**) or 3,4-dichloro (**12**, **19**, **27**, **39**) substituents. The least active compounds were those containing cyano group in phenyl ring (the only active compound was **8**). Two compounds (**23** and **16**) contain 2-ethylthio and 3-ethyl substituents in imidazole ring, which could contribute to their high anticancer activity, and the importance of this fragment in this position could be of great interest in the future research.

**Figure 3:**
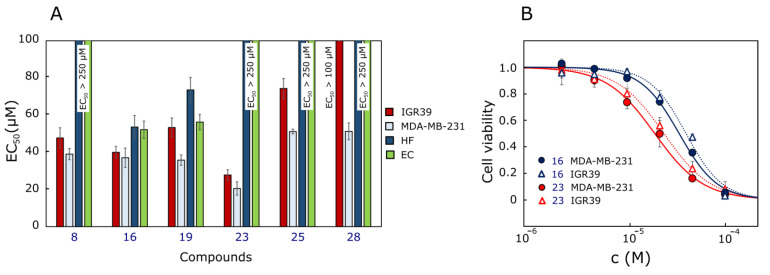
Effect of the most active compounds on cancer cell viability. (**A**). EC_50_ values of the most active compounds after 72 h of incubation by MTT assay. (**B**). Dose–response curves of compounds 16 and 23. *n* = 3. Experiments were repeated three times, calculating the mean and standard deviation.

The most active compounds were imidazole derivatives **8**, **16**, **19**, **23**, **25**, and **28**, which reduced cell viability of at least one cell line below 10%, and they were then tested more thoroughly both against human cancer cell lines and normal cells lines (fibroblasts (HF) and endothelial cells (EC)). The effective concentrations that reduce cell viability by 50% (EC_50_ values) have been established (Figure 3A).

Compound **23** was established to be the most active of tested compounds, its EC_50_ value was 27.8 ± 2.8 µM against IGR39 cell line and 20.5 ± 3.6 µM against MDA-MB-231 cell line. It was 1.4 times more active against IGR39 and 1.8 times more active against MDA-MB-231 cell lines, compared to the second most active compound **16** from this series (Figure 3B). Moreover, the selectivity of compound **23** against cancer cell lines compared to noncancerous cells was the highest between tested compounds. To compare with, IGR39 cell line was shown to be resistant to BRAF inhibitor dabrafenib, which is approved and used in clinics as a second line drug for malignant melanoma (EC_50_ value after 6 days of incubation was >50 µM) [27]. Dabrafenib reduces melanoma cell line SK-MEL-24 viability by MTT assay after 72 h of incubation at a concentration of 35.9 µM [28]. In general, malignant melanoma is characterized as highly mutated and lethal type of cancer [29], and for years was treated with conventional relatively toxic drugs with limited effectiveness [30]. However, despite the targeted therapy that affects BRAF and MEK pathways, the failure of treatment, side effects, and the development of resistance shows that there is a need of novel therapeutic strategies [31]. IGR39 cell line is considered to be as one the most resistant to drugs melanoma cell lines [32] and there are limited data of agent activity in this cell line. Another cell line A375, which is more sensitive to drugs, responds to the treatment with dacarbazine better, but still after 48 h of incubation with it, half of cells remained viable when incubated with 25–50 µg/mL of drug [33]. Meanwhile, targeted BRAF inhibitor vemurafenib achieves 50% of A375 cell viability reduction at 139 nM concentration but only at >10 µM concentration reduces human melanoma cell line SK-MEL-2 viability by 50% [34].

Doxorubicin, which is considered as a chemotherapeutic agent against triple-negative breast cancer, reduces MDA-MB-231 cell viability below 50% at 5 μM concentration [35], and strategies of combining this drug with the other compounds sensitizing cells to its treatment [36] or enhancing doxorubicin transport inside [37], are widely explored. This type of cancer has limited treatment option, and quite often includes such non-specific drugs as anthracyclines, taxanes, platinum derivatives, and fluorouracil [38]. As this type of cancer is very heterogenous and has limited possibilities for development of targeted agents, available more specific therapeutics show limited effectiveness. It is very invasive and resistant to many available drugs [16], thus search for the newer, more effective agents for this type of cancer is a long-term ongoing process.

Both types of cancer are characterized as recurrent, thus we decided to test the compound effect on single cell ability to survive and proliferate using clonogenic assay. The most active agents **8**, **16**, **19**, **23**, **25**, and **28** were tested for their effect on IGR39 and MDA-MB-231 cell colony formation at 50% of their established EC_50_ concentrations.

**Figure 4:**
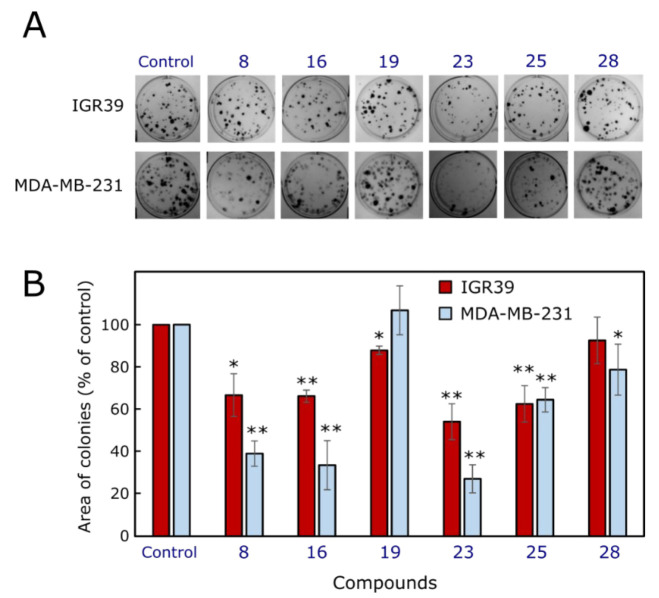
Effect of compounds on cell colony formation. (**A**) Photos of human melanoma IGR39 and human triple-negative breast cancer MDA-MB-231 colonies at the end of experiment. (**B**) Area of colonies at the end of experiment. Asterisks (*) indicate *p* < 0.05 and (**) indicate *p* < 0.01 compared to the control (untreated cells). Experiments were repeated three times, calculating the mean and standard deviation.

Almost all compounds showed greater activity on MDA-MB-231 cell colony growth (Figure 4). The most active compound **23**, which was the most cytotoxic in MTT assay, showed comparable activity with compounds **8** and **16** against both cell lines. In the presence of compound **23**, MDA-MB-231 cell colony area dropped down to 27.1 ± 6.6%, compared to the control (*p* < 0.05) (Figure 4). All three compounds produced almost twice more expressed effect on MDA-MB-231 than IGR39 cell line. Compounds **19** and **28** were active only on one of tested cell line.

As previously mentioned, the strategy of combinational therapy is being extensively studied and investigated whether this approach could reduce the resistance of cancer to available therapy. It has been established MERTK inhibitor UNC2025 reduces colony formation of BRAF mutant and BRAF wild-type cell lines at 300–500 nM concentration [39]. Combinations of dacarbazine and all-trans retinoic acid were shown to be more active against the B16F10 melanoma cell colony growth compared to dacarbazine alone [40]. Yuan et al. [28] determined that the combination of 3.2 µM A100 and 0.8 µM dabrafenib decreases cell growth of SK-MEL-24 more efficiently compared to single agent dabrafenib. However, this effect has not been found in the case of other tested melanoma cell lines A375 and WM115. It means, that the different response to the same drug and its combination exist between different cell lines, thus it would be worthy to study the effect of our synthesized derivatives in some other melanoma cell lines, too.

Many studies have been done on MDA-MB-231 cell colony formation in the presence of drugs used for triple-negative breast cancer treatment, novel compounds, or their combinations. Franco et al. [41] established that MDA-MB-231 cell colony formation is reduced more efficiently by 70 nM of doxorubicin compared to 70 nM of paclitaxel. Alkaloid aloperine derived from plants could inhibit MDA-MB-231 cell colony formation number up to ~40% compared to the control; however, at rather high concentration of 400 µM [42]. By using clonogenic assay, it has been shown that the clinically used cholesterol level lowering drug pitavastatin it 90% from its EC_50_ value could reduce MDA-MB-231 cell colony formation up to 40% [43]. Combination of 5 nM eribulin (non-taxane microtubule dynamics inhibitor) and CDK2 inhibitor 300 nM CYC065 reduced the MDA-MB-231 cell colony growth after 4 days treatment [44]. In summary, the beneficial effect of different compounds and their combinations on MDA-MB-231 cell colony formation ability provides hopes in combating the triple-negative breast cancer recurrence after the treatment. Novel benzenesulfonamide-bearing imidazole derivatives could be also explored as sensitizers of cells to the conventional therapy.

**Figure 5:**
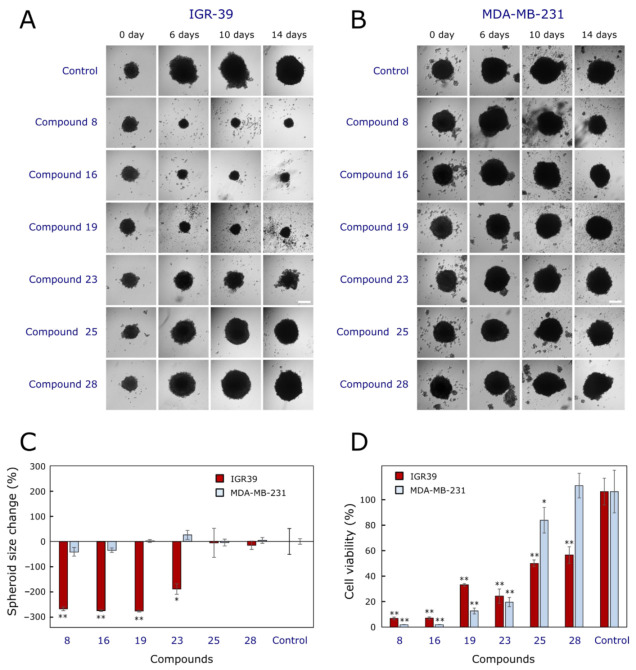
Effect of compounds on 3D cell cultures. (**A**) Photos of human melanoma IGR39 tumor spheroids at different periods of experiment. (**B**) Photos of human triple-negative breast cancer MDA-MB-231 tumor spheroids at different periods of experiment. (**C**) Spheroid size at the end of experiment. (**D**) Cell viability in MDA-MB-231 and IGR39 spheroids. Asterisks (*) indicate *p* < 0.05 and (**) indicate *p* < 0.01 compared to the control (untreated spheroids). Scale bars indicated 200 µm. Each group contained 8–10 spheroids. Data are presented as means ± standard deviations.

Three-dimensional cell models are gaining more and more attention from cell biologists, especially in testing activity and mechanisms of novel substances due to their high resemblance to the real tumors [45,46]. It has been shown that such cell systems regain tissue-specific functions and are more predictive as in vivo models compared to cell monolayers [47]. Thus, the effects of novel benzenesulfonamide-bearing imidazole derivatives **8**, **16**, **19**, **23**, **25**, and **28** on melanoma IGR39 and triple-negative breast cancer MDA-MB-231 cell spheroid growth (Figure 5) were studied. As shown in Figure 5C, all tested compounds reduced IGR39 spheroid growth much more efficiently in comparison to the control group. Meanwhile, the effect of compounds on MDA-MB-231 spheroid growth was negligible. Compounds **8**, **16**, and **19** showed a similar inhibitory effect on IGR39 spheroid growth, however the most cytotoxic compound **23** was not the most active one in 3D cultures. Compounds **25** and **28** did not show activity in both types of spheroids, compared to control groups (Figure 5C).

It is known that compounds’ effect on spheroid growth does not necessarily correlate with their effect on cell viability [48]. We determined that the only compound **28** did not reduce cell viability in MDA-MB-231 spheroids (Figure 5D). Meanwhile, all other compounds diminished cell viability in both types of spheroids, and compound **28** also reduced viability of IGR39 3D cultures. Interestingly, differences between cell viability in spheroids were not so big as the size of spheroids. In addition, despite the size of IGR39 spheroids treated with compound **19** was the smallest one between all groups, the viability of these spheroids was higher compared to those incubated with compounds **8**, **16**, and **23**. This could be explained that cells inside the spheroids could become more hypoxic, denser and this could lead to a lower viability. Some spheroids, especially treated with compound **8** and **19**, at the end of experiment began to disintegrate (Figure 5A,B). It has been shown that cytostatic drug docetaxel affects external cells in melanoma spheroids formed from three types of cells: melanoma cells, keratinocytes, and fibroblasts [49]. In our case, spheroids were also formed from several types of cells: cancer cells and fibroblasts. We could only speculate that the compounds could have different effects both on different cells and different zones in spheroids, but it has yet to be established yet.

Hundsberger et al. [50] demonstrated that a huge differences could be detected between 2D and 3D cultures depending on cell line and different concentrations of the same compound. Huang et al. [51] established that triple-negative breast cancer spheroids exhibit an increased resistance to anticancer compounds due significantly up-regulated levels of EMT-associated proteins. Similarly, in our research we established higher resistance of MDA-MB-231 spheroids to the tested compounds in comparison with melanoma cell line, and the differences between 2D and 3D models were shown.

In summary, benzenesulfonamide-bearing imidazole derivatives **8**, **16**, and **23** have shown melanoma and triple-negative breast cancer cell colony formation and tumor spheroid growth inhibition activity and require further attention as possible anticancer agents from very aggressive and invasive types of tumours.

## 3. Materials and Methods

### 3.1. Chemistry

Reagents were purchased from Sigma-Aldrich (St. Louis, MO, USA). The reaction course and purity of the synthesized compounds were monitored by TLC using aluminum plates pre-coated with silica gel 60 F_254_ (MerckKGaA, Darmstadt, Germany). Melting points were determined on a MEL-TEMP (Electrothermal, Bibby Scientific Company, Burlington, NJ, USA) melting point apparatus and are uncorrected. IR spectra (*ν*, cm^−1^) were recorded on a Perkin–Elmer Spectrum BX FT-IR spectrometer (PerkinElmer, Inc., Waltham, Massachusetts, United States) using KBr pellets. The ^1^H and ^13^C-NMR spectra were recorded in DMSO-d_6_ on a Bruker Avance III (400 MHz, 101 MHz) spectrometer. Chemical shifts (δ) are reported in parts per million (ppm) calibrated from TMS (0 ppm) as an internal standard for ^1^H NMR, and DMSO-d_6_ (39.43 ppm) for ^13^C NMR. Elemental analyses (C, H, N) were performed on an Elemental Analyzer CE-440 (Exeter Analytical, Inc., North Chelmsford, MA, USA). Compounds **2-23** and **25-42** synthesis methods, identification data and NMR spectra can be found in Supplementary Materials file. The peak shapes are denoted as follows: s, singlet; d, doublet; t, triplet; q, quartet; m, multiplet; br, broad.

### 3.2. Cell Culturing

The human melanoma IGR39 and the human triple-negative breast cancer MDA-MB-231 cell lines, as well as the human endothelial cells (EC) CRL-1730 were obtained from the American Type Culture Collection (ATCC, Manassas, VA, USA). Human foreskin fibroblasts (HF) CRL-4001 were originally obtained from ATCC and kindly provided by Prof. Helder Santos (University of Helsinki, Finland). IGR39, MDA-MB-231, EC, and HF were cultured in Dulbecco’s Modified Eagle’s GlutaMAX medium (Gibco (Carlsbad, CA, USA)). Medium was supplemented with 10,000 U/mL penicillin, 10 mg/mL streptomycin (Gibco), and 10% fetal bovine serum (Gibco). Cells were incubated at 37 °C in a humidified atmosphere containing 5% CO_2_. They were used until the passage of 20.

### 3.3. Cell Viability Assay

Cell viability was tested using 3-(4,5-dimethylthiazol-2-yl)-2,5-diphenyltetrazolium bromide (MTT; Sigma-Aldrich Co., St. Louis, MO, USA) assay, as described elsewhere [52]. Briefly, IGR39 and MDA-MB-231 cells were seeded in 96-well plates (Corning) in triplicate repeats at a volume of 100 μL (5 × 10^3^ cells/well). After 24 h, the cells were treated with 100 μM of various concentrations of tested compounds. Only medium without cells was used as a positive control, and the medium with 0.5% DMSO (Sigma-Aldrich Co.) served as a negative control. After 72 h, the medium from cells was discarded and the fresh medium containing 0.5 mg/mL of MTT solution (Sigma-Aldrich Co.) was added. Cell were incubated for the next 4 h. Then the liquid was aspirated, and the colored formazan product was dissolved in 100 μL DMSO (Sigma-Aldrich Co.). The absorbance was measured at 570 and 630 nm using a multi-detection microplate reader. Compound effect on cell viability was calculated using a formula:

$$Relative\;cell\;viability\;\left(\%\right)=\frac{A-A_{0}}{A_{NC}-A_{0}}\;$$

where:

*A*—mean of absorbance of tested compound,

*A*_0_—mean of absorbance of blank (no cells, positive control),

*A_NC_*—mean of absorbance of negative control (only cells, no treatment).

For establishing EC_50_ of the most active compounds, the same MTT procedure has been applied, only the compound serial dilutions from 50 µM to 1.56 µM have been made in a medium and added to the cells in triplicate repeats. EC_50_ value that represent the concentration of a compound causing 50% reduction of cancer cell metabolic activity has been calculated using Hill equation.

### 3.4. Colony Formation Assay

Colony formation assay was applied to evaluate the inhibitory effect of the most active compounds on cell ability to survive, and proliferate by forming colonies, as described elsewhere [43]. Briefly, both, melanoma IGR39 and triple-negative breast cancer MDA-MB-231 cells were seeded into 12-well plates in triplicate repeats (2 × 10^2^ cells/well) and grown in DMEM GlutaMAX supplemented with 10% FBS (ThermoFisher Sicentific, Waltham, MA, USA) and 1% antibiotics (ThermoFisher Scientific, Waltham, MA, USA) at 37 °C in a humidified atmosphere containing 5% CO_2_. After 24 h, the fresh medium containing tested compound 8, 16, 19, 23, 25, and 28 at a concentration representing 50% of calculated EC_50_ values were added to cells, followed by incubation at 37 °C in a humidified atmosphere containing 5% CO_2_ for the next 10 days (for IGR39 cell line) and 12 days (for MDA-MB-231 cell line), replenishing the media with the compounds once a week. Cells treated with medium containing 0.5% DMSO served as a negative control. After incubation, the colonies were stained with 0.1% Crystal Violet (Sigma-Aldrich Co.) solution. To start with, the media from cells was removed and the cells were washed once with sterile PBS. Then, the cells were fixed in a 4% formaldehyde (Thermo Scientific) solution and washed with PBS two times to remove the fixative, and stained with Crystal Violet solution for 20 min. After the stain had been removed, the remaining stain residues were washed three times sterile water. Lastly, the plates were dried overnight, and imaged with SYNGENE G:BOX gel doc system (Synoptics Limited, Cambridge, UK), using GeneSys software version 1.5.5.0, followed by quantification Gene tools software version 4.3.8.

### 3.5. Compound Activity in Spheroids

Magnetic 3D Bioprinting method was used to form spheroids, as described elsewhere [43]. Briefly, melanoma IGR39, triple-negative breast cancer MDA-MB-231 cells and human fibroblasts at 70% confluency in a 6-well plate were incubated with Nanoshuttle (Nano 3D Biosciences, Inc., Houston, TX, USA) for 8 h at 37 °C in a humidified atmosphere containing 5% CO_2_. Then, cells were trypsinized, centrifuged, and seeded into ultra-low attachment 96-well plate in a volume of 100 µL (1 × 10^3^ IGR39 and 1 × 10^3^ human fibroblasts/well, and 2 × 10^3^ MDA-MB-231 and 2 × 10^3^ human fibroblasts/well). The plate was placed on a magnetic drive (Nano 3D Biosciences, Inc., Houston, TX, USA) and incubated for 2 days at 37 °C in a humidified atmosphere containing 5% CO_2_. Then the fresh medium containing tested compound concentration representing 100% of calculated EC_50_ values was added to the wells. The spheroids were captured every two days using the Olympus IX73 inverted microscope (OLYMPUS CORPORATION). The quantitative analysis of compound anticancer activity in spheroids was performed using ImageJ (National Institutes of Health) and Microsoft Office Excel 2016 software.

### 3.6. Statistical Analysis

All biological experiments were repeated at least three times, calculating the mean and standard deviation. The data was processes using Microsoft Office Excel 2016 software (Microsoft Corporation, Redmond, WA, USA). Statistical analysis was performed by using Student’s t-test. The level of significance was set as *p* < 0.05.

## 4. Conclusions

In this study, the chemical transformations of benzenesulfonamides were carried out and a series of 1-substituted imidazoles with aliphatic, aromatic fragments were synthesized and evaluated for their anticancer activities in 2D and 3D models in vitro.

In the MTT assay, compounds were more effective against human triple-negative breast cancer MDA-MB-231 cell line, compared to malignant melanoma IGR39 cell line. Benzenesulfonamide-bearing imidazole derivatives containing 4-chloro and 3,4-dichlorosubstituents in benzene ring, and 2-ethylthio and 3-ethyl groups in imidazole ring have been determined as the most active compounds. These compounds reduced cell colony formation of both cell lines. Tested derivatives inhibited the growth and viability of IGR39 cell spheroids more efficiently compared to triple-negative breast cancer spheroids.

The obtained results suggest that benzenesulfonamide-bearing imidazole could be a suitable core for the anticancer compounds against such aggressive and invasive tumour types as malignant melanoma and triple-negative breast cancer.

## Data Availability

Data is contained within the article or Supplementary Materials.

## Supplementary Materials

The following are available online at https://www.mdpi.com/article/10.3390/ph14111158/s1, Figure S1: 1H NMR of compound **2** at 400 MHz (DMSO-*d_6_*), Figure S2: ^13^C NMR of compound **2** at 101 MHz (DMSO-*d_6_*), Figure S3: ^1^H NMR of compound **3** at 400 MHz (DMSO-*d_6_*), Figure S4: ^13^C NMR of compound **3** at 101 MHz (DMSO-*d_6_*), Figure S5: ^1^H NMR of compound **4** at 400 MHz (DMSO-*d_6_*), Figure S6: ^13^C NMR of compound **4** at 101 MHz (DMSO-*d_6_*), Figure S7: ^1^H NMR of compound **5** at 400 MHz (DMSO-*d_6_*), Figure S8: ^13^C NMR of compound **5** at 101 MHz (DMSO-*d_6_*), Figure S9: ^1^H NMR of compound **6** at 400 MHz (DMSO-*d_6_*), Figure S10: ^13^C NMR of compound **6** at 101 MHz (DMSO-*d_6_*), Figure S11: ^1^H NMR of compound **7** at 400 MHz (DMSO-*d_6_*), Figure S12: ^13^C NMR of compound **7** at 101 MHz (DMSO-*d_6_*), Figure S13: ^1^H NMR of compound **8** at 400 MHz (DMSO-*d_6_*), Figure S14: ^13^C NMR of compound **8** at 101 MHz (DMSO-*d_6_*), Figure S15: ^1^H NMR of compound **9** at 400 MHz (DMSO-*d_6_*), Figure S16: ^13^C NMR of compound **9** at 101 MHz (DMSO-*d_6_*), Figure S17: ^1^H NMR of compound **10** at 400 MHz (DMSO-*d_6_*), Figure S18: ^13^C NMR of compound **10** at 101 MHz (DMSO-*d_6_*), Figure S19: ^1^H NMR of compound **11** at 400 MHz (DMSO-*d_6_*), Figure S20: ^13^C NMR of compound **11** at 101 MHz (DMSO-*d_6_*), Figure S21: ^1^H NMR of compound **12** at 400 MHz (DMSO-*d_6_*), Figure S22: ^13^C NMR of compound **12** at 101 MHz (DMSO-*d_6_*), Figure S23: ^1^H NMR of compound **13** at 400 MHz (DMSO-*d_6_*), Figure S24: ^13^C NMR of compound **13** at 101 MHz (DMSO-*d_6_*), Figure S25: ^1^H NMR of compound **14** at 400 MHz (DMSO-*d_6_*), Figure S26: ^13^C NMR of compound **14** at 101 MHz (DMSO-*d_6_*), Figure S27: ^1^H NMR of compound **15** at 400 MHz (DMSO-*d_6_*), Figure S28: ^13^C NMR of compound **15** at 101 MHz (DMSO-*d_6_*), Figure S29: ^1^H NMR of compound **16** at 400 MHz (DMSO-*d_6_*), Figure S30: ^13^C NMR of compound **16** at 101 MHz (DMSO-*d_6_*), Figure S31: ^1^H NMR of compound **17** at 400 MHz (DMSO-*d_6_*), Figure S32: ^13^C NMR of compound **17** at 101 MHz (DMSO-*d_6_*), Figure S33: ^1^H NMR of compound **18** at 400 MHz (DMSO-*d_6_*), Figure S34: ^13^C NMR of compound **18** at 101 MHz (DMSO-*d_6_*), Figure S35: ^1^H NMR of compound **19** at 400 MHz (DMSO-*d_6_*), Figure S36: ^13^C NMR of compound **19** at 101 MHz (DMSO-*d_6_*), Figure S37: ^1^H NMR of compound **20** at 400 MHz (DMSO-*d_6_*), Figure S38: ^13^C NMR of compound **20** at 101 MHz (DMSO-*d_6_*), Figure S39: ^1^H NMR of compound **21** at 400 MHz (DMSO-*d_6_*), Figure S40: ^13^C NMR of compound **21** at 101 MHz (DMSO-*d_6_*), Figure S41: ^1^H NMR of compound **22** at 400 MHz (DMSO-*d_6_*), Figure S42: ^13^C NMR of compound **22** at 101 MHz (DMSO-*d_6_*), Figure S43: ^1^H NMR of compound **23** at 400 MHz (DMSO-*d_6_*), Figure S44: ^13^C NMR of compound **23** at 101 MHz (DMSO-*d_6_*), Figure S45: ^1^H NMR of compound **25** at 400 MHz (DMSO-*d_6_*), Figure S46: ^13^C NMR of compound **25** at 101 MHz (DMSO-*d_6_*), Figure S47: ^1^H NMR of compound **26** at 400 MHz (DMSO-*d_6_*), Figure S48: ^13^C NMR of compound **26** at 101 MHz (DMSO-*d_6_*), Figure S49: ^1^H NMR of compound **27** at 400 MHz (DMSO-*d_6_*), Figure S50: ^13^C NMR of compound **27** at 101 MHz (DMSO-*d_6_*), Figure S51: ^1^H NMR of compound **28** at 400 MHz (DMSO-*d_6_*), Figure S52: ^13^C NMR of compound **28** at 101 MHz (DMSO-*d_6_*), Figure S53: ^1^H NMR of compound **29** at 400 MHz (DMSO-*d_6_*), Figure S54: ^13^C NMR of compound **29** at 101 MHz (DMSO-*d_6_*), Figure S55: ^1^H NMR of compound **30** at 400 MHz (DMSO-*d_6_*), Figure S56: ^13^C NMR of compound **30** at 101 MHz (DMSO-*d_6_*), Figure S57: ^1^H NMR of compound **31** at 400 MHz (DMSO-*d_6_*), Figure S58: ^13^C NMR of compound **31** at 101 MHz (DMSO-*d_6_*), Figure S59: ^1^H NMR of compound **32** at 400 MHz (DMSO-*d_6_*), Figure S60: ^13^C NMR of compound **32** at 101 MHz (DMSO-*d_6_*), Figure S61: ^1^H NMR of compound **33** at 400 MHz (DMSO-*d_6_*), Figure S62: ^13^C NMR of compound **33** at 101 MHz (DMSO-*d_6_*), Figure S63: ^1^H NMR of compound **34** at 400 MHz (DMSO-*d_6_*), Figure S64: ^13^C NMR of compound **34** at 101 MHz (DMSO-*d_6_*), Figure S65: ^1^H NMR of compound **35** at 400 MHz (DMSO-*d_6_*), Figure S66: ^13^C NMR of compound **35** at 101 MHz (DMSO-*d_6_*), Figure S67: ^1^H NMR of compound **36** at 400 MHz (DMSO-*d_6_*), Figure S68: ^13^C NMR of compound **36** at 101 MHz (DMSO-*d_6_*), Figure S69: ^1^H NMR of compound **37** at 400 MHz (DMSO-*d_6_*), Figure S70: ^13^C NMR of compound **37** at 101 MHz (DMSO-*d_6_*), Figure S71: ^1^H NMR of compound **38** at 400 MHz (DMSO-*d_6_*), Figure S72: ^13^C NMR of compound **38** at 101 MHz (DMSO-*d_6_*), Figure S73: ^1^H NMR of compound **39** at 400 MHz (DMSO-*d_6_*), Figure S74: ^13^C NMR of compound **39** at 101 MHz (DMSO-*d_6_*), Figure S75: ^1^H NMR of compound **40** at 400 MHz (DMSO-*d_6_*), Figure S76: ^13^C NMR of compound **40** at 101 MHz (DMSO-*d_6_*), Figure S77: ^1^H NMR of compound **41** at 400 MHz (DMSO-*d_6_*), Figure S78: ^13^C NMR of compound **41** at 101 MHz (DMSO-*d_6_*), Figure S79: ^1^H NMR of compound **42** at 400 MHz (DMSO-*d_6_*), Figure S80: ^13^C NMR of compound **42** at 101 MHz (DMSO-*d_6_*).
