# Supplementary material for: Exploration of Benzenesulfonamide-Bearing Imidazole Derivatives Activity in Triple-Negative Breast Cancer and Melanoma 2D and 3D Cell Cultures
Source: Pharmaceuticals (Basel). 2021 Nov 13;14(11):1158. doi: 10.3390/ph14111158 (PMC8625351; doi:10.3390/ph14111158)
Supplement: Supplementary file 1 [file pharmaceuticals-14-01158-s001.zip › pharmaceuticals-1454421-supplementary.pdf]

Exploration of Benzenesulfonamide-Bearing Imidazole Derivatives Activity in Triple-Negative Breast Cancer and Melanoma 2D and 3D Cell Cultures

Benas Balandis [1], Vytautas Mickevičius [1], Vilma Petrikaitė [2,3]

[1]Department of Organic Chemistry, Kaunas University of Technology, Radvilėnų pl. 19, LT-50254 Kaunas, Lithuania

[2]Laboratory of Drug Targets Histopathology, Institute of Cardiology, Lithuanian University of Health Sciences, Sukilėlių pr. 13, LT-50162 Kaunas, Lithuania; vilmapetrikaite@gmail.com

[3]Institute of Physiology and Pharmacology, Faculty of Medicine, Lithuanian University of Health Sciences, A. Mickevičiaus g. 9, LT-44307 Kaunas, Lithuania

Chemistry

General procedure for the synthesis of *3-((2-oxopropyl)amino)benzenesulfonamide* (2). A mixture of amine **1** (1.0 g, 5.8 mmol), 1-chloropropanone (0.5 ml, 6.2 mmol), 10 ml of water was stirred at reflux for 4 h. Then the reaction mixture was cooled down and the precipitate was filtered off, washed with water, diethyl ether to afford yellow solid, yield 0.58 g (44%); m.p. 114–115 °C; IR (KBr) (ν, cm^{-1}): 3396, 3263, 1724; ^1H NMR (400 MHz, DMSO-d_6) δ (ppm): 2.14 (s, 3H, CH_3), 4.02 (s, 2H, CH_2), 6.33 (br. s, 1H, NH), 6.73 (d, 1H, *J* = 8.2 Hz, H_{ar}), 6.99 (br. s, 2H, NH_2), 7.12–7.27 (m, 3H, H_{ar}); ^{13}C NMR (101 MHz, DMSO-d_6) (δ, ppm): 27.09, 52.79, 108.71, 112.90, 114.95, 129.30, 144.78, 148.39, 205.94; Anal. Calcd. for $C_9H_{12}N_2O_3S$: C 47.36; H 5.30; N 12.27; %. Found: C 47.22; H 5.19; N 12.46 %.

General procedure for the synthesis of *3-(2-mercapto-4-methyl-1H-imidazol-1-yl)benzenesulfonamide* (3). A mixture of compound **2** (0.5 g, 2.2 mmol), KSCN (0.78 g, 8 mmol), 5 ml of glacial acetic acid and 1 ml of HCl was heated at reflux for 4 h. Then the reaction mixture was cooled down and diluted with 20 ml of water. The precipitate was filtered off, washed with water, dried and recrystallized from propan-2-ol to afford brown solid, yield 0.52 g (88%); m.p. 208–209 °C; IR (KBr) (ν, cm^{-1}): 3319, 2664, 1489; ^1H NMR (400 MHz, DMSO-d_6) δ (ppm): 2.08 (s, 3H, CH_3), 7.04 (s, 1H, H_{ar}), 7.47 (br. s, 2H, NH_2), 7.62–7.93 (m, 3H, H_{ar}), 8.10 (s, 1H, CH), 12.39 (s, 1H, SH); ^{13}C NMR (101 MHz, DMSO-d_6) (δ, ppm): 9.74, 115.26, 122.60, 124.46, 125.07, 128.82, 129.54, 138.10, 144.61, 161.29; Anal. Calcd. for $C_{10}H_{11}N_3O_2S_2$: C 44.59; H 4.12; N 15.60; %. Found: C 44.42; H 3.99; N 15.76 %.

General procedure for the synthesis of compounds 4–9. Amine **1** (1.72 g, 10 mmol) was dissolved in boiling water (40 ml). Then the solution of corresponding α-haloketone (12 mmol) in 10 ml of 1,4-dioxane was added dropwise to the mixture. The reaction mixture was heated at reflux for 2 h, then it was cooled down and the precipitate was filtered off, washed with diethyl ether, and recrystallized from 1,4-dioxane to afford compounds **4–9**.

***3-((2-oxo-2-phenylethyl)amino)benzenesulfonamide* (4).** White solid, yield 2.53 g (87%); m.p. 227–228 °C; IR (KBr) (ν, cm^{-1}): 3384, 3260, 1686; ^1H NMR (400 MHz, DMSO-d_6) δ (ppm): 4.75 (s, 2H, CH_2), 6.20 (br. s, 1H, NH), 6.86 (dd, 1H, , *J* = 8.2, 2.3, H_{ar}), 6.99–7.07 (m, 1H, H_{ar}), 7.09–7.29 (m, 4H, NH_2, H_{ar}), 7.53–7.74 (m, 3H, H_{ar}), 8.10 (d, 2H, *J* = 7.2 Hz, H_{ar}); ^{13}C NMR (101 MHz, DMSO-d_6) (δ, ppm): 50.24, 109.08, 113.10, 115.27, 123.88, 129.27, 129.39, 139.73, 144.77, 148.41, 150.13, 195.86; Anal. Calcd. for $C_{14}H_{14}N_2O_3S$: C 57.92; H 4.86; N 9.65 %. Found: C 57.88; H 4.77; N 9.50 %.

***3-((2-(4-chlorophenyl)-2-oxoethyl)amino)benzenesulfonamide* (5).** White solid, yield 2.91 g (90%); m.p. 230–231 °C; IR (KBr) (ν, cm^{-1}): 3372, 3248, 1693; ^1H NMR (400 MHz, DMSO-d_6) δ (ppm): 4.73 (s, 2H, CH_2), 6.39 (br. s, 1H, NH), 6.84 (d, 2H, *J* = 7.8 Hz, H_{ar}), 7.02 (d, 1H, *J* = 7.6 Hz, H_{ar}), 7.11 (br. s, 1H, H_{ar}), 7.18 (br. s, 2H, NH_2), 7.23 (t, 1H, *J* = 7.9 Hz, H_{ar}), 7.64 (d, 2H, *J* = 8.3 Hz, H_{ar}), 8.08 (d, 2H, *J* = 7.2 Hz, H_{ar}); ^{13}C NMR (101 MHz, DMSO-d_6) (δ, ppm): 49.69, 109.02, 112.98, 115.29, 128.93, 129.23, 129.84, 133.73, 138.48, 144.75, 148.50, 195.45; Anal. Calcd. for $C_{14}H_{13}ClN_2O_3S$: C 51.77; H 4.03; N 8.63 %. Found: C 51.71; H 4.07; N 8.58 %.

***3-((2-(3,4-dichlorophenyl)-2-oxoethyl)amino)benzenesulfonamide* (6).** White solid, yield 3.07 g (85%); m.p. 220–221 °C; IR (KBr) (ν, cm^{-1}): 3375, 3263, 1692; ^1H NMR (400 MHz, DMSO-d_6) δ (ppm): 4.76 (s, 2H, CH_2), 6.77–7.33 (m, 6H, NH_2, H_{ar}), 7.84 (d, H, *J* = 8.4 Hz, H_{ar}), 8.01 (d, H, *J* = 8.4 Hz, H_{ar}), 8.28 (s, 1H, H_{ar}); ^{13}C NMR (101 MHz, DMSO-d_6) (δ, ppm): 49.87, 109.12, 113.04, 115.27, 127.93, 129.24, 129.93, 131.15, 131.89, 135.20, 136.36, 144.75, 148.44, 194.84; Anal. Calcd. for $C_{14}H_{12}Cl_2N_2O_3S$: C 46.81; H 3.37; N 7.80 %. Found: C 46.82; H 3.27; N 7.87%.

***3-((2-(4-fluorophenyl)-2-oxoethyl)amino)benzenesulfonamide* (7).** White solid, yield 3.00 g (97%); m.p. 228–229 °C; IR (KBr) (ν, cm^{-1}): 3387, 3256, 1687; ^1H NMR (400 MHz, DMSO-d_6) δ (ppm): 4.73 (s, 2H, CH_2), 6.39 (br. s, 1H, NH), 6.84 (d, 1H, *J* = 8.2 Hz, H_{ar}), 7.01 (d, 1H, *J* = 7.6 Hz, H_{ar}), 7.12 (br. s, 1H, H_{ar}), 7.18 (br. s, 2H, NH_2), 7.23 (t, 1H, *J* = 7.9 Hz, H_{ar}), 7.35–7.44 (m, 2H, H_{ar}), 8.12–8.19 (m, 2H, H_{ar}); ^{13}C NMR (101 MHz, DMSO-d_6) (δ, ppm): 49.60, 109.01, 112.94, 115.27, 115.75, 115.96, 129.23, 130.90, 130.99, 131.81, 131.84, 144.75, 148.55, 194.96; Anal. Calcd. for $C_{14}H_{13}FN_2O_3S$: C 54.54; H 4.25; N 9.09 %. Found: C 54.57; H 4.41; N 8.95 %.

***3-((2-(4-cyanophenyl)-2-oxoethyl)amino)benzenesulfonamide* (8).** Yellow solid, yield 2.40 g (76%); m.p. 219–220 °C; IR (KBr) (ν, cm^{-1}): 3377, 3252, 2228, 1691; ^1H NMR (400 MHz, DMSO-d_6) δ (ppm): 4.79 (s, 2H, CH_2), 6.34 (br. s,

1H, NH), 6.85 (d, 1H, J = 8.2 Hz, H_{ar}), 7.02 (d, 1H, J = 6.8 Hz, H_{ar}) 7.12 (br. s, 1H, H_{ar}), 7.15–7.27 (m, 3H, NH_2, H_{ar}), 8.06 (d, 2H, J = 8.1 Hz, H_{ar}), 8.21 (d, 2H, J = 8.1 Hz, H_{ar}); ^{13}C NMR (101 MHz, DMSO-d_6) (δ, ppm): 50.07, 109.08, 113.05, 115.26, 115.46, 118.16, 128.57, 129.25, 132.84, 138.26, 144.76, 148.43, 196.04; Anal. Calcd. for $C_{15}H_{13}N_3O_3S$: C 57.13; H 4.16; N 13.33 %. Found: C 57.08; H 4.26; N 13.29 %.

3-((2-(4-nitrophenyl)-2-oxoethyl)amino)benzenesulfonamide (9). Orange solid, yield 3.28 g (98%); m.p. 217–218 °C; IR (KBr) (ν, cm^{-1}): 3344, 3262, 1702; ^{1}H NMR (400 MHz, DMSO-d_6) δ (ppm): 4.82 (s, 2H, CH_2), 6.46 (br. s, 1H, NH), 6.86 (d, 2H, J = 8.2 Hz, H_{ar}), 7.03 (d, 1H, J = 7.7 Hz, H_{ar}), 7.13 (br. s, 1H, H_{ar}), 7.18 (br. s, 2H, NH_2), 7.24 (t, 1H, J = 7.9 Hz, H_{ar}), 8.19–8.49 (m, 4H, H_{ar}); ^{13}C NMR (101 MHz, DMSO-d_6) (δ, ppm): 50.24, 109.08, 113.10, 115.27, 123.88, 129.77, 129.39, 139.73, 144.77, 148.41, 150.13, 195.86; Anal. Calcd. for $C_{14}H_{13}N_3O_5S$: C 50.15; H 3.91; N 12.53; %. Found: C 50.28; H 4.04; N 12.33 %.

General procedure for the synthesis of imidazoles 10–15. 2 mmol of compounds **4–9** was dissolved in the solution of glacial acetic acid (5 ml) and HCl (1 ml), and KSCN (0.78 g, 8 mmol) was added. The reaction mixture was heated at reflux for 4 h, then it was cooled down, diluted with water and the precipitate was filtered off, washed with water and n-hexane.

3-(2-mercapto-4-phenyl-1H-imidazol-1-yl)benzenesulfonamide (10). White solid, yield 0.48 g (72%); m.p. 216–217 °C; IR (KBr) (ν, cm^{-1}): 3358, 2770, 1552; ^{1}H NMR (400 MHz, DMSO-d_6) δ (ppm): 7.33 (t, 1H, J = 7.5 Hz, H_{ar}), 7.39–7.48 (m, 1H, H_{ar},), 7.52 (s, 2H, NH_2), 7.70–8.00 (m, 6H, H_{ar}), 8.20 (s, 1H, H_{ar}), 13.08 (s, 1H, SH); ^{13}C NMR (101 MHz, DMSO-d_6) (δ, ppm): 115.69, 123.02, 124.30, 124.94, 127.57, 128.02, 128.71, 128.95, 129.26, 129.66, 137.90, 144.74, 162.94; Anal. Calcd. for $C_{15}H_{13}N_3O_2S_2$: C 54.36; H 3.95; N 12.68 %. Found: C 54.24; H 3.77; N 12.52 %.

3-(4-(4-chlorophenyl)-2-mercapto-1H-imidazol-1-yl)benzenesulfonamide (11). Light-brown solid, yield 0.52 g (71%); m.p. 277–278 °C; IR (KBr) (ν, cm^{-1}): 3340, 2728, 1568; ^{1}H NMR (400 MHz, DMSO-d_6) δ (ppm): 7.44–7.58 (m, 4H, H_{ar}, NH_2), 7.67–7.81 (m, 3H, H_{ar}), 7.90 (d, 1H, J = 8.0 Hz, H_{ar}), 7.95 (d, 1H, J = 8.0 Hz, H_{ar}), 8.00 (br. s, 2H, H_{ar}), 8.19 (s, 1H, CH), 13.12 (s, 1H, SH); ^{13}C NMR (101 MHz, DMSO-d_6) (δ, ppm): 116.33, 123.00, 125.02, 126.01, 126.52, 127.63, 129.00, 129.25, 129.69, 132.41, 137.81, 144.77, 163.15; Anal. Calcd. for $C_{15}H_{12}ClN_3O_2S_2$: C 49.25; H 3.31; N 11.49; %. Found: C 49.44; H 3.22; N 11.40 %.

3-(4-(3,4-dichlorophenyl)-2-mercapto-1H-imidazol-1-yl)benzenesulfonamide (12). Light-brown solid, yield 0.58 g (73%); m.p. 241–242 °C; IR (KBr) (ν, cm^{-1}): 3364, 2720, 1493; ^{1}H NMR (400 MHz, DMSO-d_6) δ (ppm): 7.53 (br. s, 2H, NH_2), 7.70–7.83 (m, 3H, H_{ar}), 7.89 (d, 1H, J = 7.9 Hz, H_{ar}), 7.95 (d, 1H, J = 8.0 Hz, H_{ar}), 8.00 (s, 1H, H_{ar}), 8.18 (s, 1H, CH), 13.16 (s, 1H, SH); ^{13}C NMR (101 MHz, DMSO-d_6) (δ, ppm): 117.39, 122.90, 124.23, 125.09, 125.94, 126.38, 128.21, 129.18, 129.69, 130.06, 131.13, 131.90, 137.68, 144.80, 163.41; Anal. Calcd. for $C_{15}H_{12}ClN_3O_2S_2$: C 49.25; H 3.31; N 11.49 %. Found: C 49.26; H 3.35; N 11.42 %.

3-(4-(4-fluorophenyl)-2-mercapto-1H-imidazol-1-yl)benzenesulfonamide (13). Light-yellow solid, yield 0.48 g (69%); m.p. 271–272 °C; IR (KBr) (ν, cm^{-1}): 3338, 2729, 1562; ^{1}H NMR (400 MHz, DMSO-d_6) δ (ppm): 7.27 (t, 2H, J = 8.8 Hz, H_{ar}), 7.45 (br. s, 2H, NH_2), 7.62–7.75 (m, 5H, H_{ar}), 7.95 (d, 1H, J = 7.4 Hz, H_{ar}), 8.43 (s, 1H, CH), 11.24 (s, 1H, SH); ^{13}C NMR (101 MHz, DMSO-d_6) (δ, ppm): 106.15, 115.72, 115.94, 117.33, 121.96, 122.11, 123.01, 125.47, 125.50, 125.55, 129.86, 137.46, 145.04, 152.31, 160.10, 162.53; Anal. Calcd. for $C_{15}H_{12}FN_3O_2S_2$: C 51.56; H 3.46; N 12.03 %. Found: C 51.36; H 3.29; N 12.10 %.

3-(4-(4-cyanophenyl)-2-mercapto-1H-imidazol-1-yl)benzenesulfonamide (14). Light-yellow solid, yield 0.55 g (77%); m.p. 301–302 °C; IR (KBr) (ν, cm^{-1}): 3344, 2815, 2232, 1510; ^{1}H NMR (400 MHz, DMSO-d_6) δ (ppm): 7.53 (br. s, 2H, NH_2), 7.76 (t, 1H, J = 8.0 Hz, H_{ar}), 7.85–7.99 (m, 6H, H_{ar}), 8.18 (s, 1H, CH), 8.21 (s, 1H, H_{ar}), 13.27 (s, 1H, SH); ^{13}C NMR (101 MHz, DMSO-d_6) (δ, ppm): 109.85, 118.51, 118.75, 123.09, 124.63, 125.22, 127.04, 129.36, 129.75, 131.91, 132.96, 137.63, 144.81, 163.84; Anal. Calcd. for $C_{16}H_{12}N_4O_2S_2$: C 53.92; H 3.39; N 15.72 %. Found: C 53.71; H 3.34; N 15.79 %.

3-(2-mercapto-4-(4-nitrophenyl)-1H-imidazol-1-yl)benzenesulfonamide (15). Dark-yellow solid, yield 0.58 g (77%); m.p. 278–279 °C; IR (KBr) (ν, cm^{-1}): 3311, 2719, 1516; ^{1}H NMR (400 MHz, DMSO-d_6) δ (ppm): 7.54 (br. s, 2H,

NH_2), 7.77 (t, 1H, *J* = 7.9 Hz, H_{ar}), 7.88–8.08 (m, 4H, H_{ar}), 8.19 (s, 1H, CH), 8.28, 8.30 (2s, 3H, H_{ar}), 13.35 (s, 1H, SH); ^{13}C NMR (101 MHz, DMSO-*d*$_6$) (δ, ppm): 119.24, 123.09, 124.39, 124.87, 125.27, 126.73, 129.36, 129.75, 133.86, 137.56, 144.82, 146.17, 164.10; Anal. Calcd. for $C_{15}H_{12}N_4O_4S_2$: C 47.86; H 3.21; N 14.89; %. Found: C 47.93; H 3.19; N 14.64 %.

General procedure for the synthesis of *3-(4-(4-chlorophenyl)-2-(ethylthio)-1H-imidazol-1-yl)benzenesulfonamide* (16). Imidazole **11** (0.37 g, 1.0 mmol) was dissolved in DMF (3 ml). Triethylamine (0.5 ml) and ethyl iodide (0.24 g, 1.5 mmol) were added dropwise, and the reaction mixture was stirred at room temperature for 3 h. Then the reaction mixture diluted with 20 ml of water. The precipitate was filtered off, washed with water, diethyl ether, dried and recrystallized from propan-2-ol to afford white solid, yield 0.36 g (92%); m.p. 214–215 °C; IR (KBr) (ν, cm^{-1}): 3349, 1481; ^{1}H NMR (400 MHz, DMSO-*d*$_6$) δ (ppm): 1.30 (t, 3H, *J* = 7.3 Hz, CH_3), 3.15 (q, 2H, *J* = 7.4 Hz, CH_2), 7.46 (d, 2H, *J* = 8.2 Hz, H_{ar}), 7.58 (br. s, 2H, NH_2), 7.72–8.01 (m, 6H, H_{ar}), 8.14 (s, 1H, CH); ^{13}C NMR (101 MHz, DMSO-*d*$_6$) (δ, ppm): 14.83, 27.52, 119.34, 122.34, 125.51, 126.02, 128.62, 130.48, 131.15, 132.36, 136.88, 140.31, 142.19, 145.39; Anal. Calcd. for $C_{17}H_{16}ClN_3O_2S_2$: C 51.84; H 4.09; N 10.67 %. Found: C 51.71; H 3.89; N 10.76 %.

General procedure for the synthesis of imidazoles 17–22. 2 mmol of compounds **4–9** was dissolved in glacial acetic acid (5 ml) and urea (0.48 g, 8 mmol) was added. The reaction mixture was heated at reflux for 12 h, then it was cooled down, diluted with water and the precipitate was filtered off, washed with water and n-hexane.

***3-(2-oxo-4-phenyl-2,3-dihydro-1H-imidazol-1-yl)benzenesulfonamide* (17).** White solid, yield 0.31 g (49%); m.p. 258–259 °C; IR (KBr) (ν, cm^{-1}): 3332, 3241, 1703; ^{1}H NMR (400 MHz, DMSO-*d*$_6$) δ (ppm): 7.27 (t, 1H, *J* = 7.4 Hz, H_{ar}), 7.36–7.49 (m, 4H, H_{ar}, NH_2), 7.63–7.74 (m, 5H, H_{ar}), 7.97 (d, 1H, *J* = 7.2 Hz, H_{ar}), 8.44 (s, 1H, CH), 11.23 (s, 1H, NH); ^{13}C NMR (101 MHz, DMSO-*d*$_6$) (δ, ppm): 106.27, 117.36, 121.94, 122.93, 123.05, 123.45, 127.31, 128.82, 129.86, 137.49, 145.03, 152.34; Anal. Calcd. for $C_{15}H_{13}N_3O_3S$: C 57.13; H 4.16; N 13.33; %. Found: C 57.41; H 4.08; N 13.11 %.

***3-(4-(4-chlorophenyl)-2-oxo-2,3-dihydro-1H-imidazol-1-yl)benzenesulfonamide* (18).** Light-brown solid, yield 0.36 g (52%); m.p. 300–301 °C; IR (KBr) (ν, cm^{-1}): 3322, 3249, 1691; ^{1}H NMR (400 MHz, DMSO-*d*$_6$) δ (ppm): 7.43–7.53 (m, 4H, H_{ar}, NH_2), 7.63–7.74 (m, 4H, H_{ar}), 7.78 (s, 1H, H_{ar}), 7.95 (d, 1H, *J* = 7.5 Hz, H_{ar}), 8.42 (s, 1H, CH), 11.27 (s, 1H, NH); ^{13}C NMR (101 MHz, DMSO-*d*$_6$) (δ, ppm): 107.09, 117.40, 121.87, 122.07, 123.09, 124.38, 125.10, 127.79, 128.61, 128.85, 129.87, 131.56, 137.37, 145.05, 152.27; Anal. Calcd. for $C_{15}H_{12}ClN_3O_3S$: C 51.51; H 3.46; N 12.01; %. Found: C 51.33; H 3.27; N 11.92 %.

***3-(4-(3,4-dichlorophenyl)-2-oxo-2,3-dihydro-1H-imidazol-1-yl)benzenesulfonamide* (19).** Light-brown solid, yield 0.34 g (44%); m.p. 286–287 °C; IR (KBr) (ν, cm^{-1}): 3331, 3245, 1704; ^{1}H NMR (400 MHz, DMSO-*d*$_6$) δ (ppm): 7.45 (br. s, 2H, NH_2), 7.61–7.73 (m, 4H, H_{ar}), 7.90–8.00 (m, 3H, H_{ar}), 8.40 (s, 1H, CH), 11.31 (s, 1H, NH); ^{13}C NMR (101 MHz, DMSO-*d*$_6$) (δ, ppm): 108.44, 117.39, 120.66, 122.23, 123.12, 123.35, 125.02, 129.14, 129.57, 129.90, 131.00, 131.78, 137.23, 145.08, 152.14; Anal. Calcd. for $C_{15}H_{11}Cl_2N_3O_3S$: C 46.89; H 2.89; N 10.94; %. Found: C 46.69; H 2.82; N 10.84 %.

***3-(4-(4-fluorophenyl)-2-oxo-2,3-dihydro-1H-imidazol-1-yl)benzenesulfonamide* (20).** Yellow solid, yield 0.39 g (59%); m.p. 298–299 °C; IR (KBr) (ν, cm^{-1}): 3320, 3248, 1691; ^{1}H NMR (400 MHz, DMSO-*d*$_6$) δ (ppm): 7.27 (t, 2H, *J* = 8.7 Hz, H_{ar}), 7.45 (br. s, 2H, NH_2), 7.60–7.76 (m, 5H, H_{ar}), 7.95 (d, 1H, *J* = 7.4 Hz, H_{ar}), 8.43 (s, 1H, CH), 11.24 (s, 1H, NH); ^{13}C NMR (101 MHz, DMSO-*d*$_6$) (δ, ppm): 106.15, 106.17, 115.72, 115.94, 117.33, 121.97, 122.12, 123.01, 125.47, 125.50, 125.56, 129.87, 137.47, 1145.05, 152.31; Anal. Calcd. for $C_{15}H_{12}FN_3O_3S$: C 54.05; H 3.63; N 12.61; %. Found: C 53.87; H 3.63; N 12.44 %.

***3-(4-(4-cyanophenyl)-2-oxo-2,3-dihydro-1H-imidazol-1-yl)benzenesulfonamide* (21).** Yellow solid, yield 0.29 g (43%); m.p. 328–329 °C; IR (KBr) (ν, cm^{-1}): 3299, 3219, 2226, 1704 ^{1}H NMR (400 MHz, DMSO-*d*$_6$) δ (ppm): 7.47 (br. s, 2H, NH_2), 7.65–7.75 (m, 2H, H_{ar}), 7.80–7.89 (m, 4H, H_{ar}, CH), 7.95 (d, 1H, *J* = 7.8 Hz, H_{ar}), 8.01 (s, 1H, H_{ar}), 8.41 (s, 1H, CH), 11.42 (s, 1H, NH); ^{13}C NMR (101 MHz, DMSO-*d*$_6$) (δ, ppm): 108.95, 109.85, 117.68, 118.94, 121.40, 122.44, 123.40, 123.73, 129.94, 132.81, 133.26, 137.13, 145.09, 152.26; Anal. Calcd. for $C_{16}H_{12}N_4O_3S$: C 56.46; H 3.55; N 16.46 %. Found: C 56.30; H 3.60; N 16.46 %.

3-(4-(4-nitrophenyl)-2-oxo-2,3-dihydro-1H-imidazol-1-yl)benzenesulfonamide (22). Brown solid, yield 0.28 g (38%); m.p. 246–247 °C; IR (KBr) (ν, cm^{-1}): 3307, 3231, 1693; ^{1}H NMR (400 MHz, DMSO-*d6*) δ (ppm): ^{1}H NMR (400 MHz, DMSO-*d6*) δ (ppm): 7.38–7.48 (m, 4H, NH$_2$, H$_{ar}$), 7.58–7.69 (m, 4H, H$_{ar}$), 7.73 (s, 1H, H$_{ar}$), 7.90 (d, 1H, *J* = 7.5 Hz, H$_{ar}$), 8.37 (s, 1H, CH), 11.22 (s, 1H, NH); ^{13}C NMR (101 MHz, DMSO-*d6*) (δ, ppm): 113.96, 114.57, 118.11, 120.51, 122.48, 123.70, 123.90, 124.26, 129.36, 133.45, 139.34, 139.47, 147.67, 155.62; Anal. Calcd. for $C_{15}H_{12}N_4O_5S$: C 50.00; H 3.36; N 15.55; %. Found: C 49.85; H 3.13; N 15.38 %.

General procedure for the synthesis of ***3-(3-ethyl-4-(4-fluorophenyl)-2-oxo-2,3-dihydro-1H-imidazol-1-yl)benzenesulfonamide*** (**23**). Imidazole **20** (0.35 g, 1.0 mmol) was dissolved in DMF (3 ml). Triethylamine (0.5 ml) and ethyl iodide (0.24 g, 1.5 mmol) were added dropwise, and the reaction mixture was stirred at room temperature for 10 hours. Then the reaction mixture was diluted with 20 ml of water. The precipitate was filtered off, washed with water, diethyl ether, dried and recrystallized from propan-2-ol to afford white solid, yield 0.26 g (72%); m.p. 177–178 °C; IR (KBr) (ν, cm^{-1}): 3350, 1671; ^{1}H NMR (400 MHz, DMSO-*d6*) δ (ppm): 1.30 (t, 3H, *J* = 7.3 Hz, CH$_3$), 3.14 (q, 2H, *J* = 7.3 Hz, CH$_2$), 7.23 (t, 2H, *J* = 8.7 Hz, H$_{ar}$), 7.57 (br. s, 2H, NH$_2$), 7.70–8.00 (m, 6H, CH, H$_{ar}$), 8.08 (s, 1H, CH); ^{13}C NMR (101 MHz, DMSO-*d6*) (δ, ppm): 14.83, 27.56, 115.34, 115.56, 118.69, 122.32, 125.44, 126.21, 126.29, 128.63, 130.00, 130.03, 130.47, 136.94, 140.57, 141.88, 145.36; Anal. Calcd. for $C_{17}H_{16}FN_3O_3S$: C 56.50; H 4.46; N 11.63; %. Found: C 56.40; H 4.24; N 11.38 %.

General procedure for the synthesis of compounds 25–30. Amine **24** (1.88 g, 10 mmol) was dissolved in boiling water (30 ml). Then the solution of corresponding α-haloketone (12 mmol) in 10 ml of 1,4-dioxane was added dropwise to the mixture. The reaction mixture was heated at reflux for 2 h, then it was cooled down and the precipitate was filtered off, washed with diethyl ether, and recrystallized from 1,4-dioxane to afford compounds **25–30**.

4-hydroxy-3-((2-oxo-2-phenylethyl)amino)benzenesulfonamide (25). Yellowish solid, yield 2.81 g (92%); m.p. 219–220 °C; IR (KBr) (ν, cm^{-1}): 3426, 3319, 3238, 1681; ^{1}H NMR (400 MHz, DMSO-*d6*) δ (ppm): 4.74 (d, 2H, *J* = 7.7 Hz, CH$_2$), 5.45 (t, 1H, *J* = 5.3 Hz, NH), 6.79 (d, 1H, *J* = 7.9 Hz, H$_{ar}$), 6.97, 7.00 (2s, 4H, H$_{ar}$, NH$_2$), 7.50–7.77 (m, 3H, H$_{ar}$), 8.09 (d, 2H, *J* = 7.7 Hz, H$_{ar}$), 10.33 (s, 1H, OH); ^{13}C NMR (101 MHz, DMSO-*d6*) (δ, ppm): 49.69, 107.08, 112.17, 114.59, 127.87, 128.89, 133.77, 134.87, 135.25, 136.67, 147.04, 195.81; Anal. Calcd. for $C_{14}H_{14}N_2O_4S$: C 54.89; H 4.61; N 9.14 %. Found: C 54.80; H 4.51; N 9.16 %.

3-((2-(4-chlorophenyl)-2-oxoethyl)amino)-4-hydroxybenzenesulfonamide (26). Brown solid, yield 3.07 g (90%); m.p. 224–225 °C; IR (KBr) (ν, cm^{-1}): 3418, 3320, 3236, 1674; ^{1}H NMR (400 MHz, DMSO-*d6*) δ (ppm): 4.73 (s, 2H, CH$_2$), 5.43 (br. s, 1H, NH), 6.79 (d, 1H, *J* = 8.6 Hz, H$_{ar}$), 6.97, 6.99 (2s, 4H, H$_{ar}$, NH$_2$), 7.65 (d, 2H, *J* = 8.3 Hz, H$_{ar}$), 8.10 (d, 2H, *J* = 8.3 Hz, H$_{ar}$), 10.32 (s, 1H, OH); ^{13}C NMR (101 MHz, DMSO-*d6*) (δ, ppm): 49.77, 107.10, 112.21, 114.66, 128.98, 129.83, 133.57, 135.24, 136.59, 138.61, 147.03, 195.04; Anal. Calcd. for $C_{14}H_{13}ClN_2O_4S$: C 49.34; H 3.85; N 8.22 %. Found: C 49.12; H 3.83; N 8.29 %.

3-((2-(3,4-dichlorophenyl)-2-oxoethyl)amino)-4-hydroxybenzenesulfonamide (27). Yellow solid, yield 2.68 g (71%); m.p. 230–231 °C; IR (KBr) (ν, cm^{-1}): 3418, 3310, 3234, 1687; ^{1}H NMR (400 MHz, DMSO-*d6*) δ (ppm): 4.75z (s, 2H, CH$_2$), 5.42 (br. s, 1H, NH), 6.78 (d, 1H, *J* = 8.2 Hz, H$_{ar}$), 6.90–7.05 (m, 4H, NH$_2$, H$_{ar}$), 7.85 (d, 1H, *J* = 8.4 Hz, H$_{ar}$), 8.03 (d, 1H, *J* = 6.5 Hz, H$_{ar}$), 8.30 (s, 1H, H$_{ar}$), 10.33 (s, 1H, OH); ^{13}C NMR (101 MHz, DMSO-*d6*) (δ, ppm): 49.98, 107.14, 112.22, 114.73, 127.94, 129.90, 131.20, 131.95, 135.06, 135.25, 136.47, 136.54, 147.03, 194.50; Anal. Calcd. for $C_{14}H_{12}Cl_2N_2O_4S$: C 44.81; H 3.22; N 7.47 %. Found: C 45.02; H 3.31; N 7.40 %.

3-((2-(4-fluorophenyl)-2-oxoethyl)amino)-4-hydroxybenzenesulfonamide (28). White solid, yield 3.01 g (93%); m.p. 222–223 °C; IR (KBr) (ν, cm^{-1}): 3419, 3313, 3237, 1685; ^{1}H NMR (400 MHz, DMSO-*d6*) δ (ppm): 4.73 (s, 2H, CH$_2$), 5.43 (br. s, 1H, NH), 6.79 (d, 1H, *J* = 8.5 Hz, H$_{ar}$), 6.97, 7.00 (2s, 4H, H$_{ar}$, NH$_2$), 7.36–7.46 (m, 2H, H$_{ar}$), 8.13–8.22 (m, 2H, H$_{ar}$), 10.33 (s, 1H, OH); ^{13}C NMR (101 MHz, DMSO-*d6*) (δ, ppm): 49.68 107.09, 112.21, 114.64, 115.83, 116.04, 130.91, 131.00, 131.65, 131.67, 135.25, 136.65, 147.04, 194.57; Anal. Calcd. for $C_{14}H_{13}FN_2O_4S$: C 51.85; H 4.04; N 8.64; %. Found: C 51.96; H 4.18; N 8.43 %.

3-((2-(4-cyanophenyl)-2-oxoethyl)amino)-4-hydroxybenzenesulfonamide (29). Yellow solid, yield 3.00 g (91%); m.p. 221–222 °C; IR (KBr) (ν, cm^{-1}): 3422, 3374, 3258, 2255, 1702; ^{1}H NMR (400 MHz, DMSO-*d$_6$*) δ (ppm): 4.78 (s, 2H, CH_2), 5.38 (br. s, 1H, NH), 6.79 (d, 1H, *J* = 8.6 Hz, H_{ar}), 6.90–7.16 (m, 4H, NH_2, H_{ar}), 8.15 (dd, 4H, *J* = 64.4, 8.2 Hz, H_{ar}), 10.33 (s, 1H, OH); ^{13}C NMR (101 MHz, DMSO-*d$_6$*) (δ, ppm): 50.20, 107.12, 112.25, 114.75, 115.56, 118.15, 128.57, 132.89, 135.24, 136.53, 138.10, 147.05, 195.66; Anal. Calcd. for $C_{15}H_{13}N_3O_4S$: C 54.37; H 3.95; N 12.68 %. Found: C 54.62; H 4.04; N 12.46 %. C, 54.37; H, 3.95; N, 12.68 %.

4-hydroxy-3-((2-(4-nitrophenyl)-2-oxoethyl)amino)benzenesulfonamide (30). Orange solid, yield 3.37 g (96%); m.p. 189–190 °C; IR (KBr) (ν, cm^{-1}): 3414, 3340, 3250, 1685; ^{1}H NMR (400 MHz, DMSO-*d$_6$*) δ (ppm): 4.81 (s, 2H, CH_2), 5.45 (br. s, 1H, NH), 6.79 (d, 1H, *J* = 8.6 Hz, H_{ar}), 6.91–7.02 (m, 4H, NH_2, H_{ar}), 8.26–8.42 (m, 4H, H_{ar}), 10.34 (s, 1H, OH); ^{13}C NMR (101 MHz, DMSO-*d$_6$*) (δ, ppm): 50.35, 107.11, 112.26, 114.78, 123.93, 129.39, 135.24, 136.48, 139.55, 147.04, 150.19, 195.46; Anal. Calcd. for $C_{14}H_{13}N_3O_6S$: C 47.86; H 3.73; N 11.96 %. Found: C 47.89; H 3.69; N 12.00 %.

General procedure for the synthesis of imidazoles 31–36. 2 mmol of compounds **25–30** were dissolved in the solution of glacial acetic acid (5 ml) and HCl (1 ml), and KSCN (0.78 g, 8 mmol) was added. The reaction mixture was heated at reflux for 4 h, then it was cooled down, diluted with water and the precipitate was filtered off, washed with water and n-hexane.

4-hydroxy-3-(2-mercapto-4-phenyl-1H-imidazol-1-yl)benzenesulfonamide (31). Brown solid, yield 0.48 g (70%); m.p. 162–163 °C; IR (KBr) (ν, cm^{-1}): 3402, 2735, 1509; ^{1}H NMR (400 MHz, DMSO-*d$_6$*) δ (ppm): 6.70–8.34 (m, 11H, H_{ar}, CH, NH_2), 10.89 (s, 1H, OH), 12.92 (s, 1H, SH); ^{13}C NMR (101 MHz, DMSO-*d$_6$*) (δ, ppm): 116.91, 124.03, 124.62, 127.70, 127.86, 128.07, 128.93, 134.66, 155.77, 163.65; Anal. Calcd. for $C_{15}H_{13}N_3O_3S_2$: C 51.86; H 3.77; N 12.10 %. Found: C 51.80; H 3.72; N 12.10 %.

3-(4-(4-chlorophenyl)-2-mercapto-1H-imidazol-1-yl)-4-hydroxybenzenesulfonamide (32). Light-brown solid, yield 0.52 g (68%); m.p. 268–269 °C; IR (KBr) (ν, cm^{-1}): 3411, 2721, 1493; ^{1}H NMR (400 MHz, DMSO-*d$_6$*) δ (ppm): 7.16 (d, 1H, *J* = 8.6 Hz, H_{ar}), 7.31 (br. s, 2H, NH_2), 7.49 (d, 2H, *J* = 8.2 Hz, H_{ar}), 7.69–7.85 (m, 5H, H_{ar}, CH), 10.91 (s, 1H, OH), 12.96 (s, 1H, SH); ^{13}C NMR (101 MHz, DMSO-*d$_6$*) (δ, ppm): 116.94, 117.52, 124.50, 125.74, 126.84, 127.01, 127.70, 127.92, 128.98, 132.07, 134.67, 155.77, 163.94; Anal. Calcd. for $C_{15}H_{12}ClN_3O_3S_2$: 47.18; H 3.17; N 11.00; %. Found: C 47.49; H 3.05; N 10.82 %.

3-(4-(3,4-dichlorophenyl)-2-mercapto-1H-imidazol-1-yl)-4-hydroxybenzenesulfonamide (33). Light-yellow solid, yield 0.42 g (51%); m.p. 230–231 °C; IR (KBr) (ν, cm^{-1}): 3371, 2752, 1503; ^{1}H NMR (400 MHz, DMSO-*d$_6$*) δ (ppm): 7.18 (d, 1H, *J* = 8.6 Hz, H_{ar}), 7.31 (br. s, 2H, NH_2), 7.60–7.83 (m, 4H, H_{ar}), 7.87 (s, 1H, H_{ar}), 8.07 (s, 1H, CH), 10.98 (s, 1H, OH), 13.01 (s, 1H, SH); ^{13}C NMR (101 MHz, DMSO-*d$_6$*) (δ, ppm): 116.94, 118.62, 124.02, 124.34, 125.63, 125.78, 127.64, 127.97, 128.53, 129.72, 131.12, 131.89, 134.65, 155.75, 164.26; Anal. Calcd. for $C_{15}H_{11}Cl_2N_3O_3S_2$: C 43.28; H 2.66; N 10.09 %. Found: C 43.07; H 2.54; N 9.92 %.

3-(4-(4-fluorophenyl)-2-mercapto-1H-imidazol-1-yl)-4-hydroxybenzenesulfonamide (34). Light-yellow solid, yield 0.49 g (67%); m.p. 277–278 °C; IR (KBr) (ν, cm^{-1}): 3340, 2731, 1501; ^{1}H NMR (400 MHz, DMSO-*d$_6$*) δ (ppm): 7.16 (d, 1H, *J* = 8.6 Hz, H_{ar}), 7.22–7.36 (m, 4H, H_{ar}, NH_2), 7.61–7.84 (m, 5H, H_{ar}, CH), 10.90 (s, 1H, OH), 12.92 (s, 1H, SH); ^{13}C NMR (101 MHz, DMSO-*d$_6$*) (δ, ppm): 115.84, 116.05, 116.78, 116.94, 124.54, 124.57, 126.16, 126.24, 127.24, 127.70, 127.87, 134.67, 155.77, 163.69; Anal. Calcd. for $C_{15}H_{12}FN_3O_3S_2$: C 49.31; H 3.31; N 11.50 %. Found: C 49.37; H 3.38; N 11.60 %.

3-(4-(4-cyanophenyl)-2-mercapto-1H-imidazol-1-yl)-4-hydroxybenzenesulfonamide (35). Yellow solid, yield 0.58 g (78%); m.p. 279–280 °C; IR (KBr) (ν, cm^{-1}): 3322, 2735, 2239, 1504; ^{1}H NMR (400 MHz, DMSO-*d$_6$*) δ (ppm): 7.17 (d, 1H, *J* = 8.6 Hz, H_{ar}), 7.32 (br. s, 2H, NH_2), 7.71–8.05 (m, 7H, H_{ar}, CH), 10.97 (s, 1H, OH), 13.12 (s, 1H, SH); ^{13}C NMR (101 MHz, DMSO-*d$_6$*) (δ, ppm): 109.54, 116.96, 118.82, 119.75, 124.30, 124.36, 126.51, 127.68, 128.08, 132.21, 132.98, 134.70, 155.78, 164.70; Anal. Calcd. for $C_{16}H_{12}N_4O_3S_2$: C 51.60; H 3.25; N 15.04 %. Found: C 51.43; H 3.10; N 15.16 %.

*4-hydroxy-3-(2-mercapto-4-(4-nitrophenyl)-1H-imidazol-1-yl)benzenesulfonamide (**36**).* Dark-orange solid, yield 0.51 g (65%); m.p. 271–272 °C; IR (KBr) (ν, cm^{-1}): 3362, 2730, 1502; ^1H NMR (400 MHz, DMSO-d_6) δ (ppm): ^1H NMR (400 MHz, DMSO-d_6) δ (ppm): 7.18 (d, 1H, *J* = 8.6 Hz, H_{ar}), 7.32 (br. s, 2H, NH_2), 7.72–8.11 (m, 5H, H_{ar}, CH), 8.27 (d, 2H, *J* = 8.6 Hz, H_{ar}), 10.98 (s, 1H, OH), 13.21 (s, 1H, SH); ^{13}C NMR (101 MHz, DMSO-d_6) (δ, ppm): 116.96, 120.51, 124.22, 124.41, 124.57, 126.22, 127.65, 128.11, 134.17, 134.70, 145.95, 155.75, 164.99; Anal. Calcd. for $C_{15}H_{12}N_4O_5S_2$: C 45.91; H 3.08; N 14.28 %. Found: C 45.93; H 3.18; N 14.16 %.

General procedure for the synthesis of imidazoles 37–42. 2 mmol of compounds **25–30** were dissolved in glacial acetic acid (5 ml) and urea (0.48 g, 8 mmol) was added. The reaction mixture was heated at reflux for 12 h, then it was cooled down, diluted with water and the precipitate was filtered off, washed with water and n-hexane.

*4-hydroxy-3-(2-oxo-4-phenyl-2,3-dihydro-1H-imidazol-1-yl)benzenesulfonamide (**37**).* Red solid, yield 0.38 g (51%); m.p. 271–272 °C; IR (KBr) (ν, cm^{-1}): 3273, 1701; ^1H NMR (400 MHz, DMSO-d_6) δ (ppm): 7.13 (d, 1H, *J* = 8.6 Hz, H_{ar}), 7.21–7.29 (m, 4H, H_{ar}, NH_2), 7.34–7.41 (m, 2H, H_{ar}), 7.60 (d, 2H, *J* = 7.8 Hz, H_{ar}), 7.67 (dd, 1H, *J* = 8.6, 2.3 Hz, H_{ar}), 7.84 (s, 1H, CH), 10.84 (s, 1H, OH), 11.11 (s, 1H, NH); ^{13}C NMR (101 MHz, DMSO-d_6) (δ, ppm): 109.71, 117.05, 121.78, 123.16, 123.86, 125.55, 126.33, 126.88, 128.78, 129.19, 134.93, 152.87, 154.82; Anal. Calcd. for $C_{15}H_{13}N_3O_4S$: C 54.37; H 3.95; N 12.68 %. Found: C 54.38; H 3.95; N 12.67 %.

*3-(4-(4-chlorophenyl)-2-oxo-2,3-dihydro-1H-imidazol-1-yl)-4-hydroxybenzenesulfonamide (**38**).* Brown solid, yield 0.39 g (53%); m.p. 280–281 °C; IR (KBr) (ν, cm^{-1}): 3243, 1660; ^1H NMR (400 MHz, DMSO-d_6) δ (ppm): 7.13 (d, 1H, *J* = 8.6 Hz, H_{ar}), 7.26 (br. s, 2H, NH_2), 7.33 (s, 1H, H_{ar}), 7.44 (d, 2H, *J* = 8.4 Hz, H_{ar}), 7.61 (d, 2H, *J* = 8.5 Hz, H_{ar}), 7.67 (dd, 1H, *J* = 8.5, 2.3 Hz, H_{ar}), 7.83 (s, 1H, CH), 10.91, 11.12 (2 br. s, 2H, OH, NH); ^{13}C NMR (101 MHz, DMSO-d_6) (δ, ppm): 110.56, 116.99, 120.68, 123.69, 124.81, 125.68, 126.46, 128.19, 128.81, 131.09, 134.87, 152.83, 154.95; Anal. Calcd. for $C_{15}H_{12}ClN_3O_4S$: C 49.25; H 3.31; N 11.49; %. Found: C 49.05; H 3.26; N 11.34 %.

*3-(4-(3,4-dichlorophenyl)-2-oxo-2,3-dihydro-1H-imidazol-1-yl)-4-hydroxybenzenesulfonamide(**39**).* White solid, yield 0.44 g (55%); m.p. 288–289 °C; IR (KBr) (ν, cm^{-1}): 3322, 1698; ^1H NMR (400 MHz, DMSO-d_6) δ (ppm): 7.14 (d, 1H, *J* = 8.6 Hz, H_{ar}), 7.26 (br. s, 2H, NH_2), 7.47 (s, 1H, H_{ar}), 7.55–7.70 (m, 5H, H_{ar}), 7.82 (d, 1H, *J* = 2.3 Hz, H_{ar}), 7.89 (s, 1H, CH), 10.86 (s, 1H, OH), 11.15, 11.24 (2s, 1H, NH); ^{13}C NMR (101 MHz, DMSO-d_6) (δ, ppm): 111.97. 116.93. 119.49. 123.13. 123.49. 124.66. 125.77. 126.61. 128.66. 129.52. 130.00. 130.94. 131.76. 131.90. 133.17. 134.90. 152.71. 154.97. 155.61; Anal. Calcd. for $C_{15}H_{11}Cl_2N_3O_4S$: C 45.02; H 2.77; N 10.50; %. Found: C 44.86; H 2.62; N 10.54 %.

*3-(4-(4-fluorophenyl)-2-oxo-2,3-dihydro-1H-imidazol-1-yl)-4-hydroxybenzenesulfonamide (**40**).* Dark-red solid, yield 0.49 g (59%); m.p. 246–247 °C; IR (KBr) (ν, cm^{-1}): 3311, 1668; ^1H NMR (400 MHz, DMSO-d_6) δ (ppm): 7.14 (d, 1H, *J* = 8.6 Hz, H_{ar}), 7.19–7.30 (m, 5H, H_{ar}, NH_2), 7.59–7.70 (m, 3H, H_{ar}), 7.83 (s, 1H, CH), 11.11 (br. s, 2H, NH, OH); ^{13}C NMR (101 MHz, DMSO-d_6) (δ, ppm): 109.61, 115.67, 115.88, 117.05 120.94, 123.82, 125.15, 125.23, 125.58, 125.86, 125.89, 126.37, 134.84, 152.86, 154.94, 159.86, 162.28; Anal. Calcd. for $C_{15}H_{12}FN_3O_4S$: C 51.57; H 3.46; N 12.03; %. Found: C 51.78; H 3.51; N 12.04 %.

*3-(4-(4-cyanophenyl)-2-oxo-2,3-dihydro-1H-imidazol-1-yl)-4-hydroxybenzenesulfonamide (**41**).* Yellow solid, yield 0.36 g (51%); m.p. 286–287 °C; IR (KBr) (ν, cm^{-1}): 3323, 2227, 1698; ^1H NMR (400 MHz, DMSO-d_6) δ (ppm): 7.13 (d, 1H, *J* = 8.6 Hz, H_{ar}), 7.26 (s, 2H, NH_2), 7.57 (s, 1H, H_{ar}), 7.65–7.86 (m, 6H, CH, H_{ar}), 11.17 (br. s, 2H, NH, OH); ^{13}C NMR (101 MHz, DMSO-d_6) (δ, ppm): 108.43, 113.47, 116.90, 119.04, 120.13, 123.34, 123.37, 125.93, 126.75, 132.79, 133.70, 134.67, 152.83, 155.30; Anal. Calcd. for $C_{16}H_{12}N_4O_4S$: C 53.93; H 3.39; N 15.72; %. Found: C 54.14; H 3.43; N 15.78 %.

*4-hydroxy-3-(4-(4-nitrophenyl)-2-oxo-2,3-dihydro-1H-imidazol-1-yl)benzenesulfonamide (**42**).* Orange solid, yield 0.38 g (51%); m.p. 280–281 °C; IR (KBr) (ν, cm^{-1}): 3342, 1694; ^1H NMR (400 MHz, DMSO-d_6) δ (ppm): 7.32 (s, 2H, NH_2), 7.72 (d, 1H, *J* = 8.8 Hz, H_{ar}), 8.12–8.22 (m, 4H, H_{ar}), 8.29–8.41 (m, 3H, CH, H_{ar}), 10.50 (s, 1H, OH), 10.80 (s, 1H, NH); ^{13}C NMR (101 MHz, DMSO-d_6) (δ, ppm): 109.72, 113.96, 114.57, 118.11, 120.51, 122.48, 123.90, 124.26,

129.36, 129.93, 139.34, 145.11, 147.67, 155.62; Anal. Calcd. for $C_{15}H_{12}N_4O_6S$: C 47.87; H 3.21; N 14.89; %. Found: C 48.02; H 3.06; N 14.83 %.

NMR data

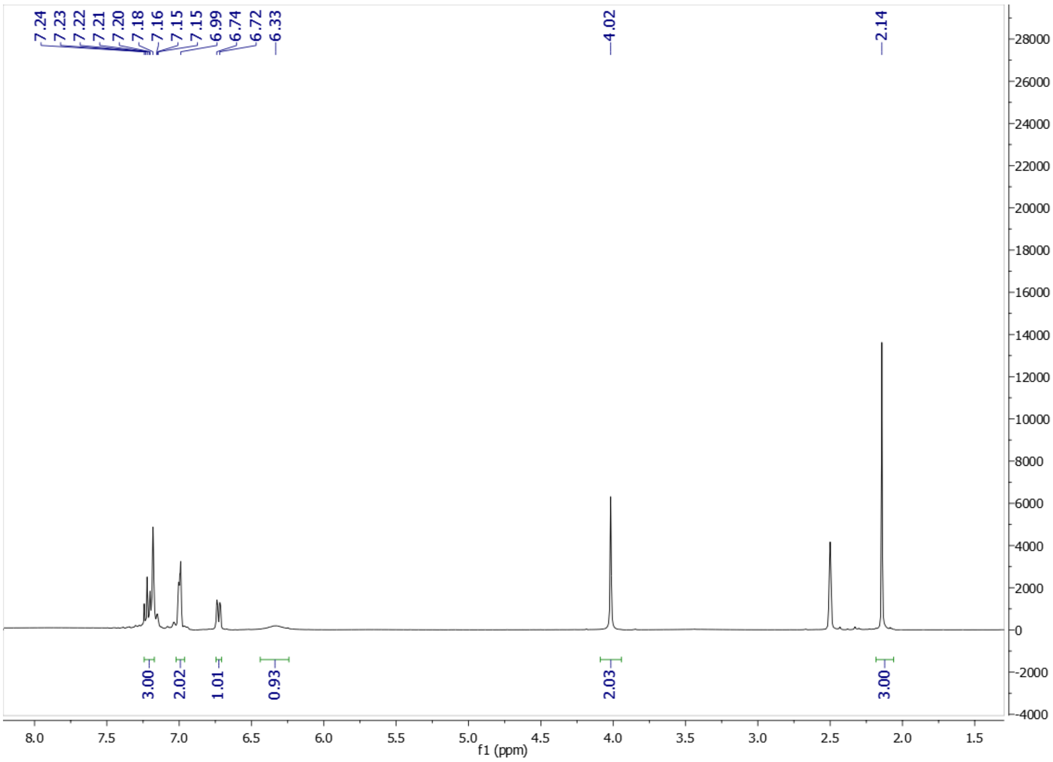

Figure S1. ^{1}H NMR of compound **2** at 400 MHz (DMSO-d_6)

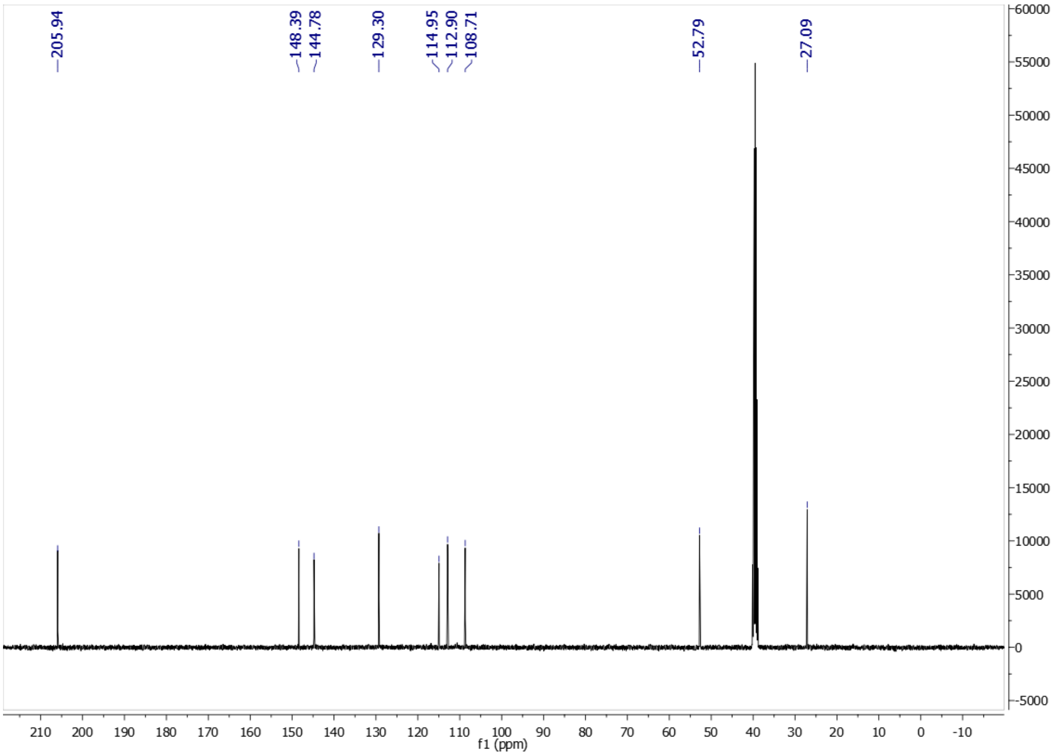

Figure S2. ^{13}C NMR of compound **2** at 101 MHz (DMSO-d_6)

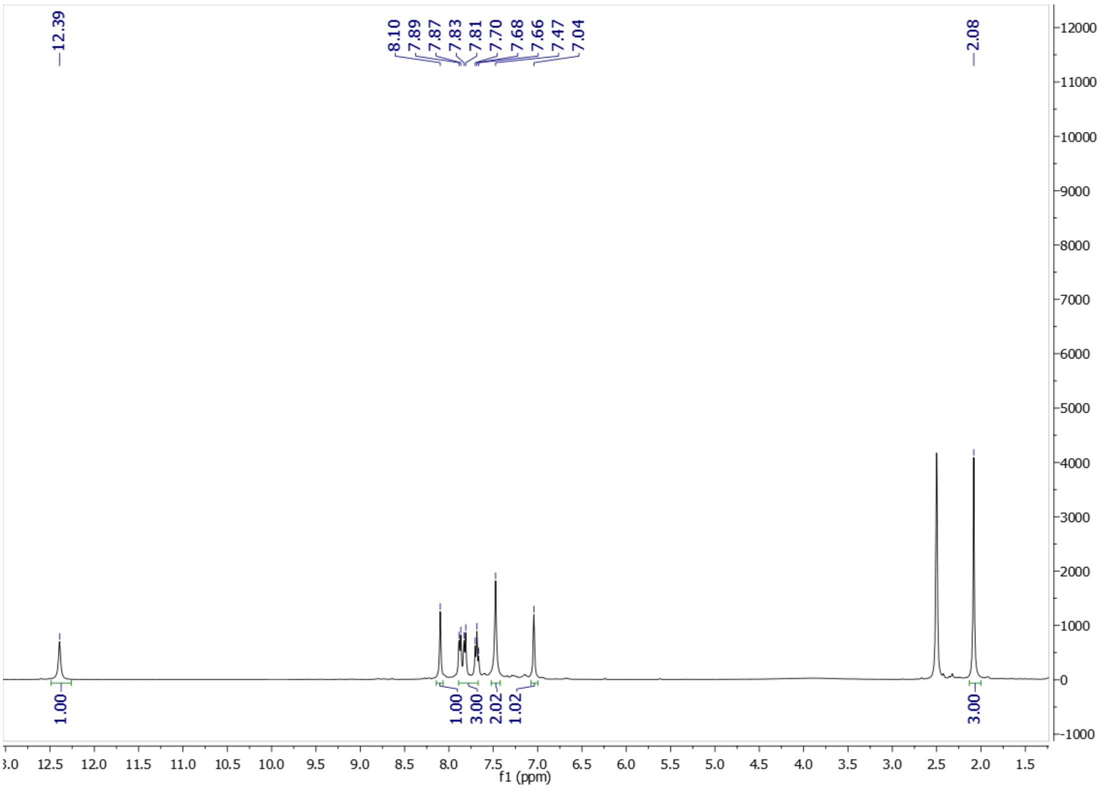

Figure S3. ^{1}H NMR of compound **3** at 400 MHz (DMSO-d_6)

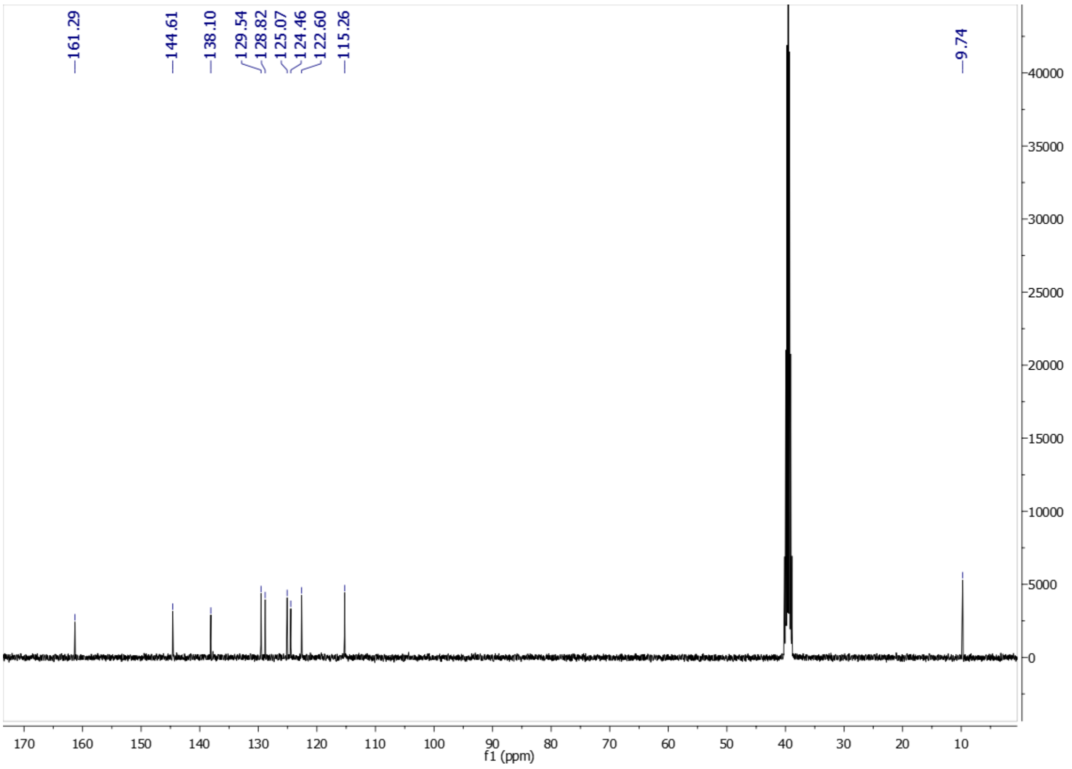

Figure S4. ^{13}C NMR of compound **3** at 101 MHz (DMSO-d_6)

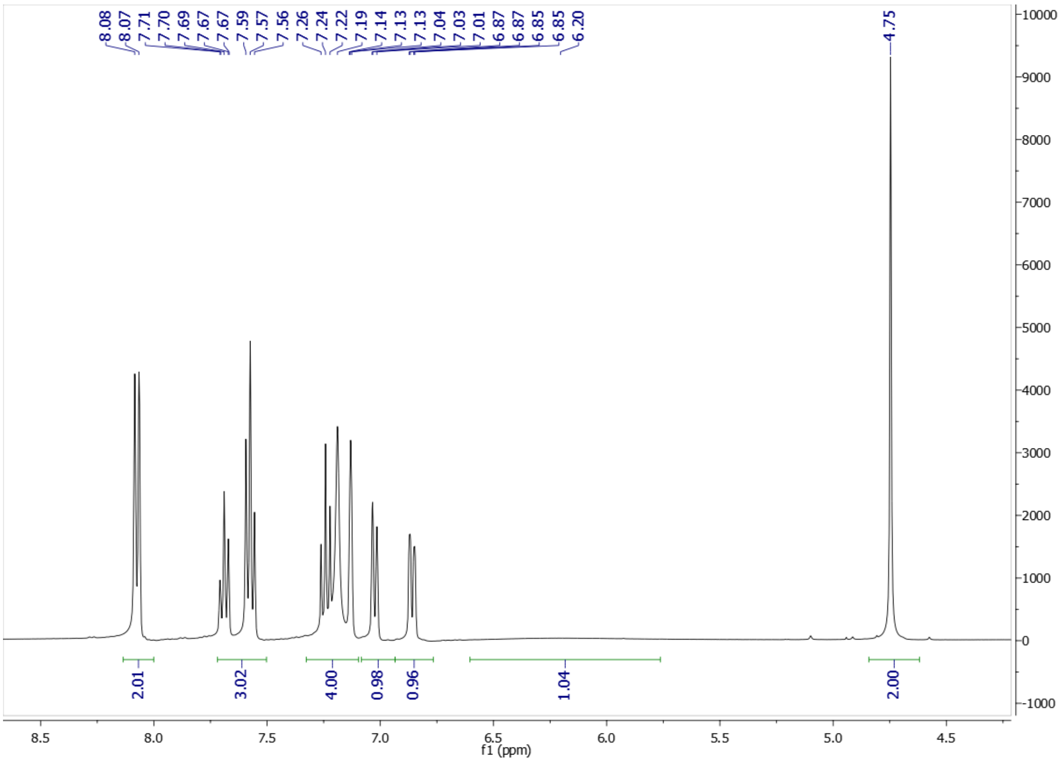

Figure S5. ^{1}H NMR of compound **4** at 400 MHz (DMSO-d_6)

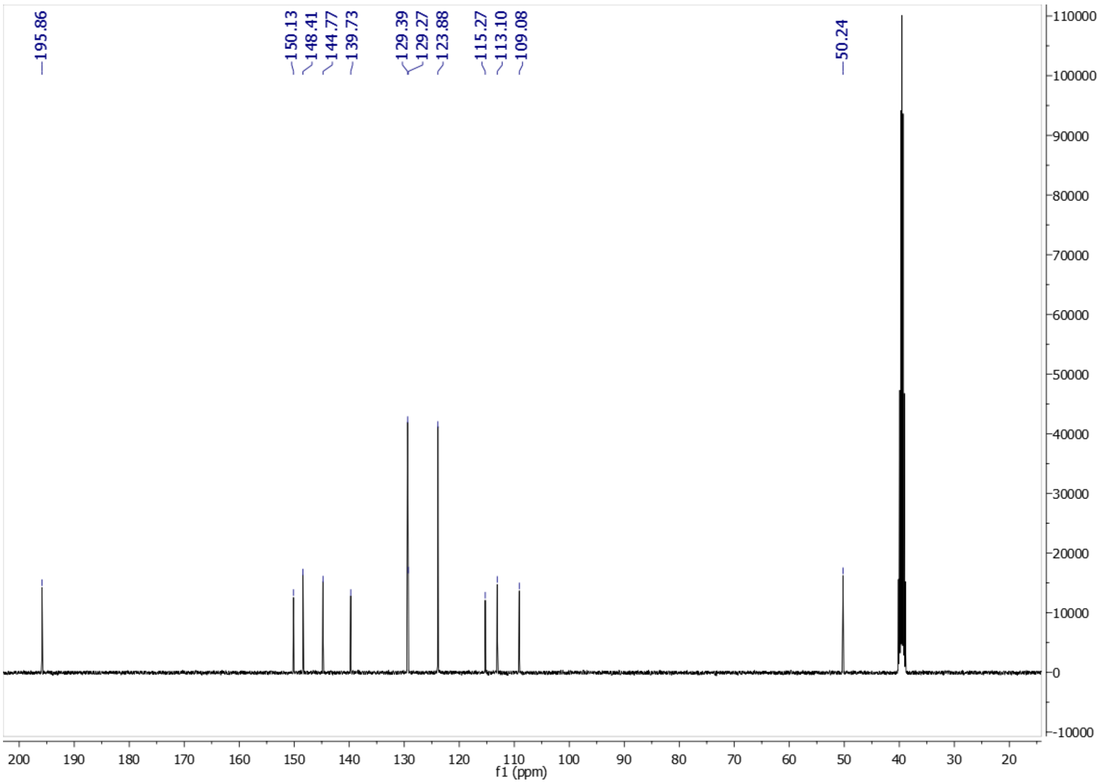

Figure S6. ^{13}C NMR of compound **4** at 101 MHz (DMSO-d_6)

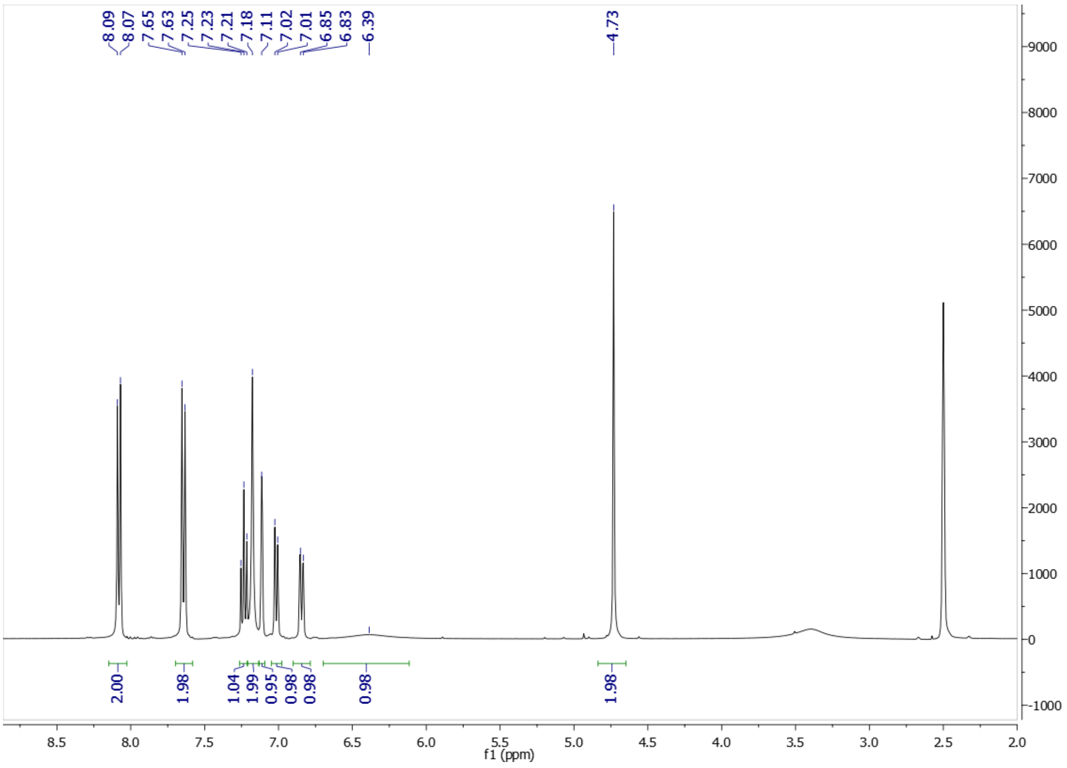

Figure S7. ^{1}H NMR of compound **5** at 400 MHz (DMSO-d_6)

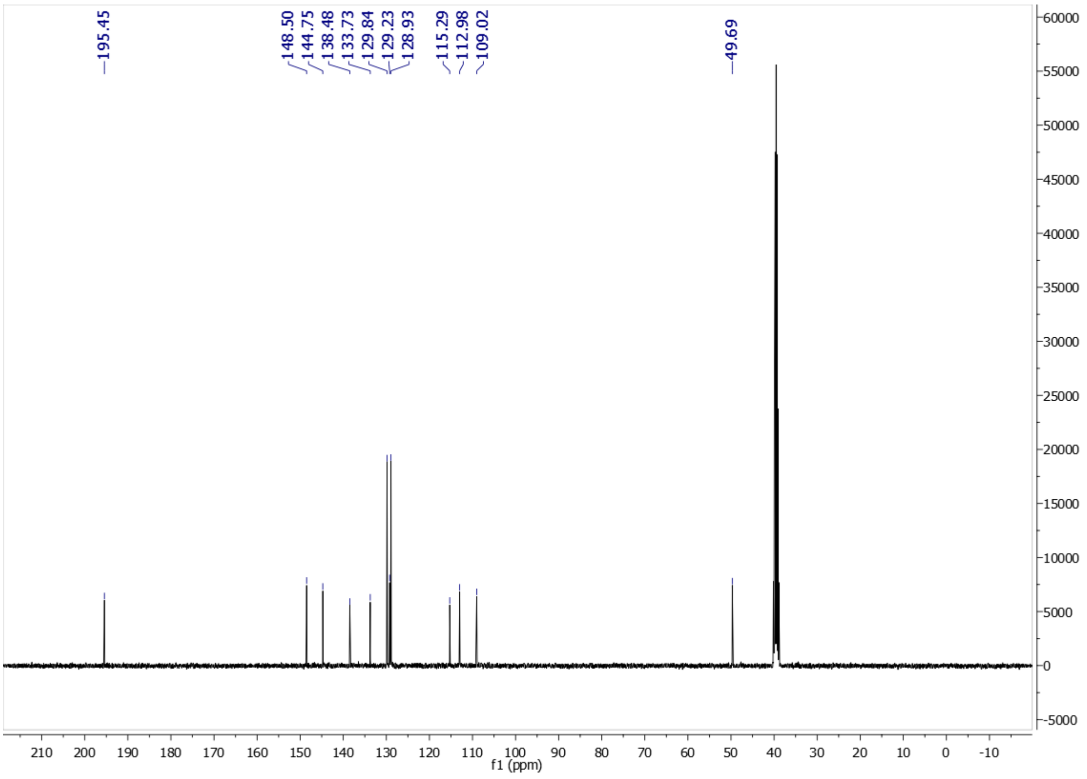

Figure S8. ^{13}C NMR of compound **5** at 101 MHz (DMSO-d_6)

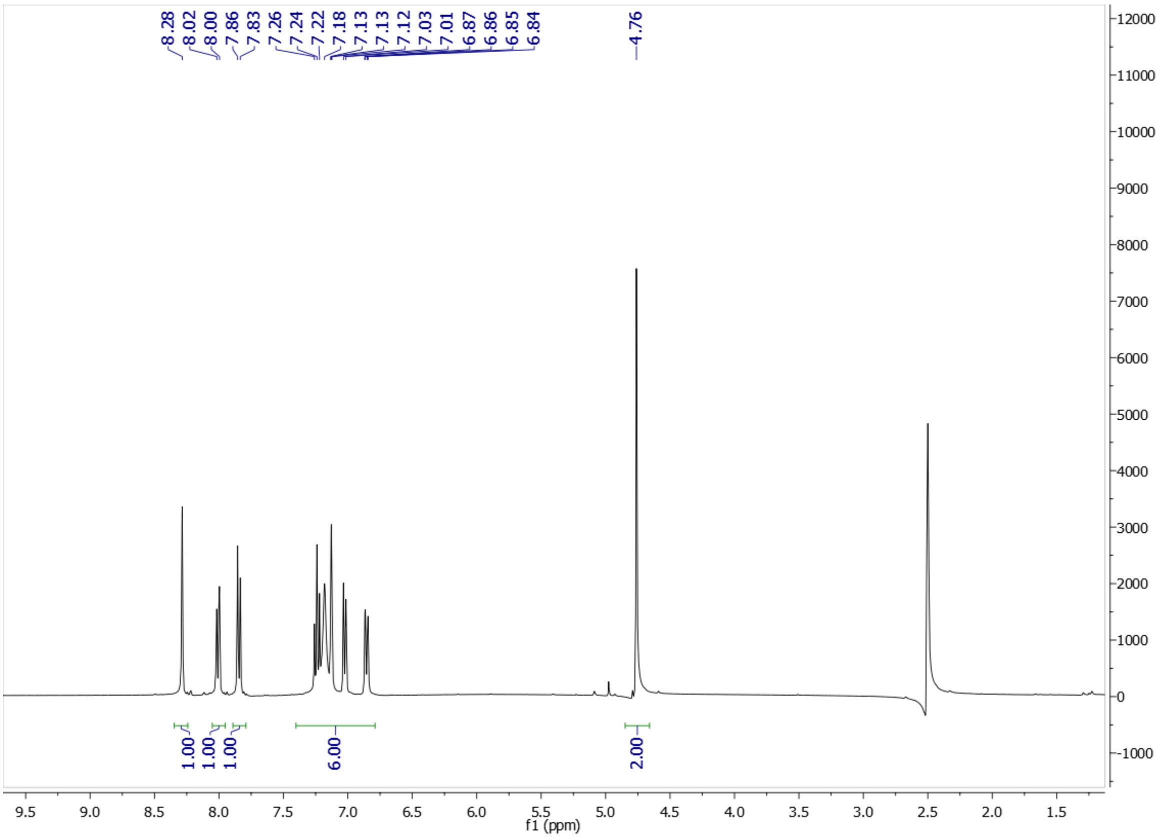

Figure S9. ^{1}H NMR of compound **6** at 400 MHz (DMSO-*d*6)

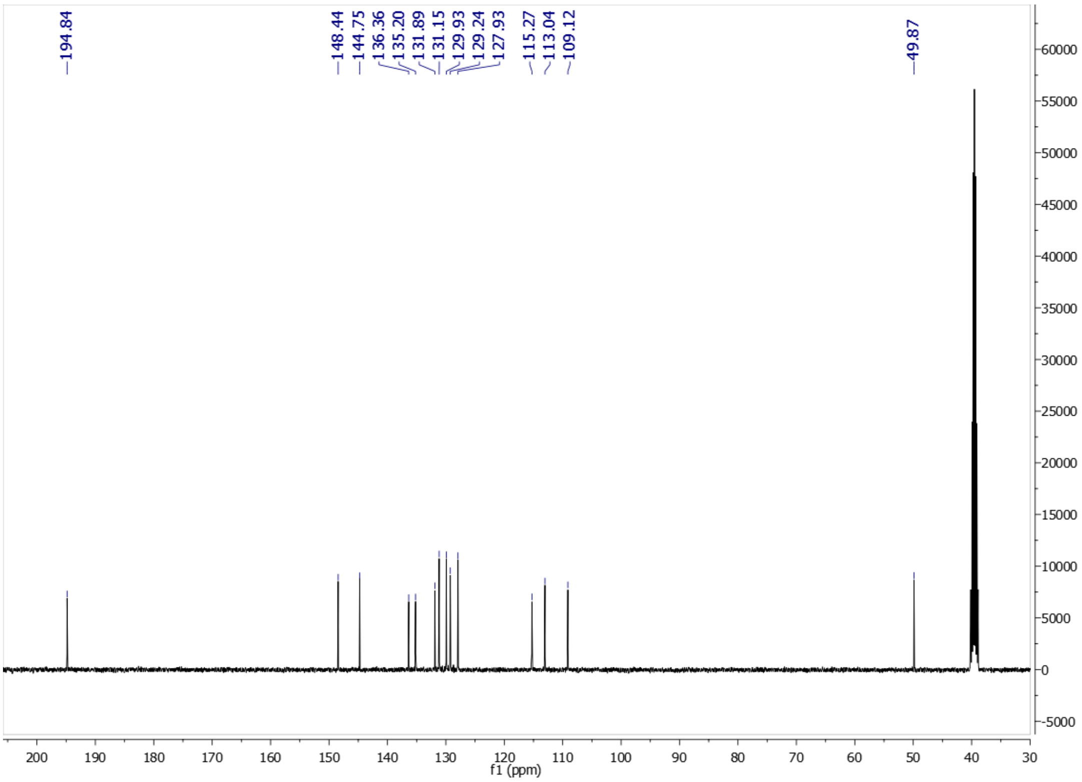

Figure S10. ^{13}C NMR of compound **6** at 101 MHz (DMSO-*d*6)

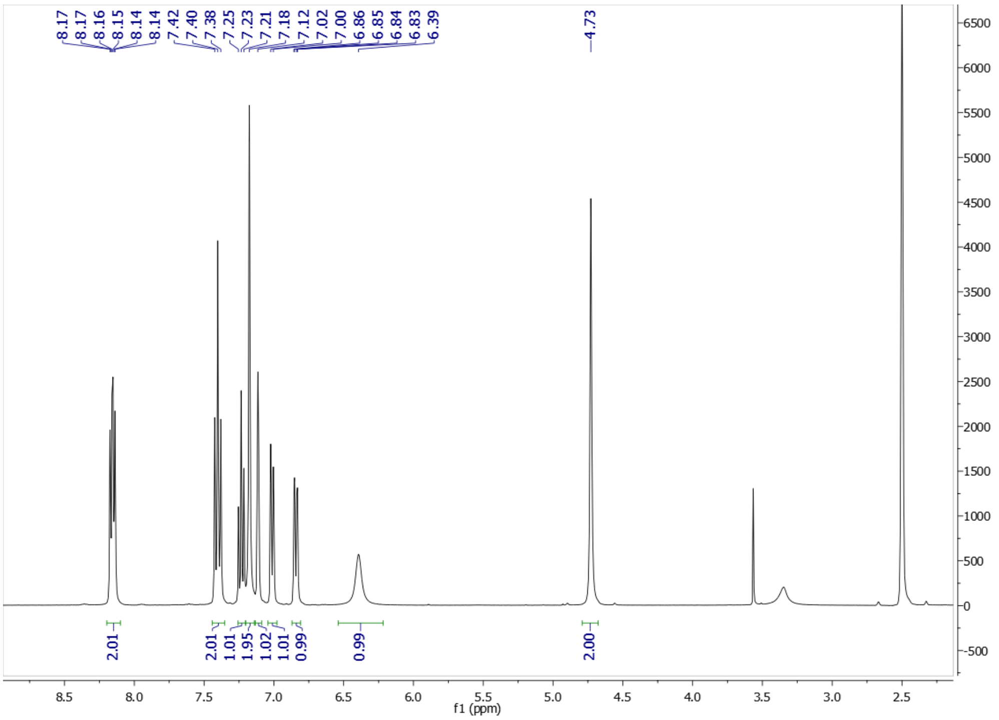

Figure S11. ^{1}H NMR of compound **7** at 400 MHz (DMSO-*d*$_6$)

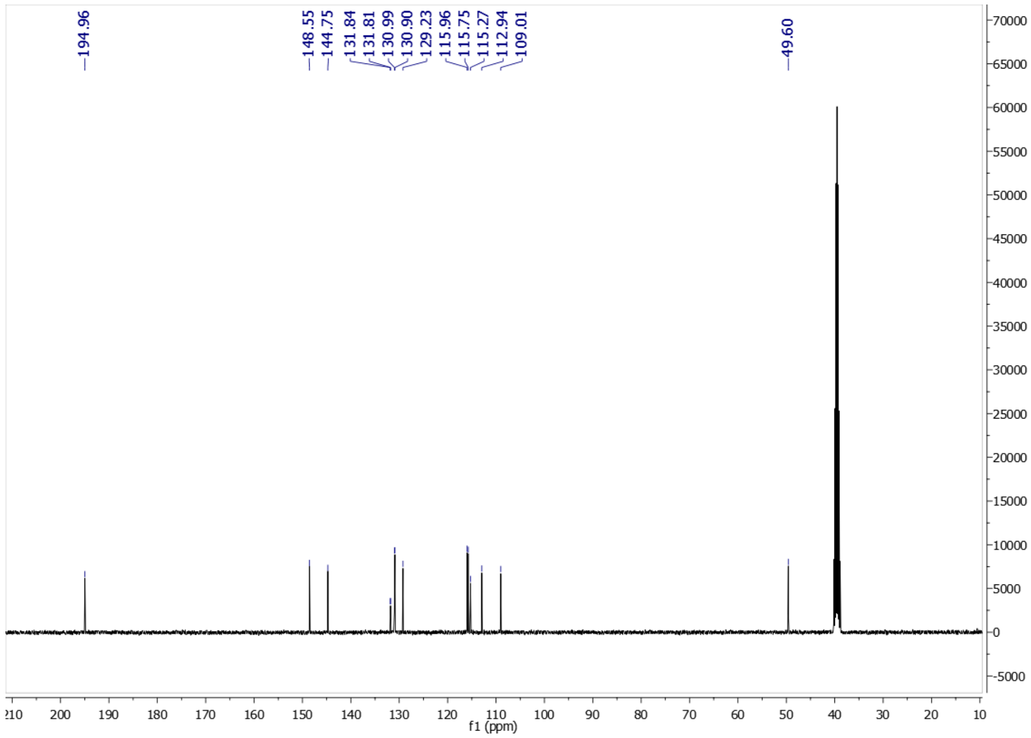

Figure S12. ^{13}C NMR of compound **7** at 101 MHz (DMSO-*d*$_6$)

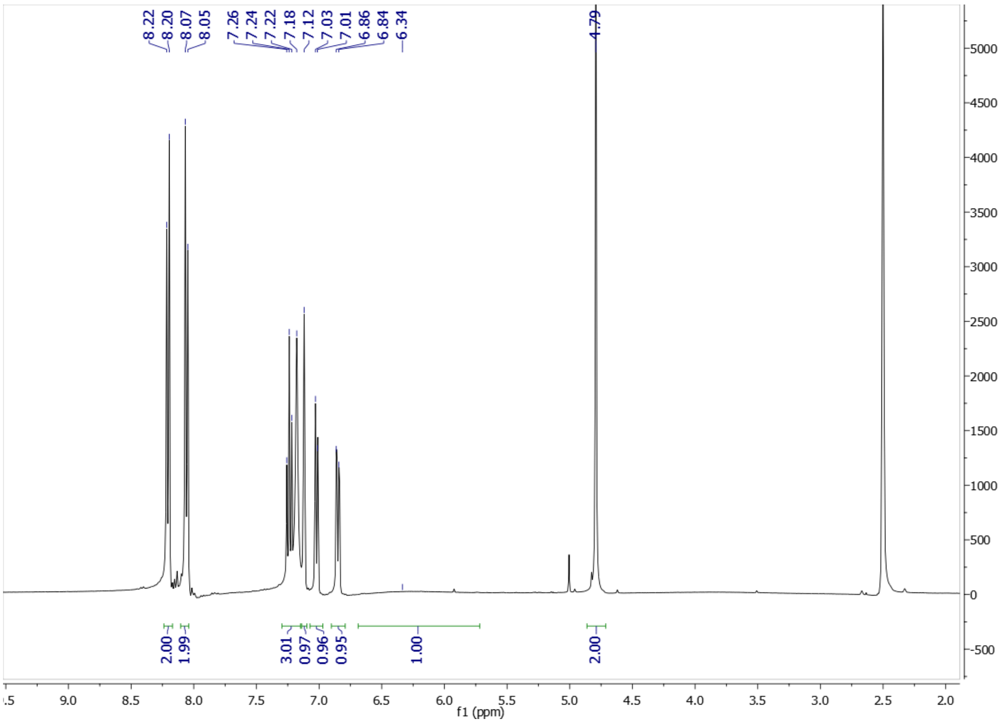

Figure S13. ^{1}H NMR of compound **8** at 400 MHz (DMSO-d_6)

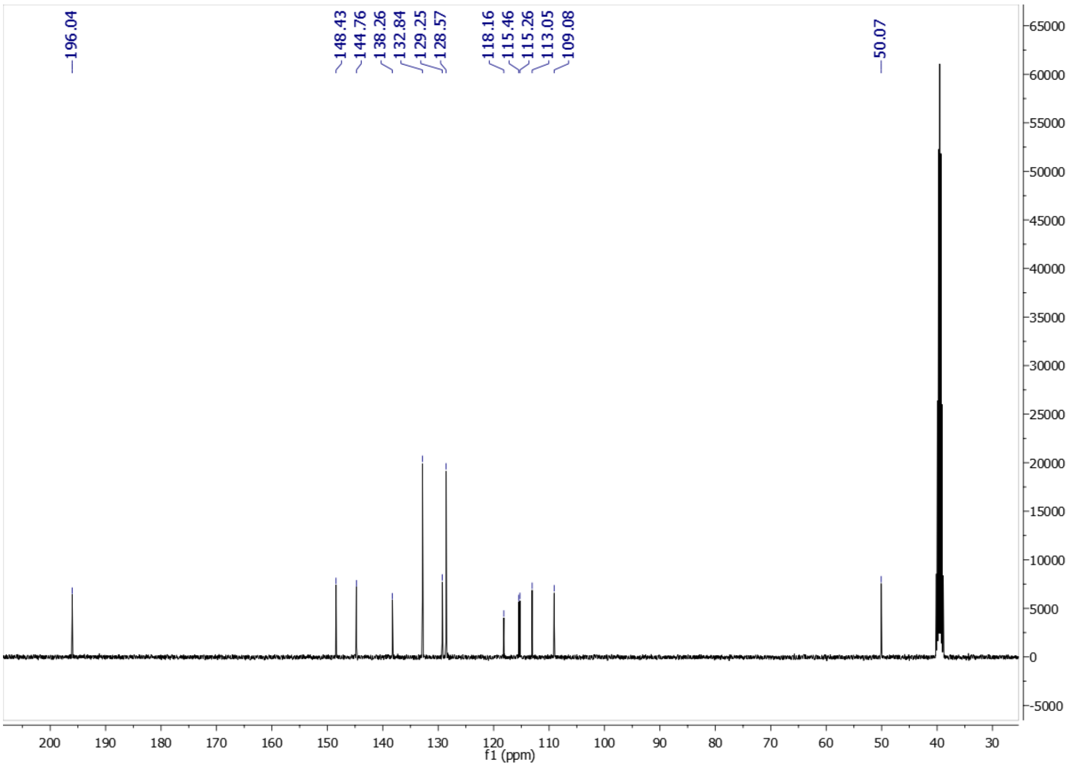

Figure S14. ^{13}C NMR of compound **8** at 101 MHz (DMSO-d_6)

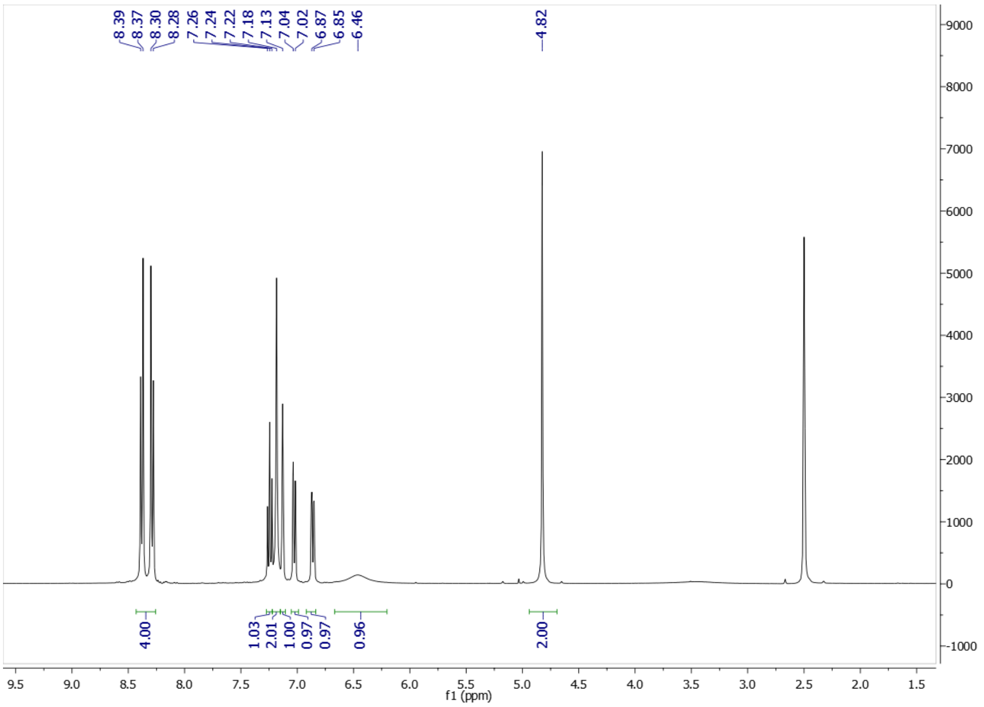

Figure S15. ^{1}H NMR of compound **9** at 400 MHz (DMSO-d_6)

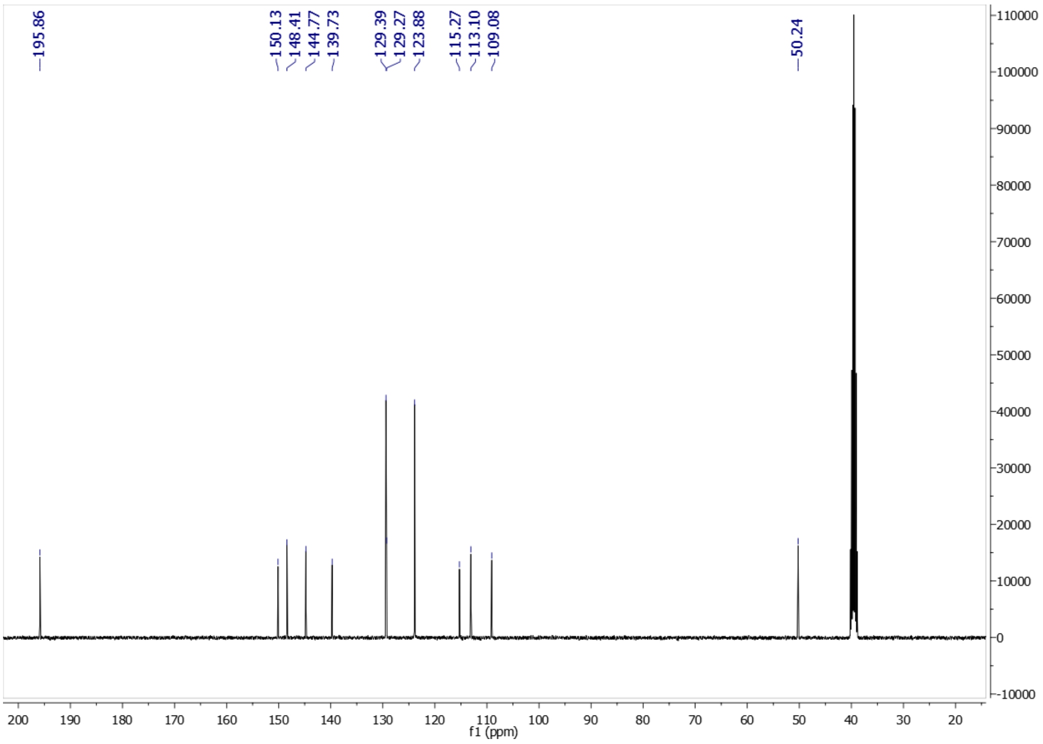

Figure S16. ^{13}C NMR of compound **9** at 101 MHz (DMSO-d_6)

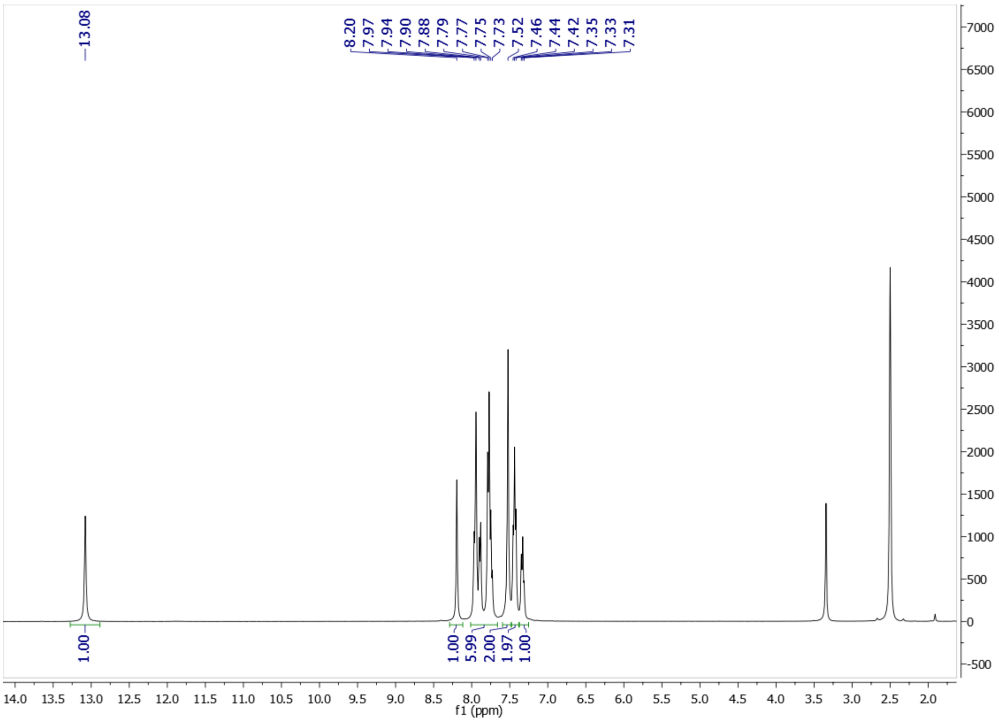

Figure S17. ^{1}H NMR of compound **10** at 400 MHz (DMSO-d_6)

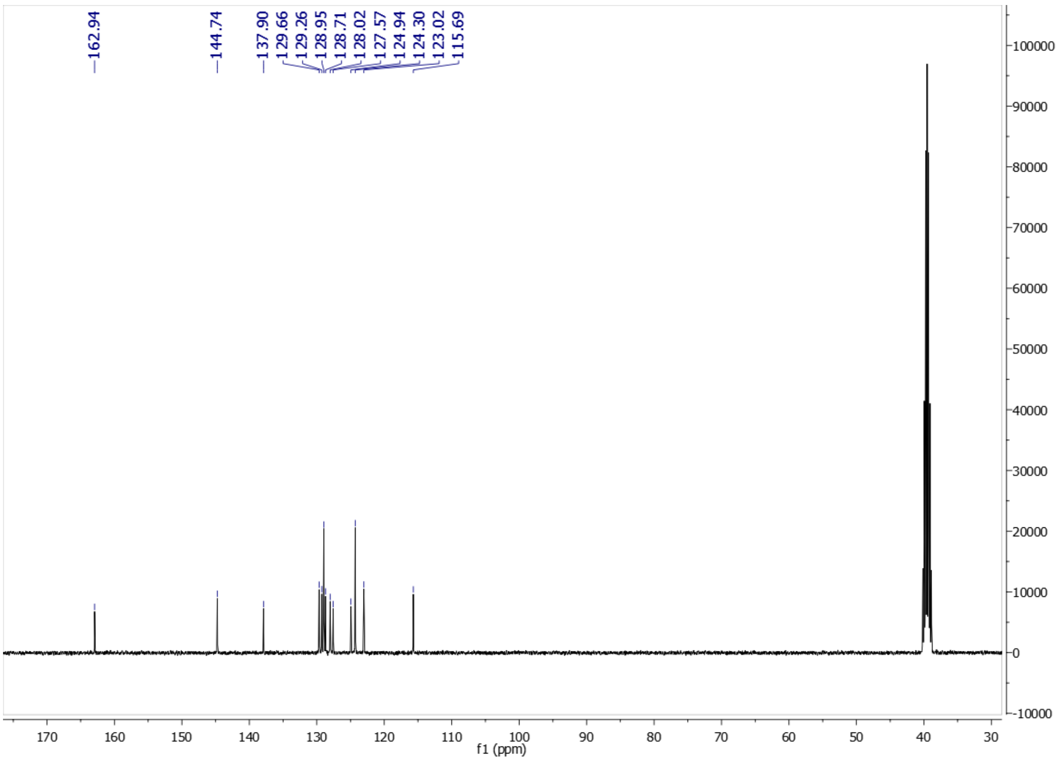

Figure S18. ^{13}C NMR of compound **10** at 101 MHz (DMSO-d_6)

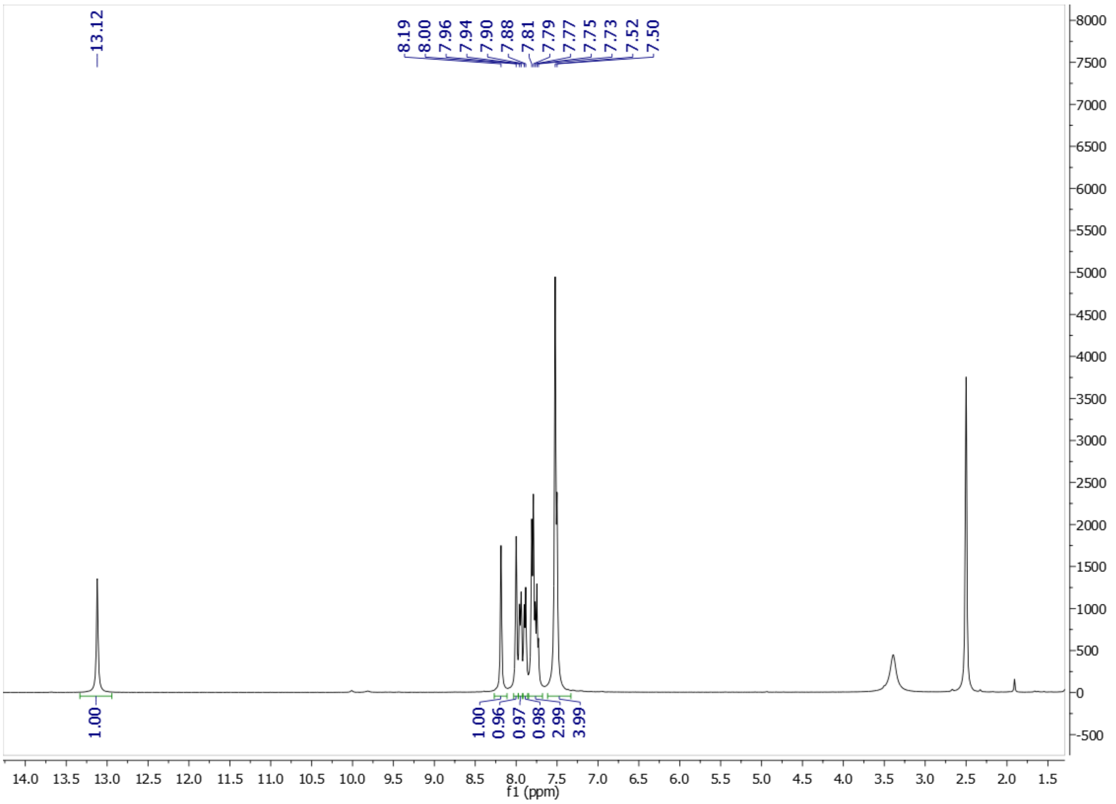

Figure S19. ¹H NMR of compound **11** at 400 MHz (DMSO-*d*₆)

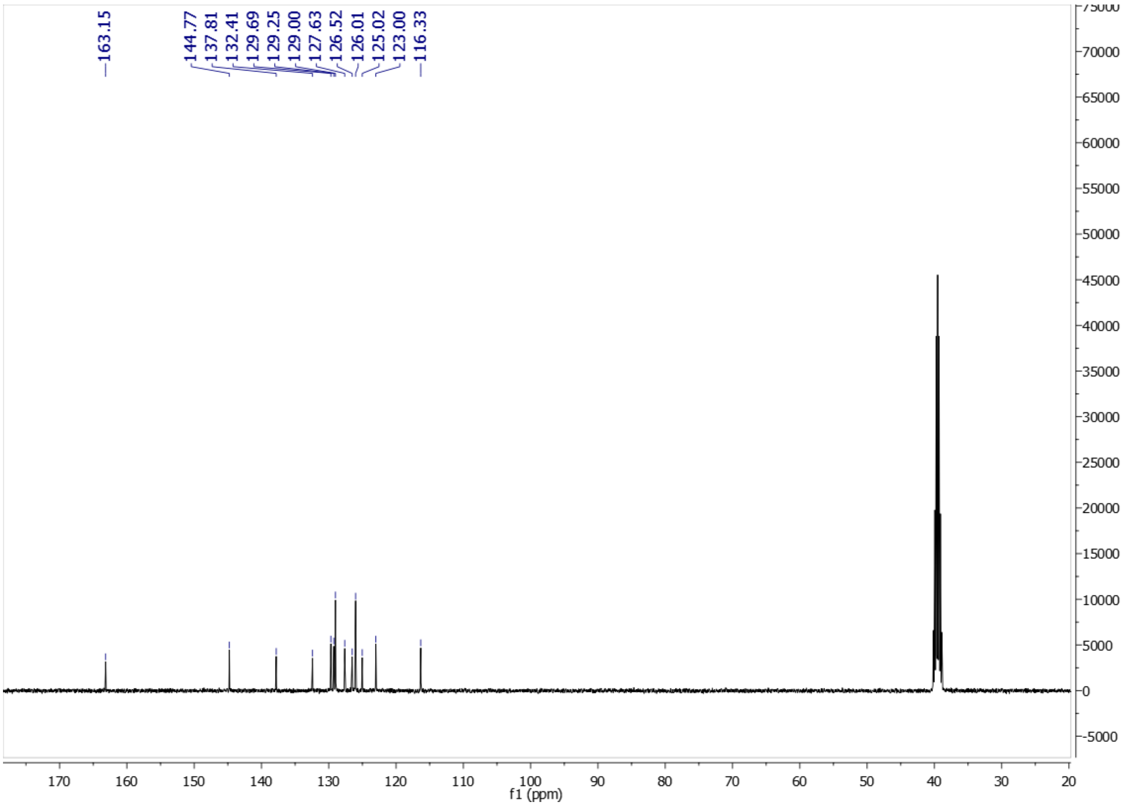

Figure S20. ¹³C NMR of compound **11** at 101 MHz (DMSO-*d*₆)

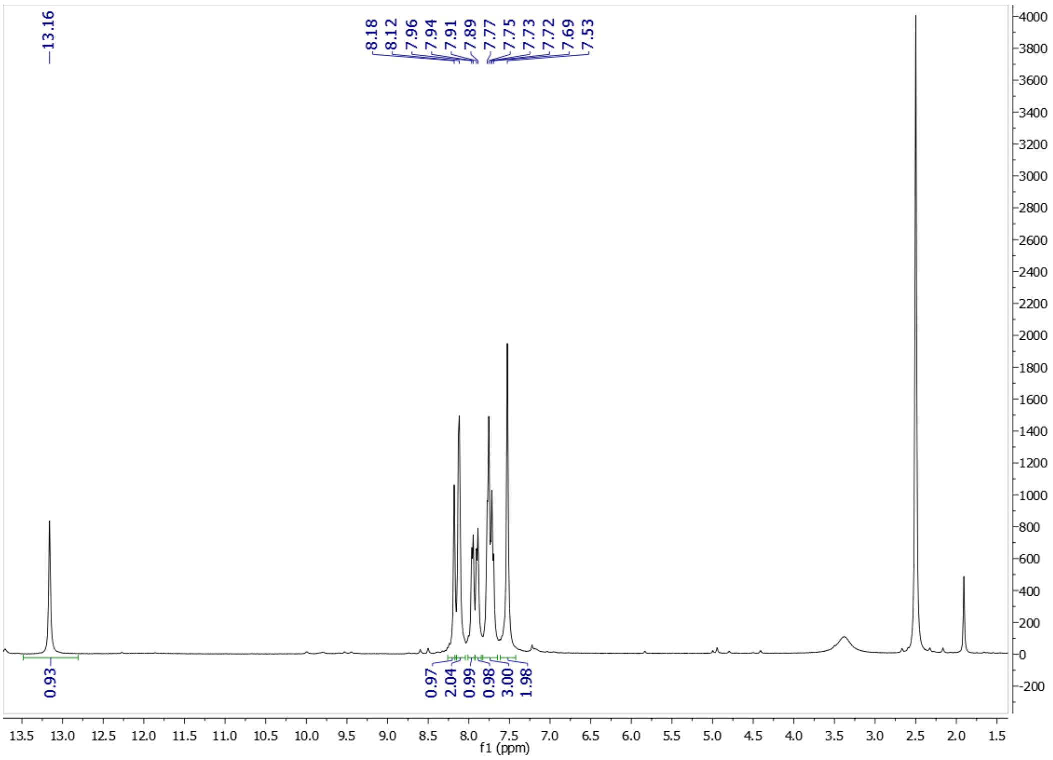

Figure S21. ^{1}H NMR of compound **12** at 400 MHz (DMSO-d_6)

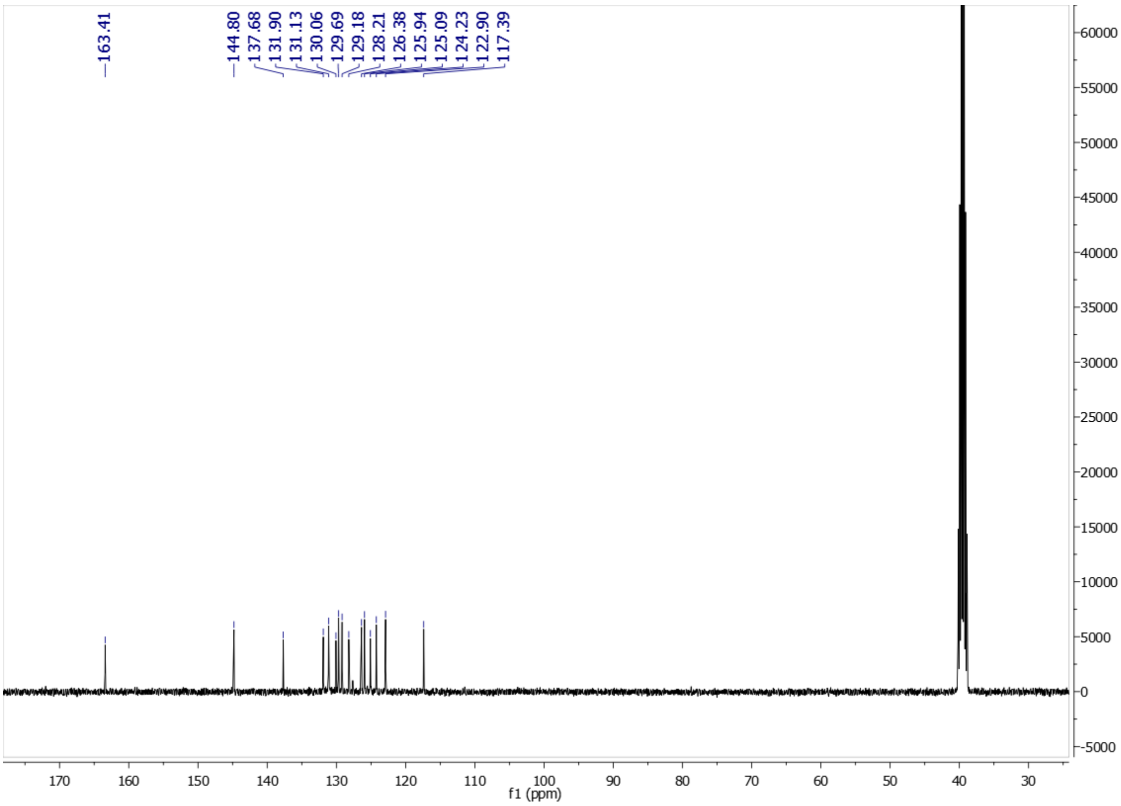

Figure S22. ^{13}C NMR of compound **12** at 101 MHz (DMSO-d_6)

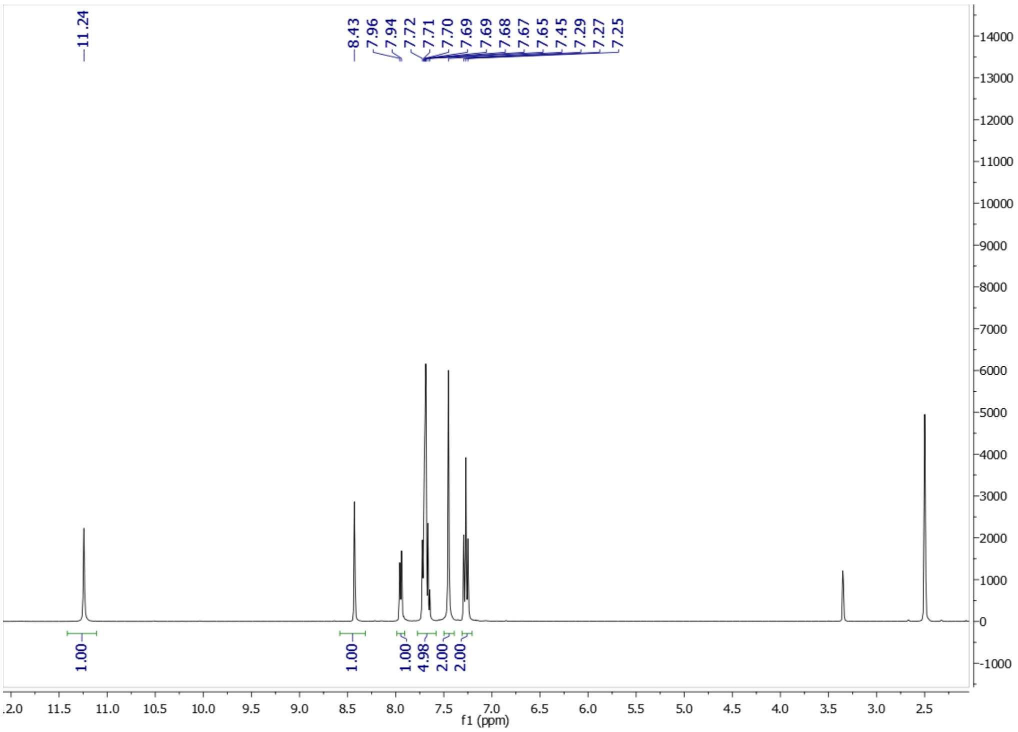

Figure S23. ^{1}H NMR of compound **13** at 400 MHz (DMSO-d_6)

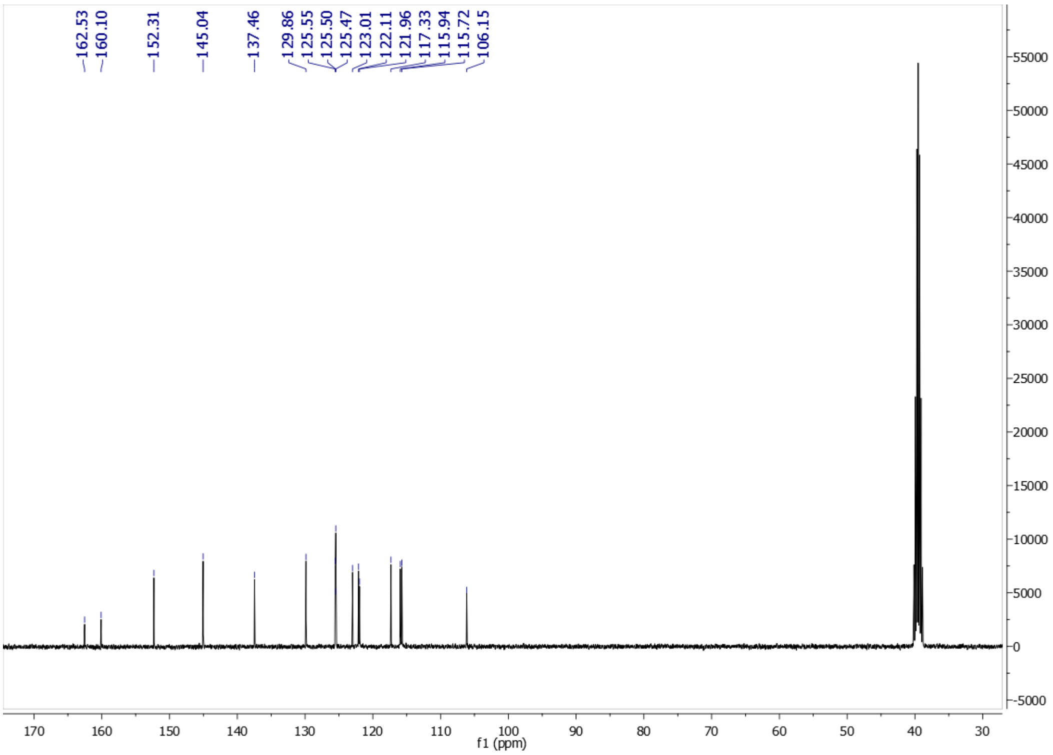

Figure S24. ^{13}C NMR of compound **13** at 101 MHz (DMSO-d_6)

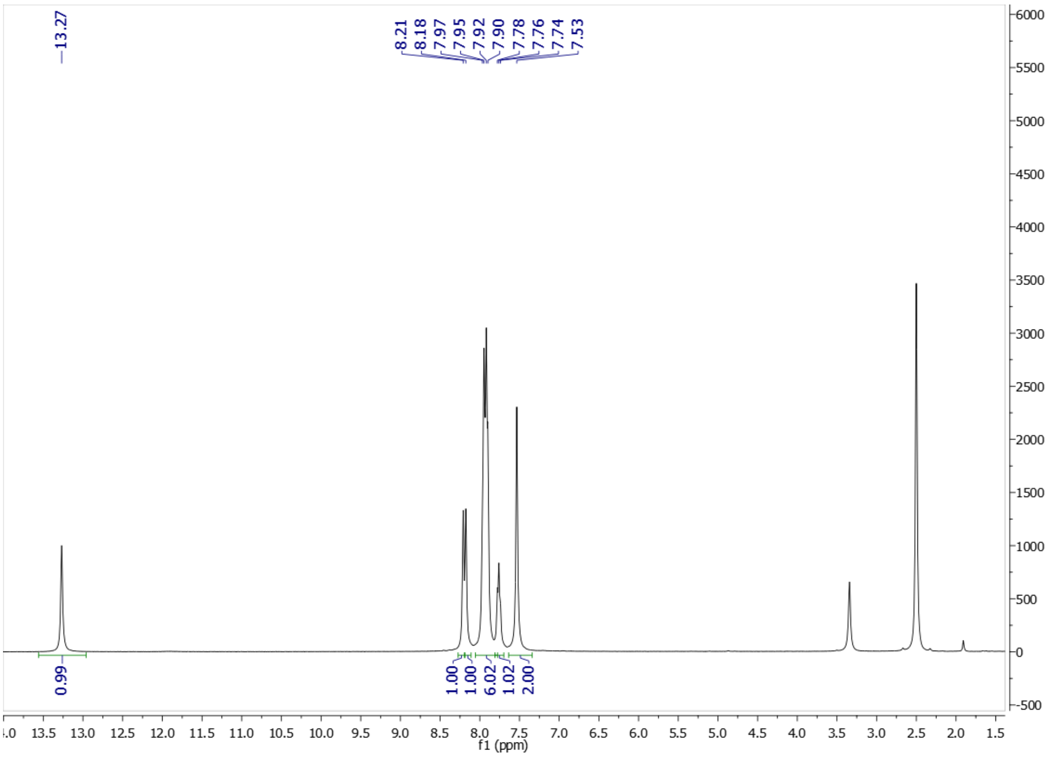

Figure S25. ^{1}H NMR of compound **14** at 400 MHz (DMSO-d_6)

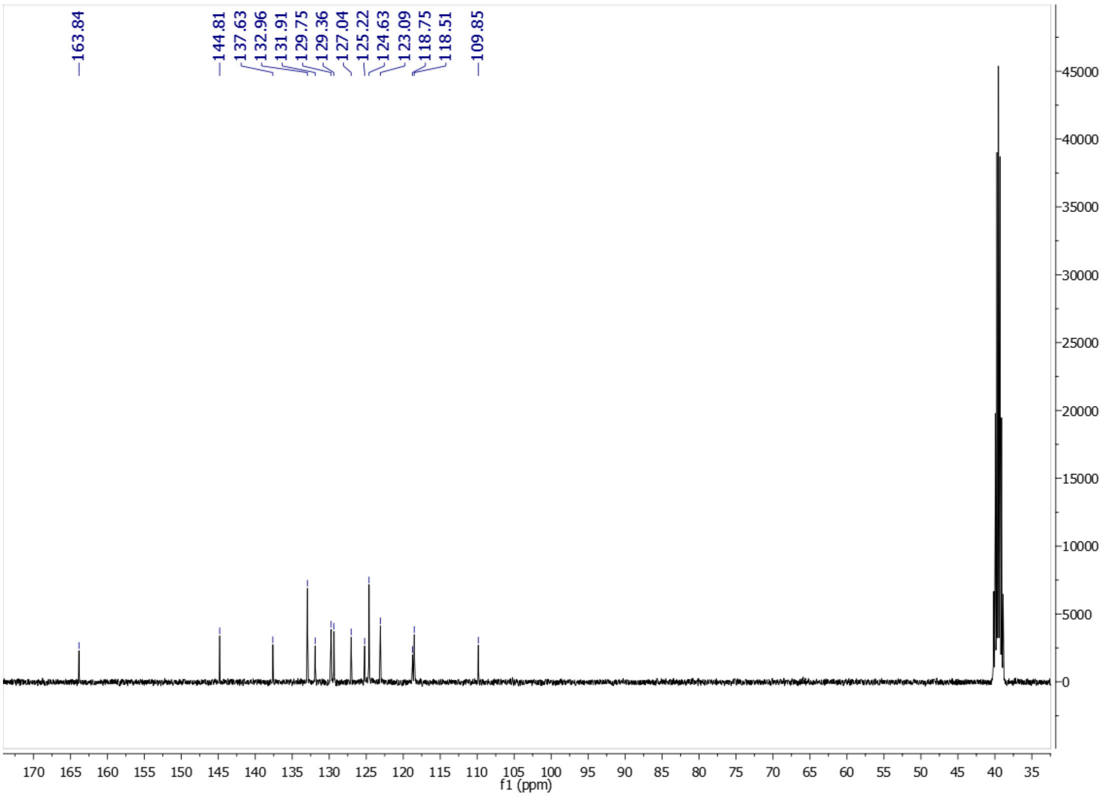

Figure S26. ^{13}C NMR of compound **14** at 101 MHz (DMSO-d_6)

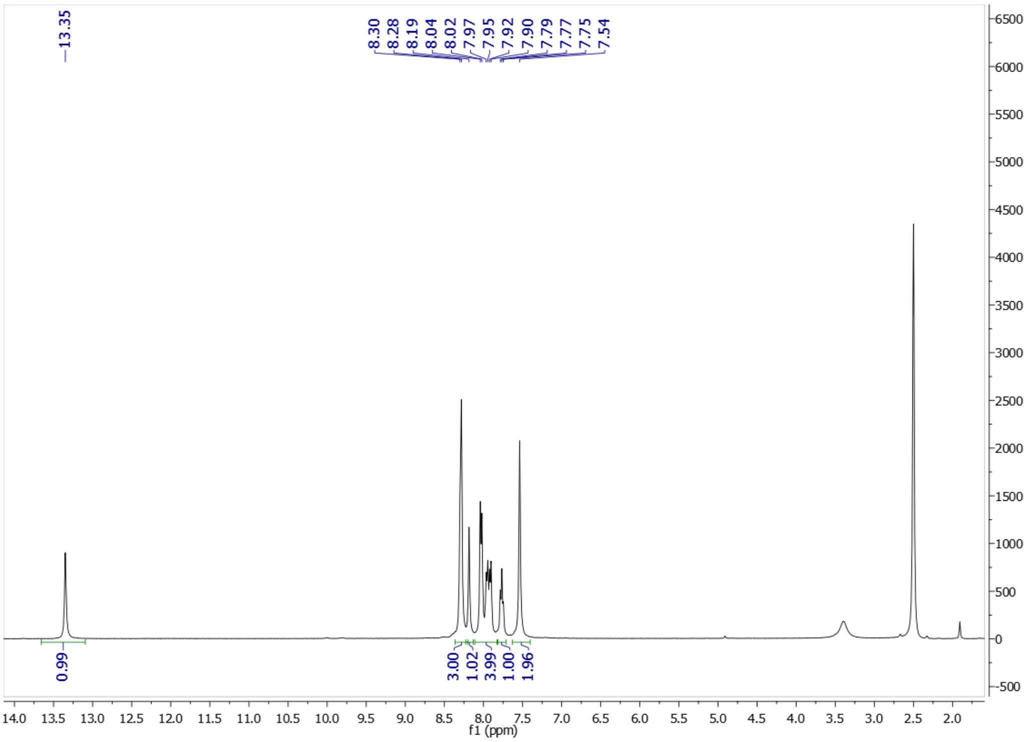

Figure S27. ^{1}H NMR of compound **15** at 400 MHz (DMSO-d_6)

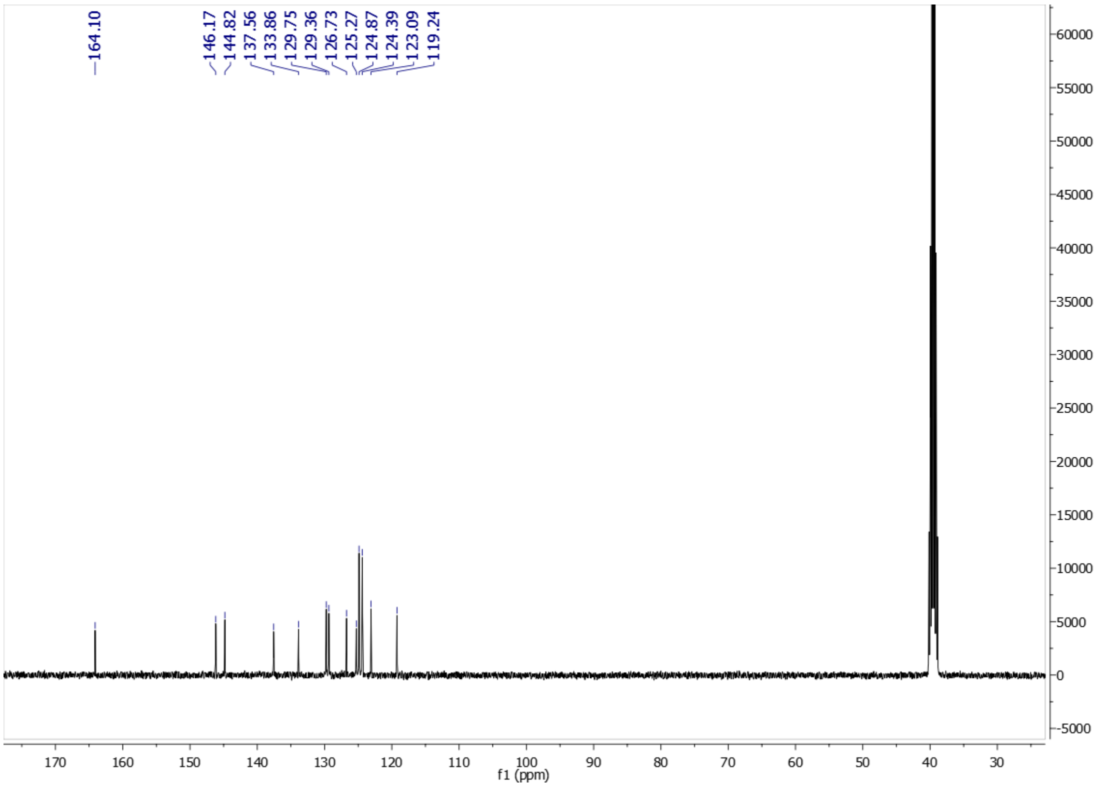

Figure S28. ^{13}C NMR of compound **15** at 101 MHz (DMSO-d_6)

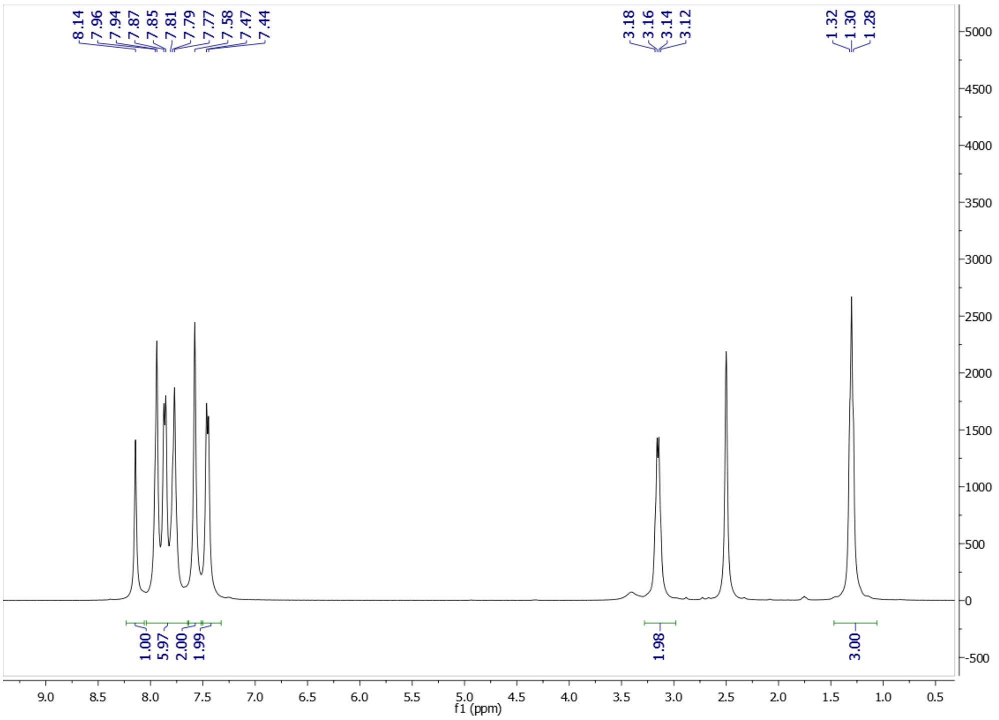

Figure S29. ^{1}H NMR of compound **16** at 400 MHz (DMSO-d_6)

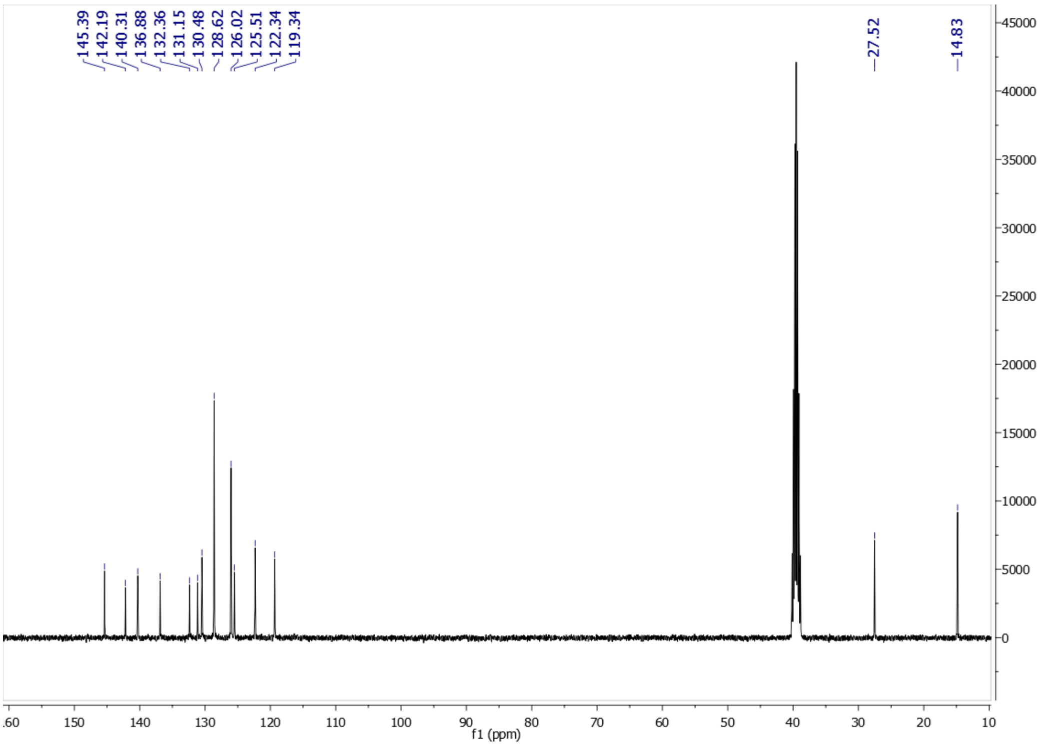

Figure S30. ^{13}C NMR of compound **16** at 101 MHz (DMSO-d_6)

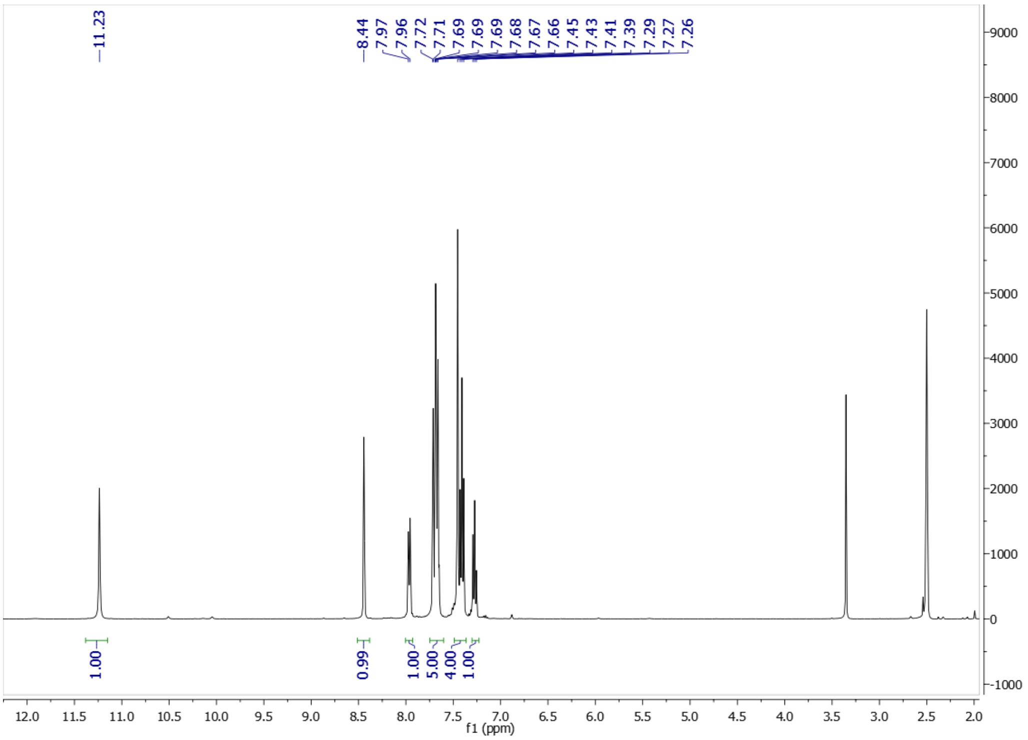

Figure S31. ^{1}H NMR of compound **17** at 400 MHz (DMSO-d_6)

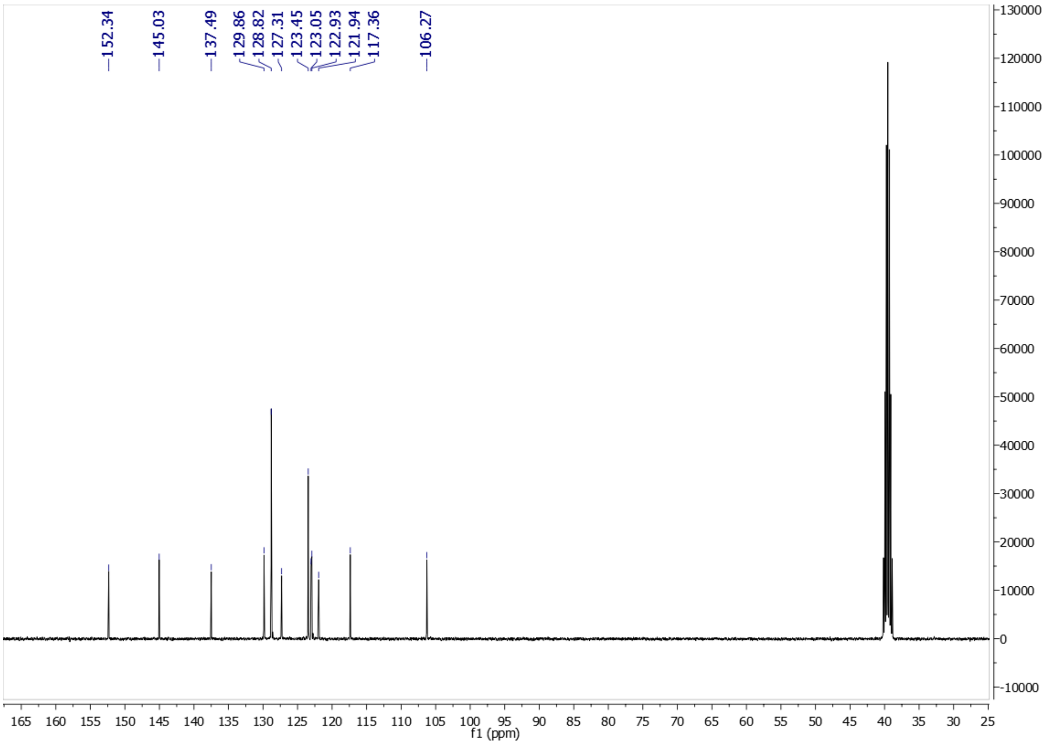

Figure S32. ^{13}C NMR of compound **17** at 101 MHz (DMSO-d_6)

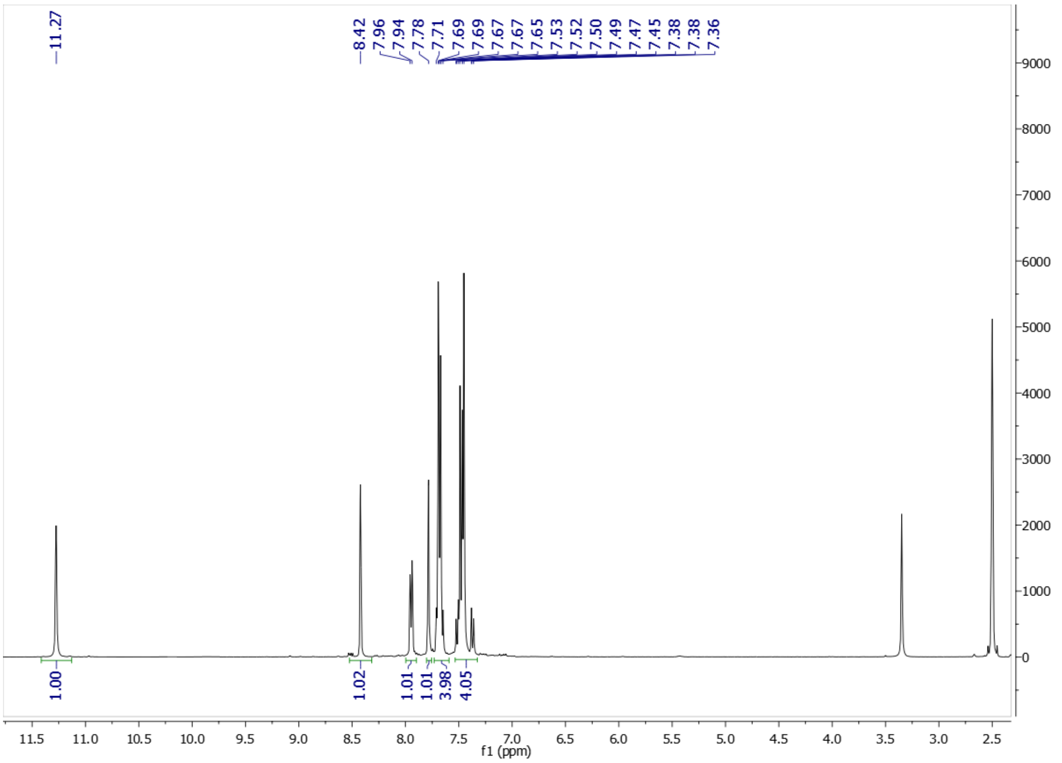

Figure S33. ^{1}H NMR of compound **18** at 400 MHz (DMSO-d_6)

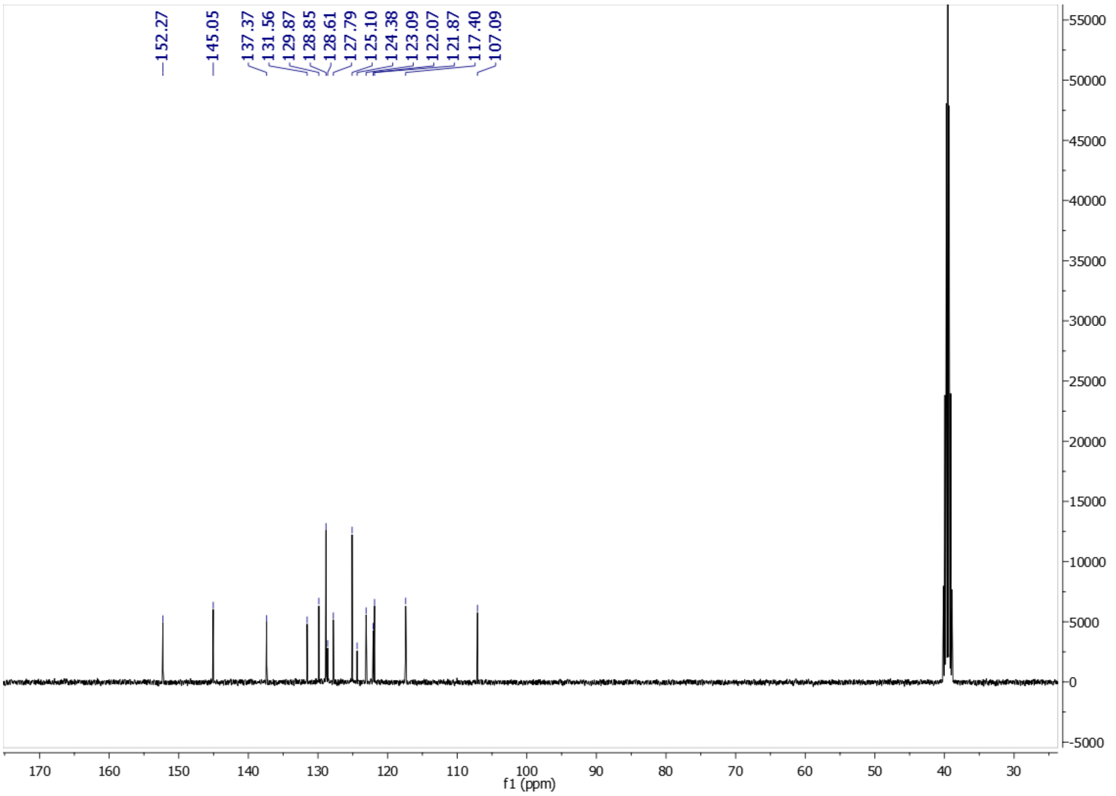

Figure S34. ^{13}C NMR of compound **18** at 101 MHz (DMSO-d_6)

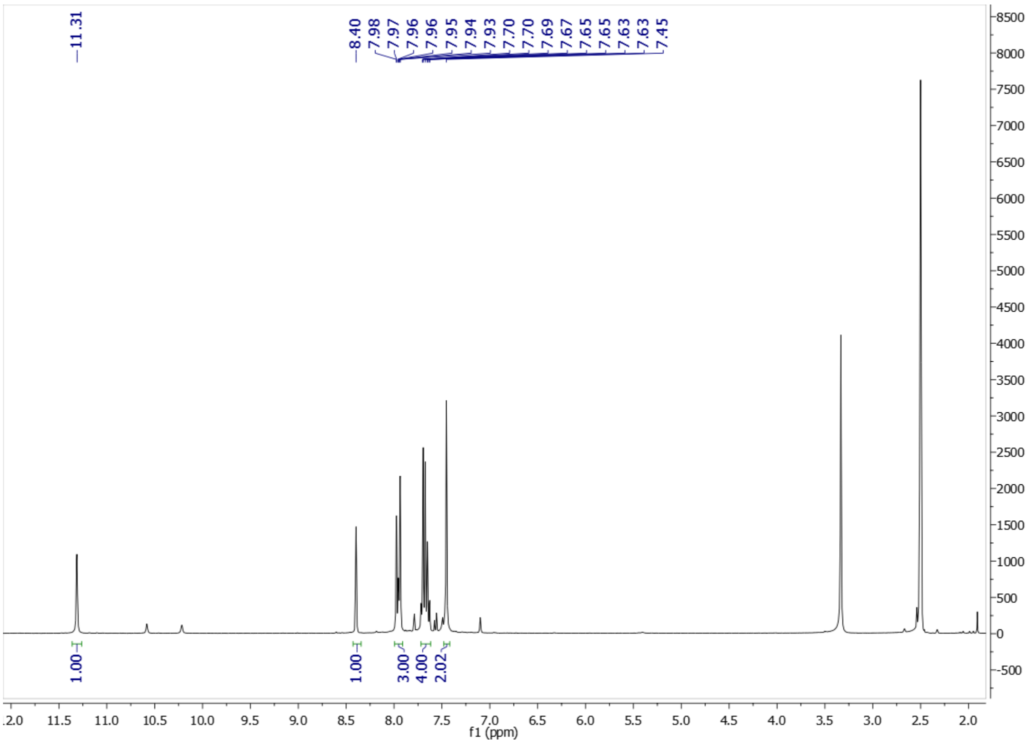

Figure S35. ^{1}H NMR of compound **19** at 400 MHz (DMSO-d_6)

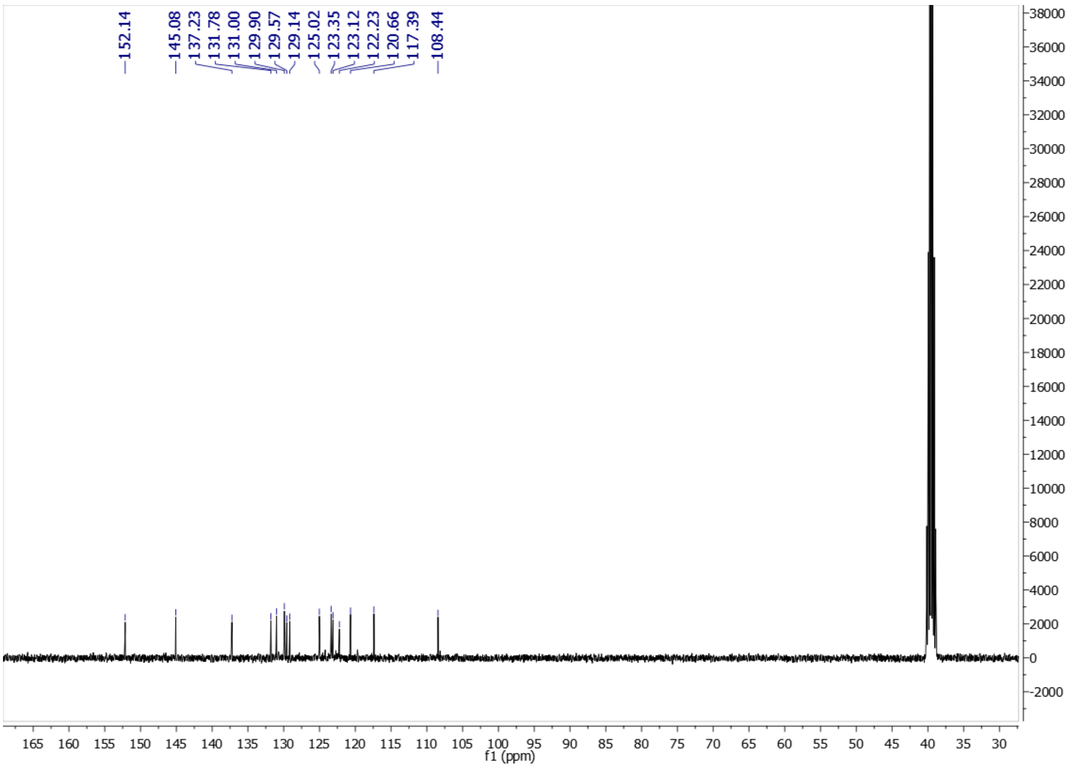

Figure S36. ^{13}C NMR of compound **19** at 101 MHz (DMSO-d_6)

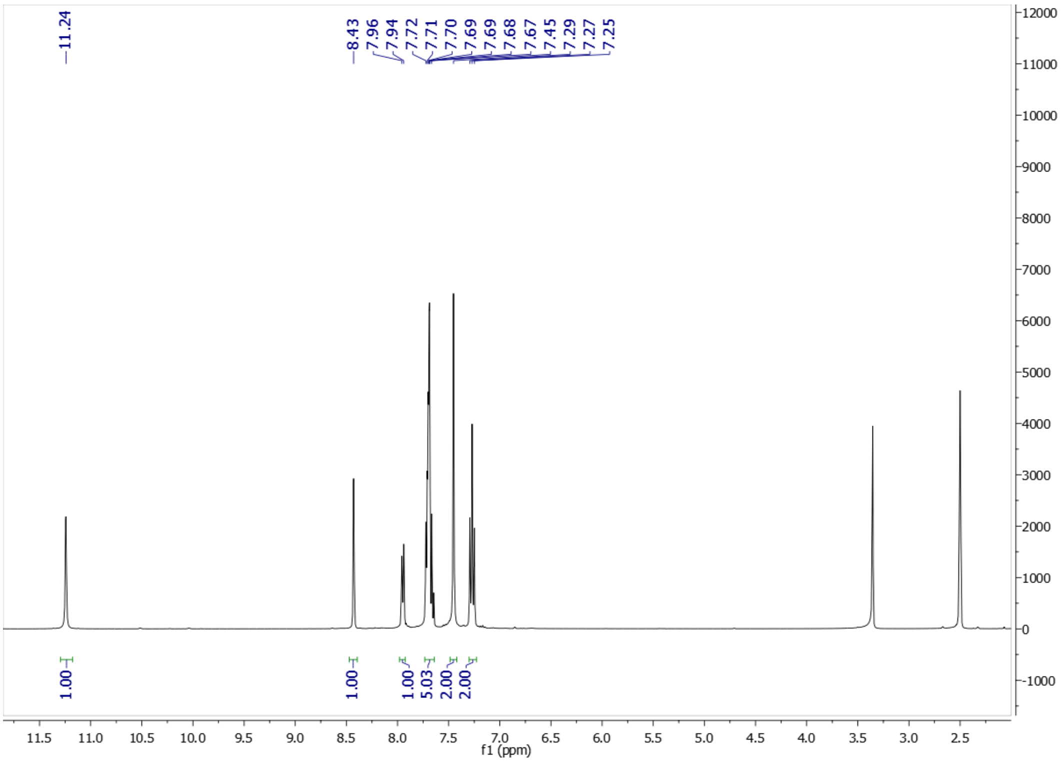

Figure S37. ^{1}H NMR of compound **20** at 400 MHz (DMSO-d_6)

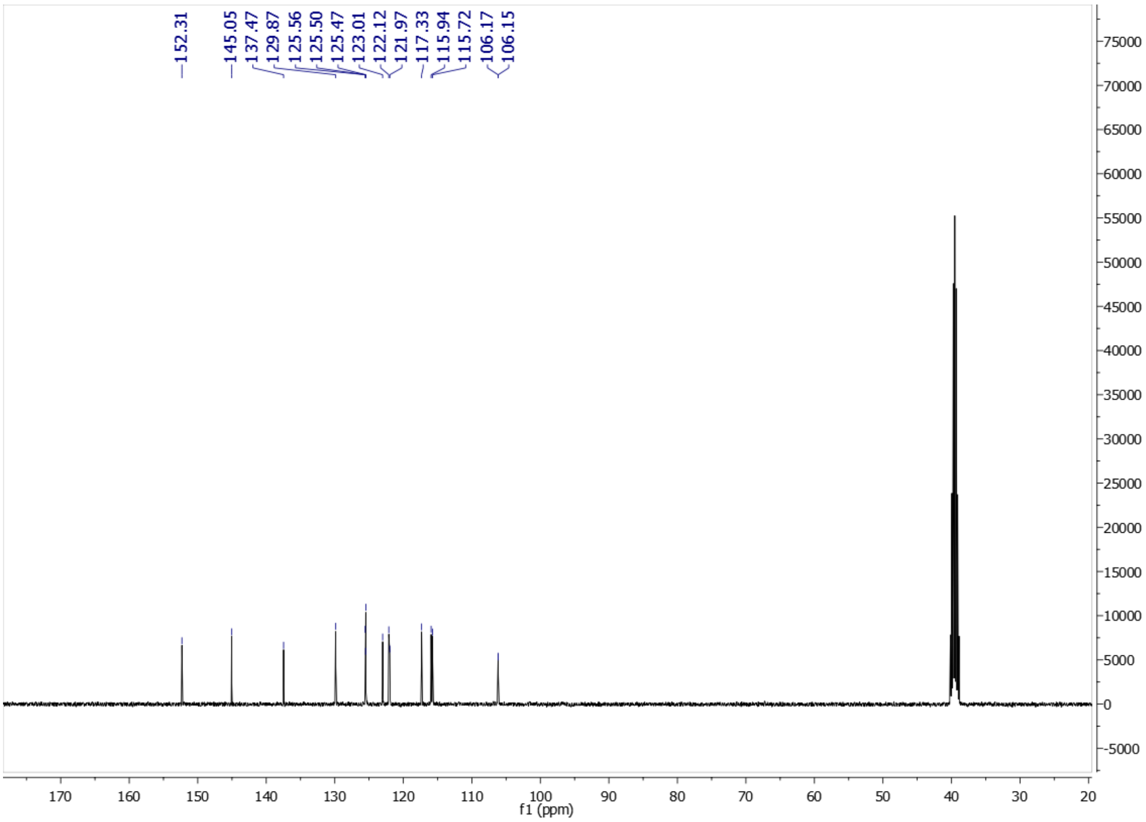

Figure S38. ^{13}C NMR of compound **20** at 101 MHz (DMSO-d_6)

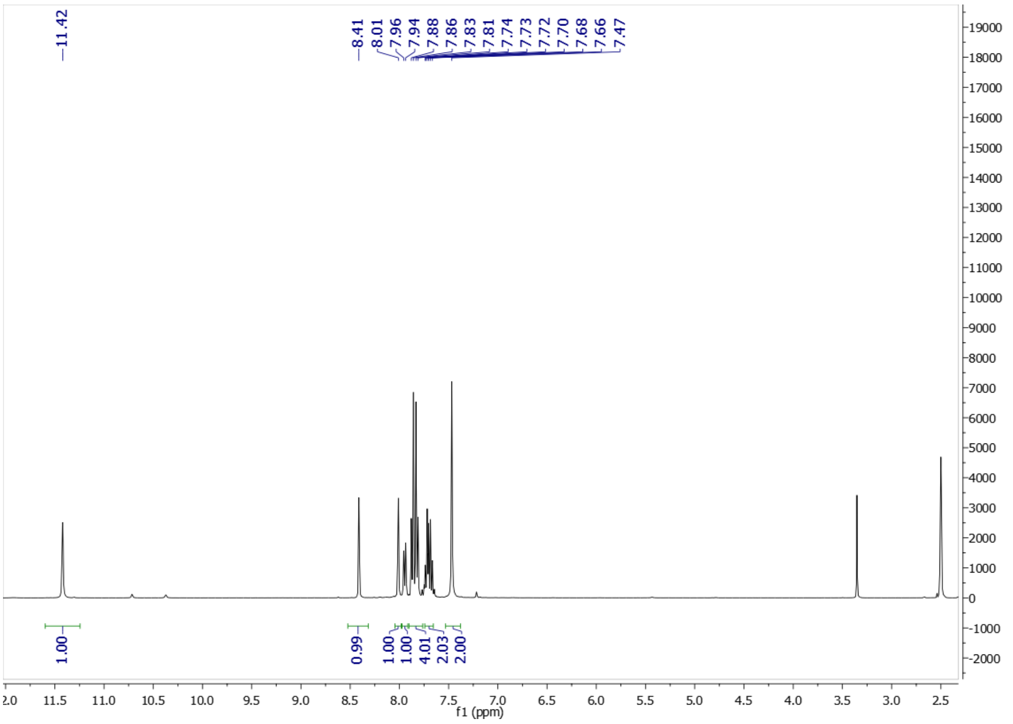

Figure S39. ^{1}H NMR of compound **21** at 400 MHz (DMSO-*d*$_6$)

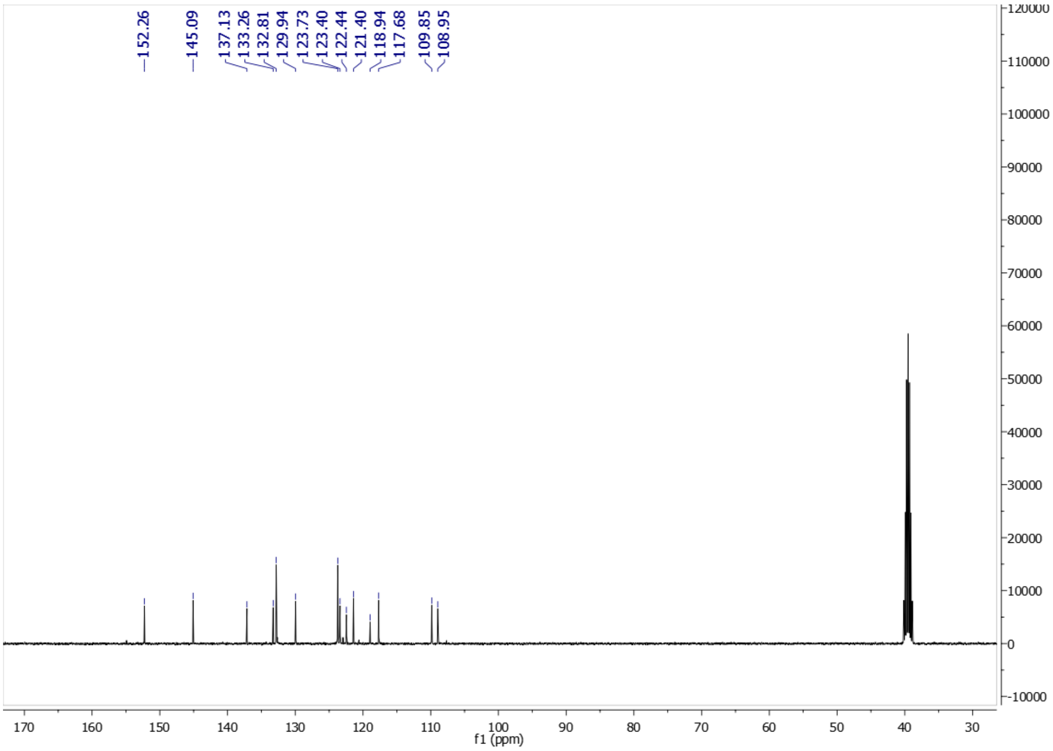

Figure S40. ^{13}C NMR of compound **21** at 101 MHz (DMSO-*d*$_6$)

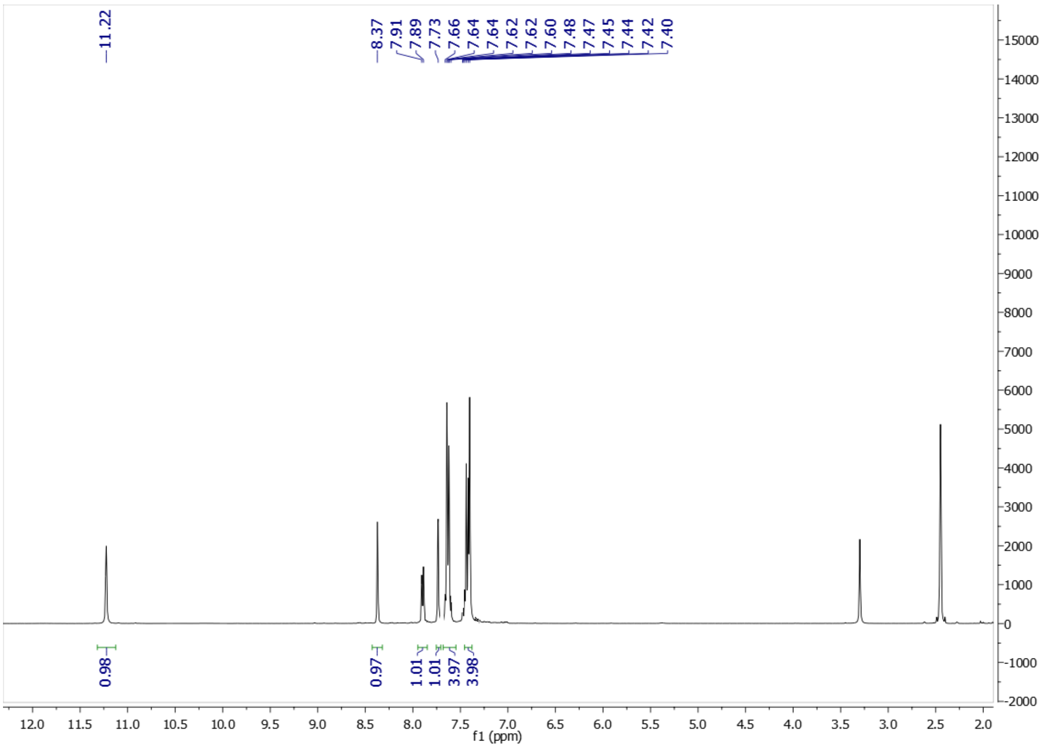

Figure S41. ^{1}H NMR of compound **22** at 400 MHz (DMSO-*d*6)

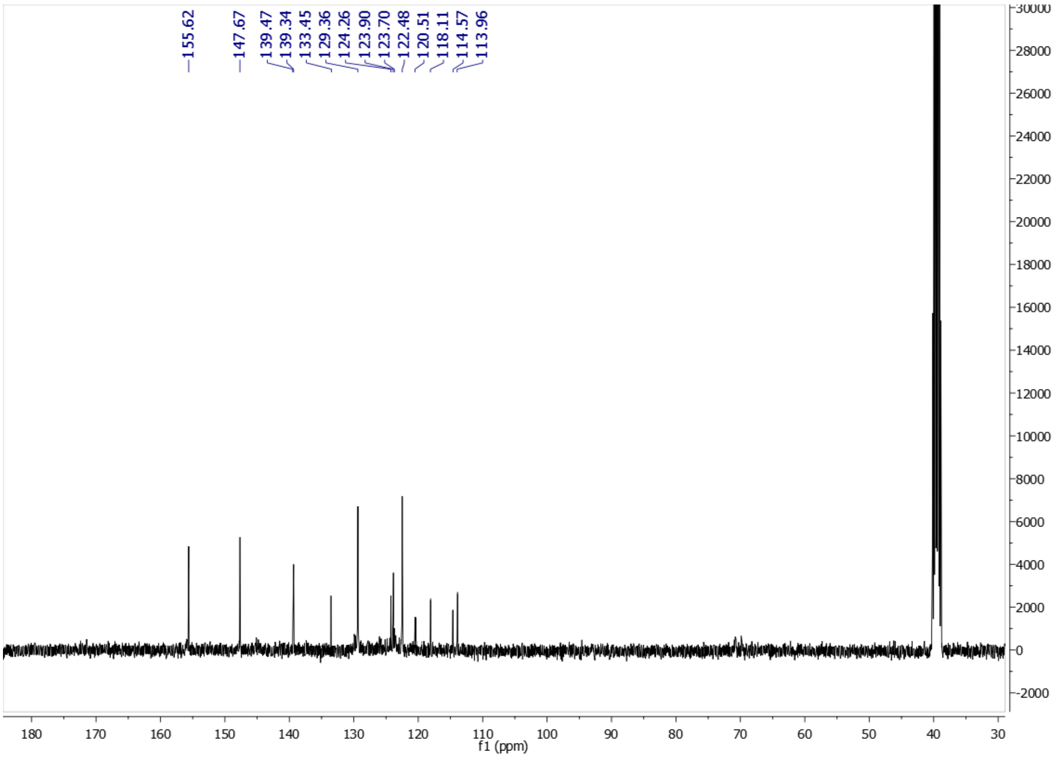

Figure S42. ^{13}C NMR of compound **22** at 101 MHz (DMSO-*d*6)

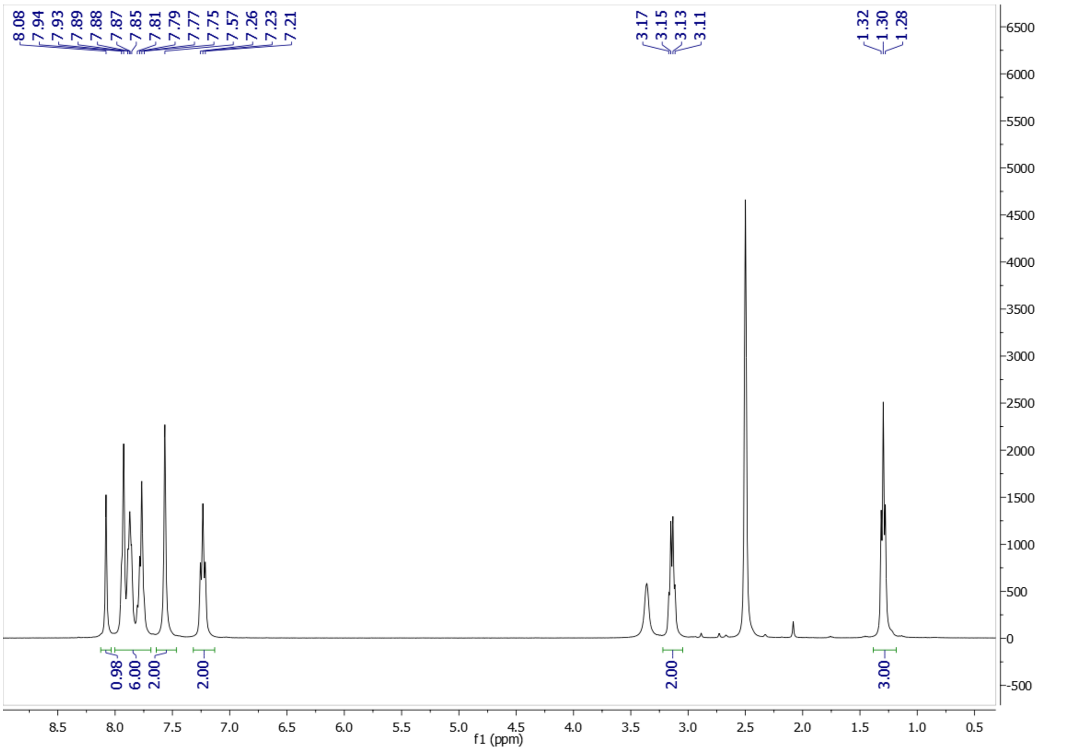

Figure S43. ^{1}H NMR of compound **23** at 400 MHz (DMSO-*d₆*)

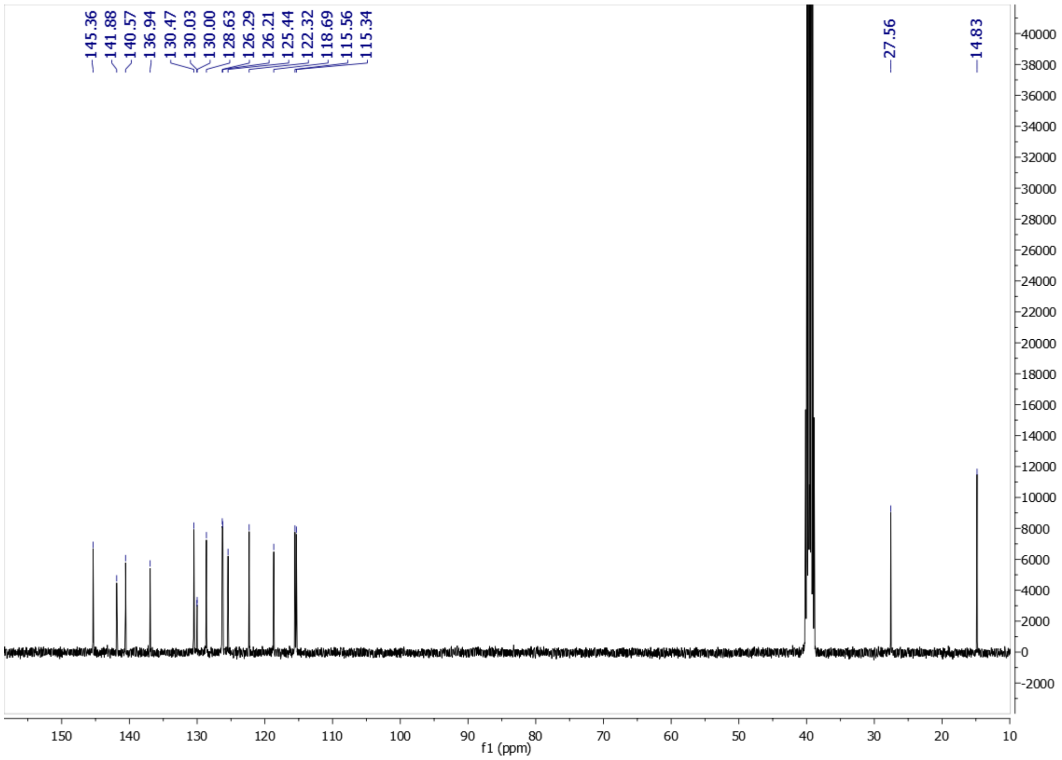

Figure S44. ^{13}C NMR of compound **23** at 101 MHz (DMSO-*d₆*)

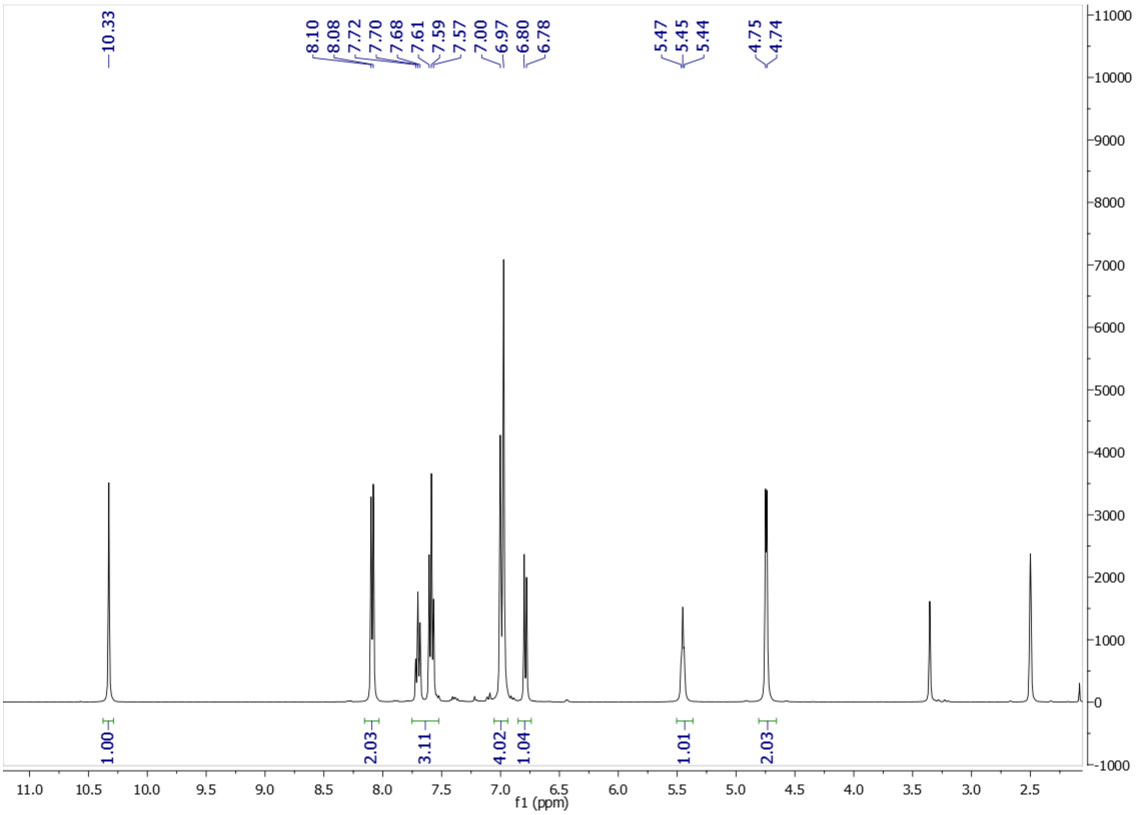

Figure S45. ^{1}H NMR of compound **25** at 400 MHz (DMSO-*d*$_6$)

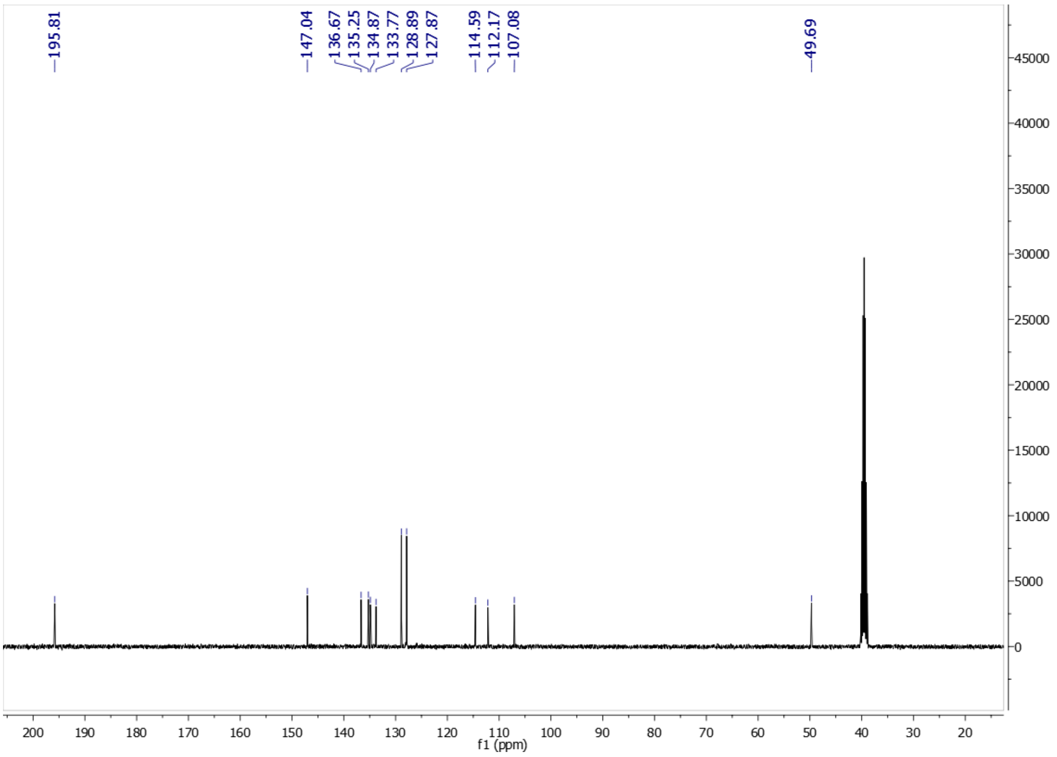

Figure S46. ^{13}C NMR of compound **25** at 101 MHz (DMSO-*d*$_6$)

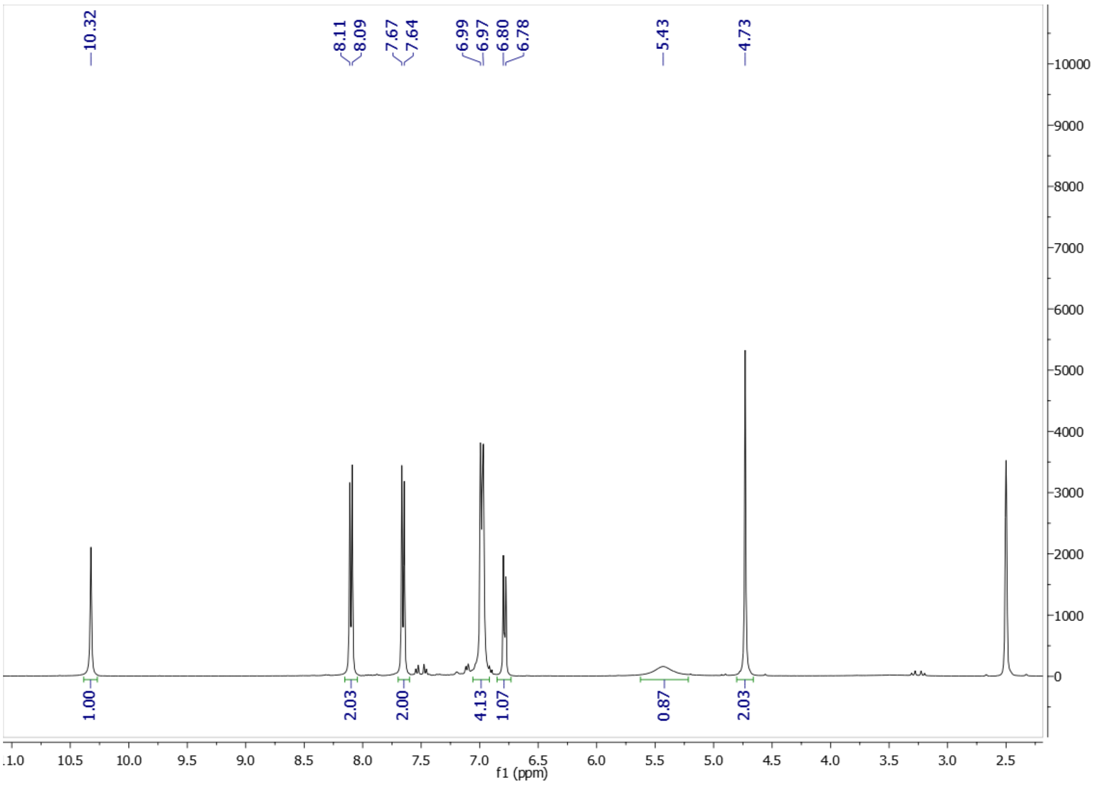

Figure S47. ^{1}H NMR of compound **26** at 400 MHz (DMSO-d_6)

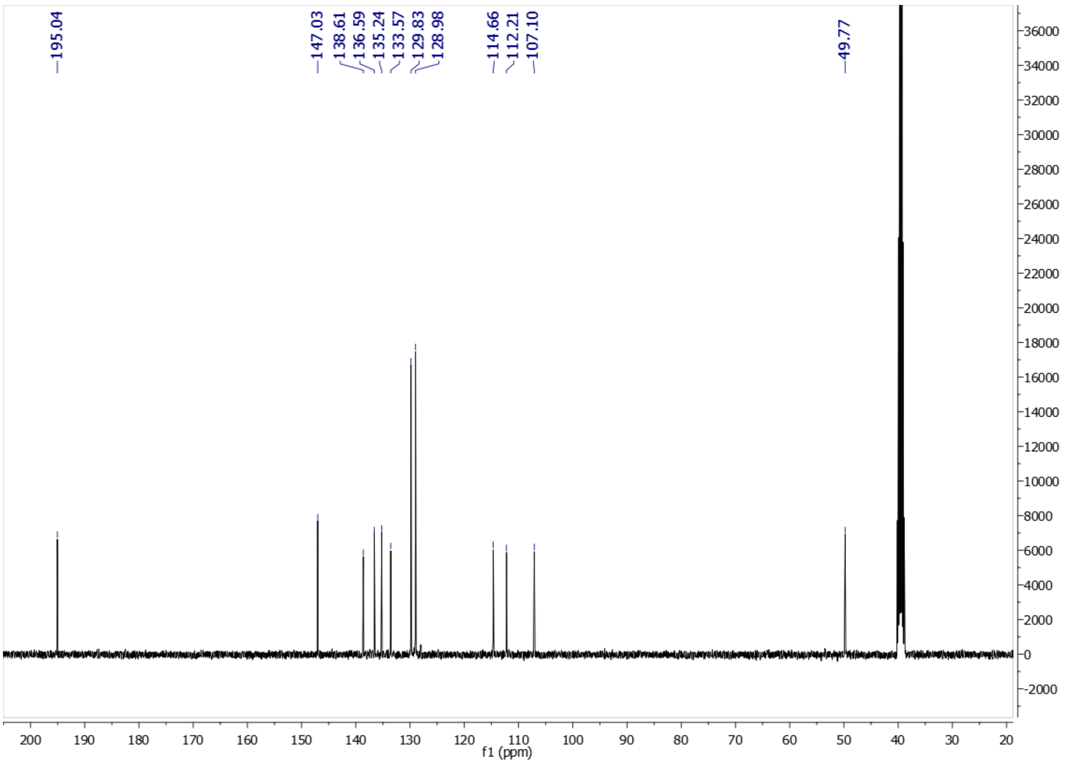

Figure S48. ^{13}C NMR of compound **26** at 101 MHz (DMSO-d_6)

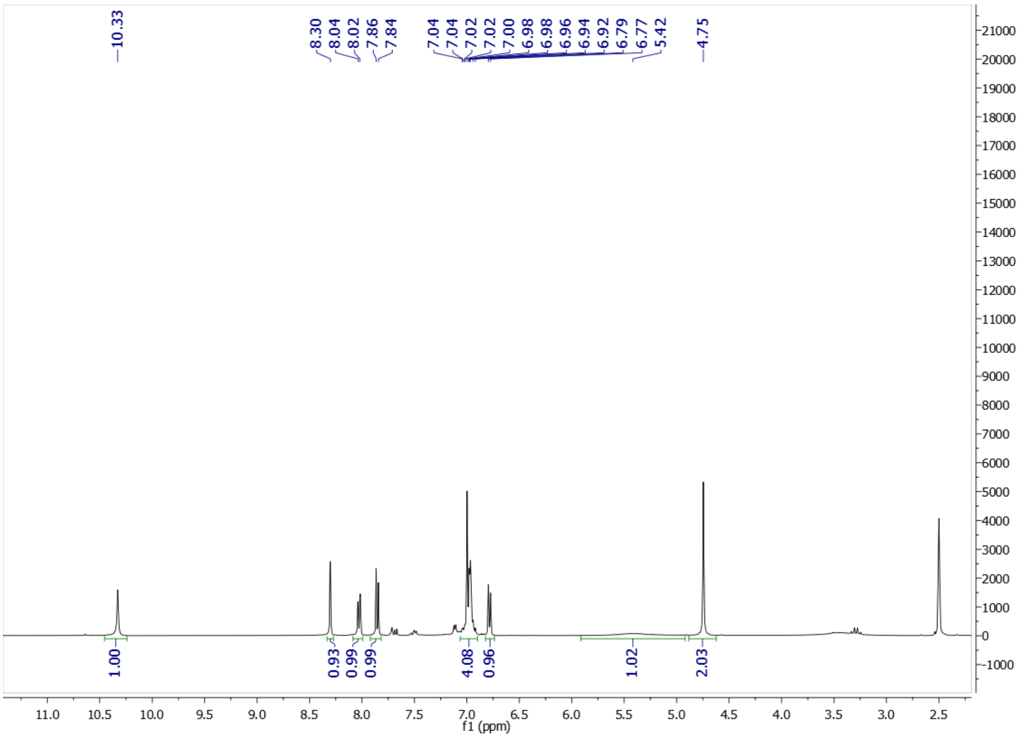

Figure S49. ^{1}H NMR of compound **27** at 400 MHz (DMSO-d_6)

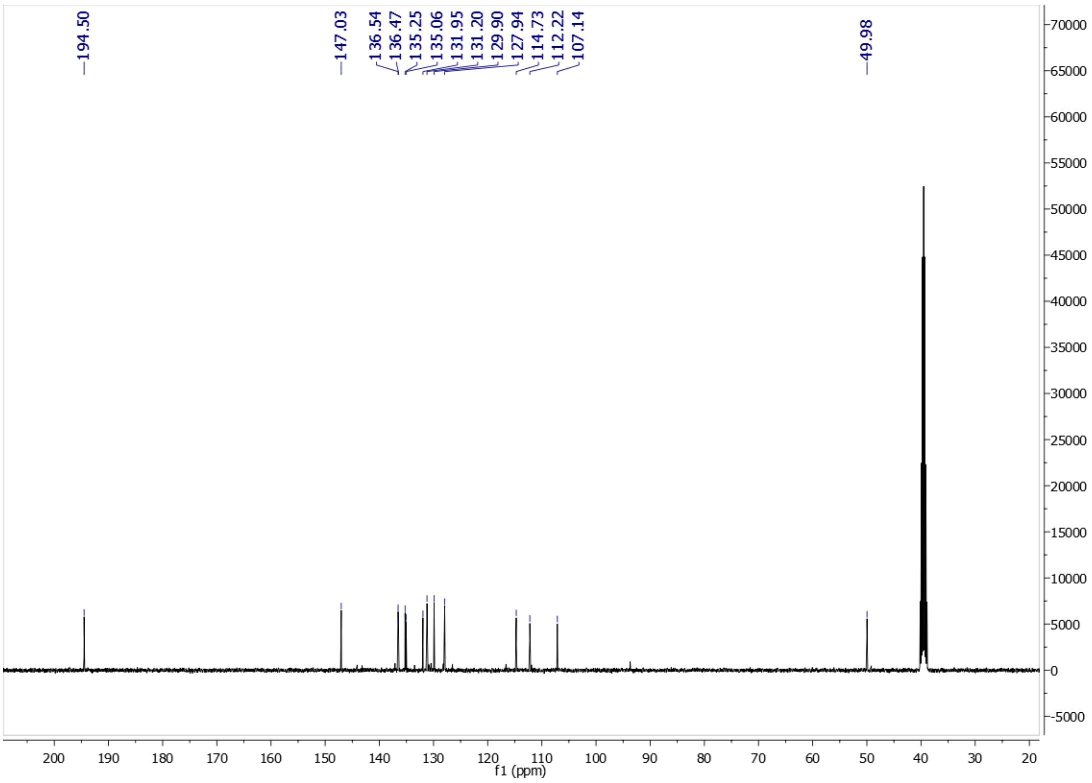

Figure S50. ^{13}C NMR of compound **27** at 101 MHz (DMSO-d_6)

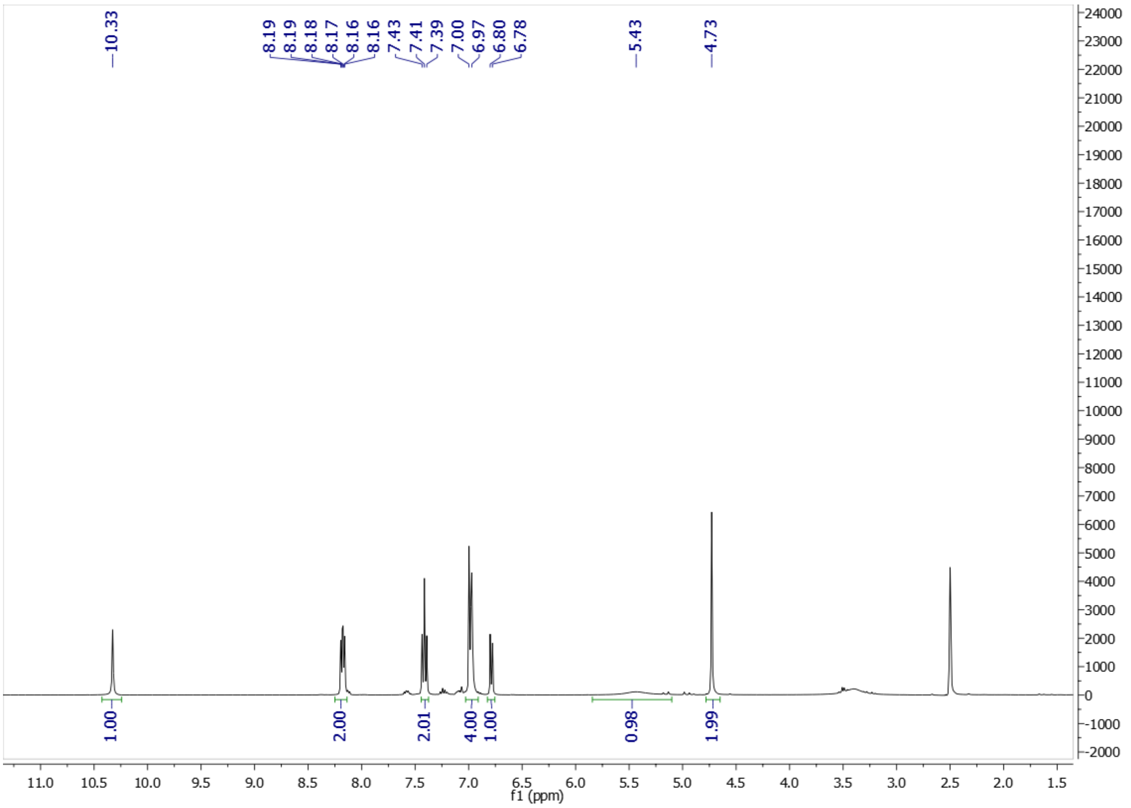

Figure S51. ^{1}H NMR of compound **28** at 400 MHz (DMSO-*d6*)

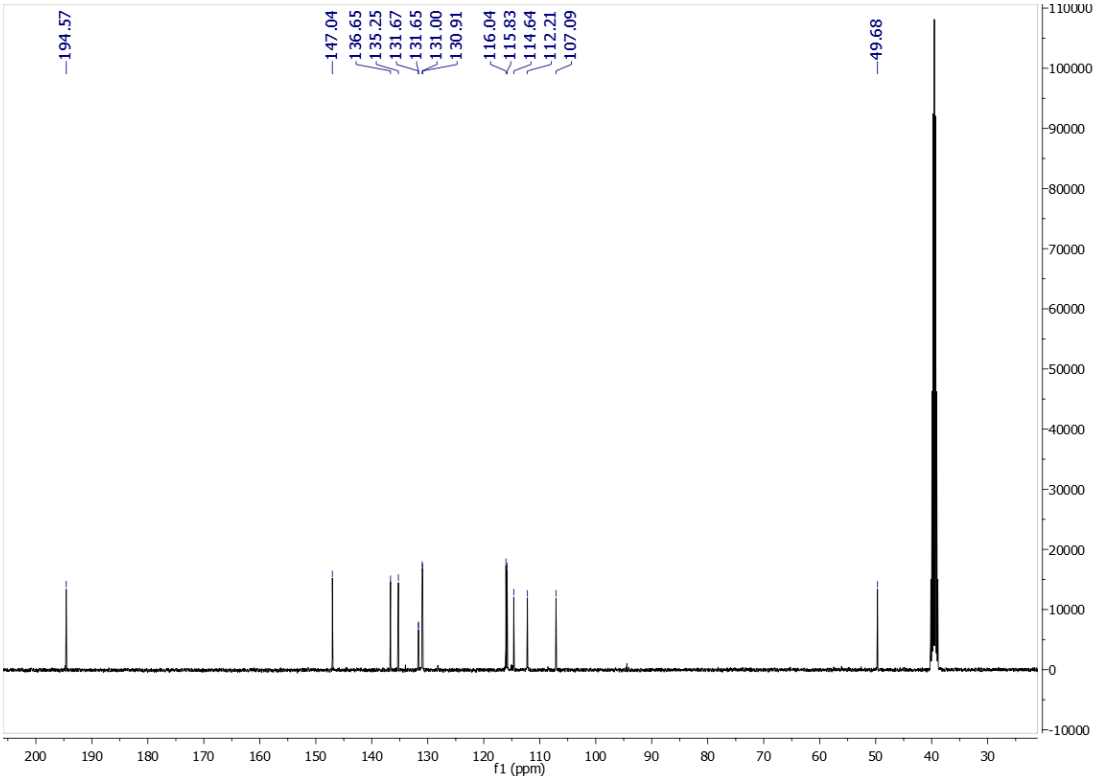

Figure S52. ^{13}C NMR of compound **28** at 101 MHz (DMSO-*d6*)

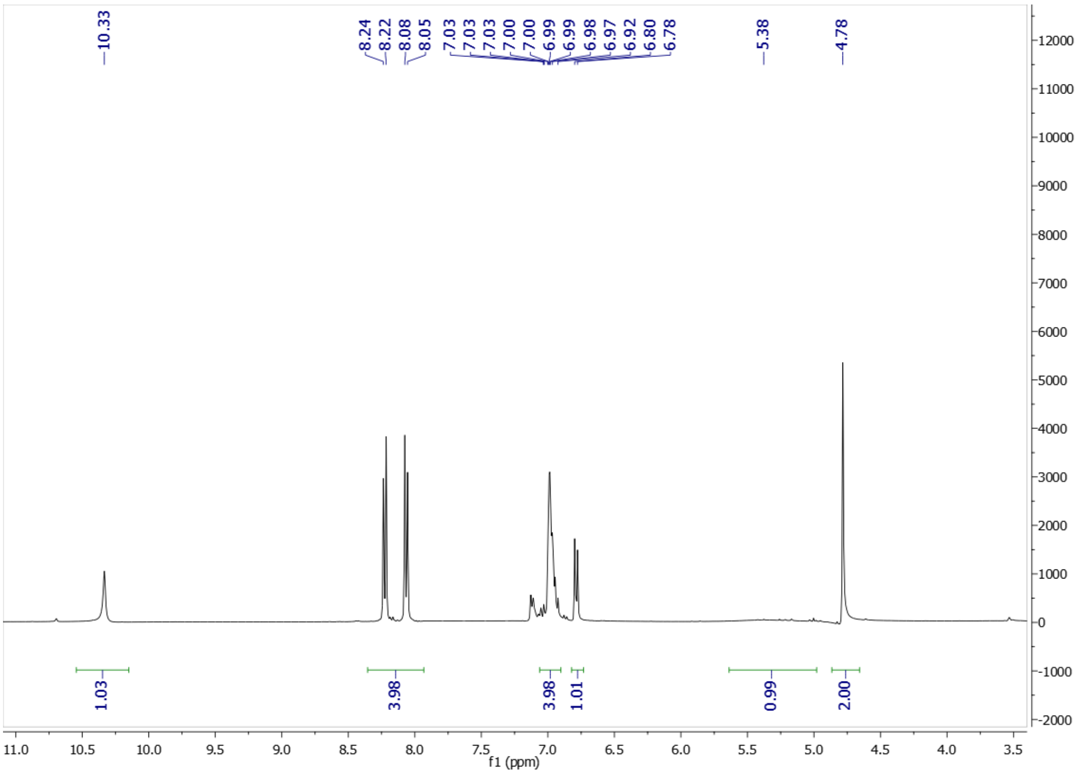

Figure S53. ^{1}H NMR of compound **29** at 400 MHz (DMSO-d_6)

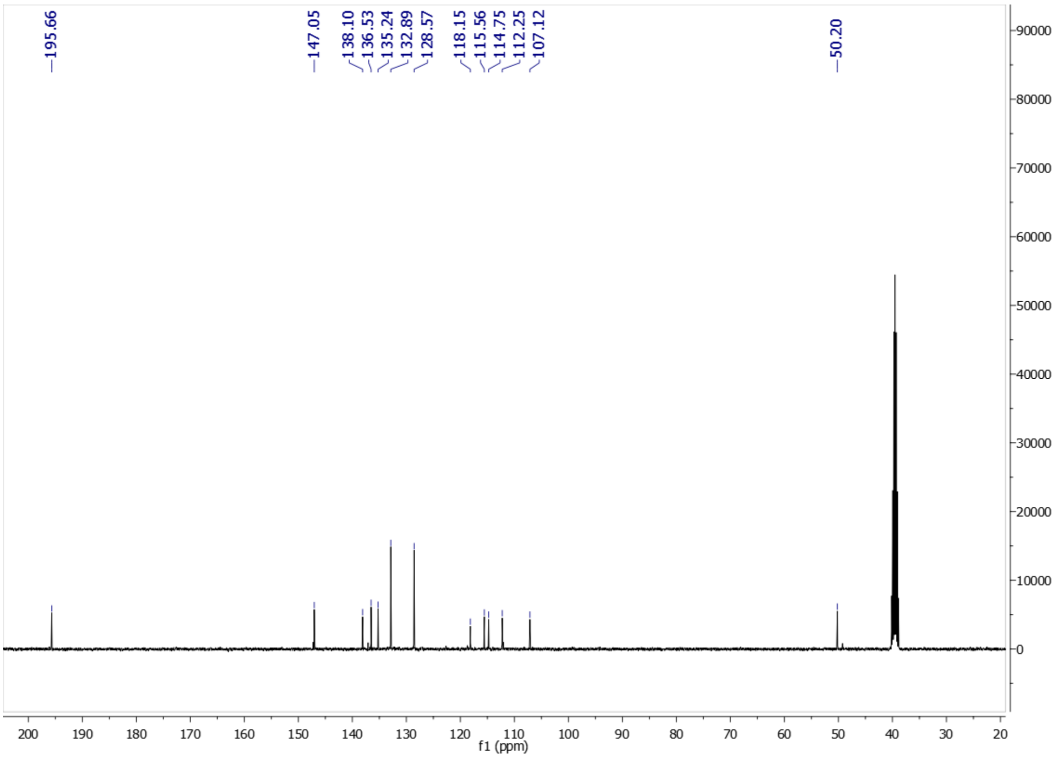

Figure S54. ^{13}C NMR of compound **29** at 101 MHz (DMSO-d_6)

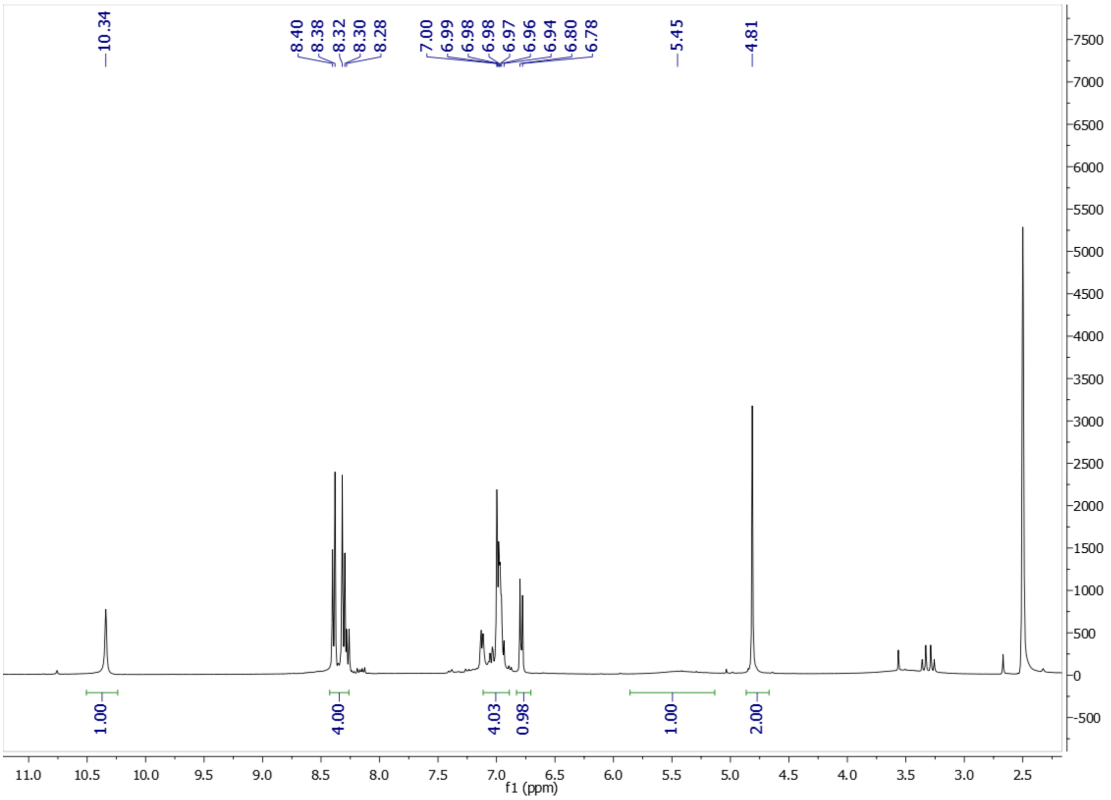

Figure S55. ^{1}H NMR of compound **30** at 400 MHz (DMSO-*d*$_6$)

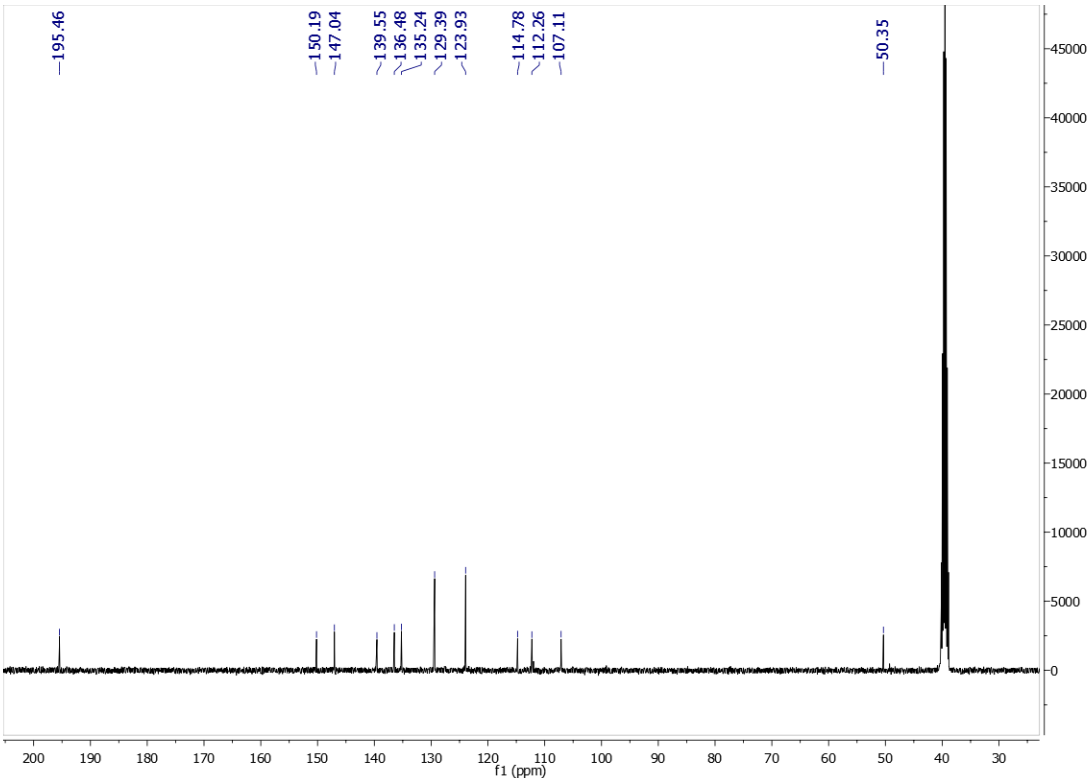

Figure S56. ^{13}C NMR of compound **30** at 101 MHz (DMSO-*d*$_6$)

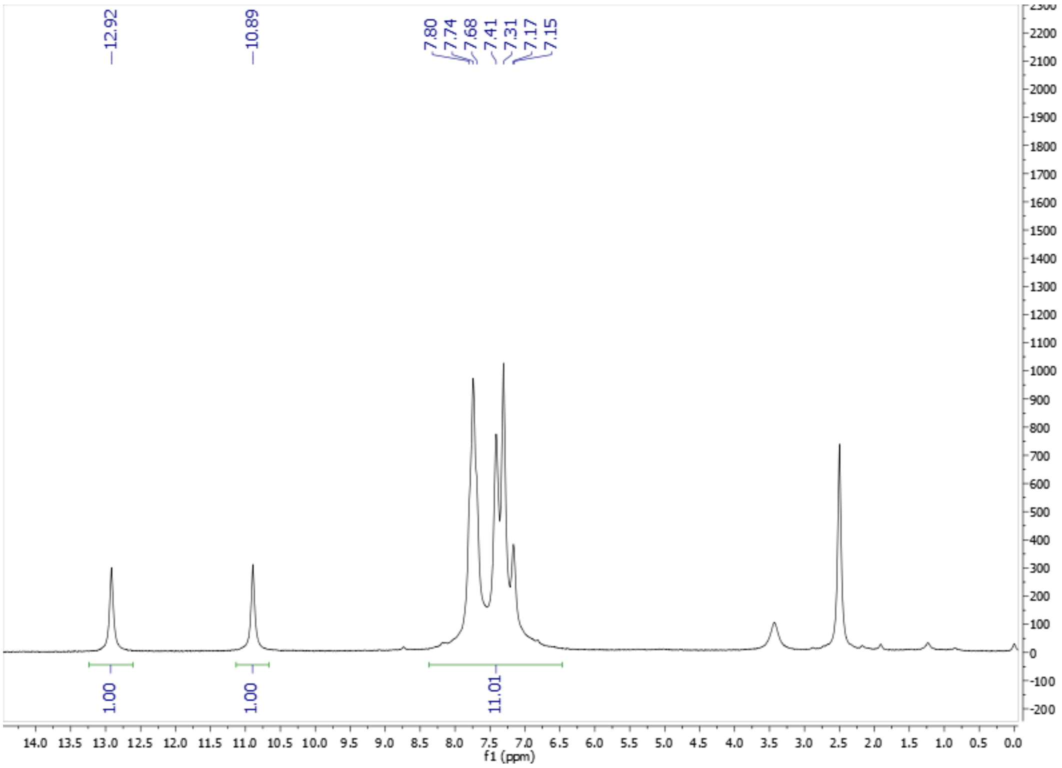

Figure S57. ^{1}H NMR of compound **31** at 400 MHz (DMSO-d_6)

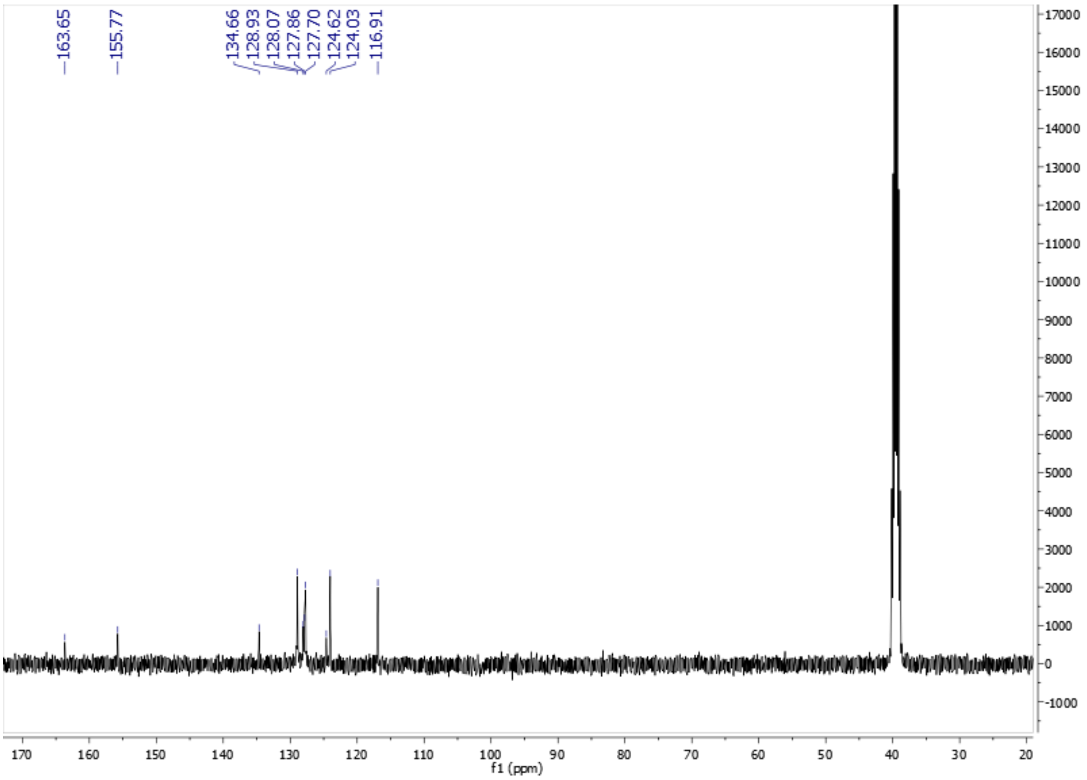

Figure S58. ^{13}C NMR of compound **31** at 101 MHz (DMSO-d_6)

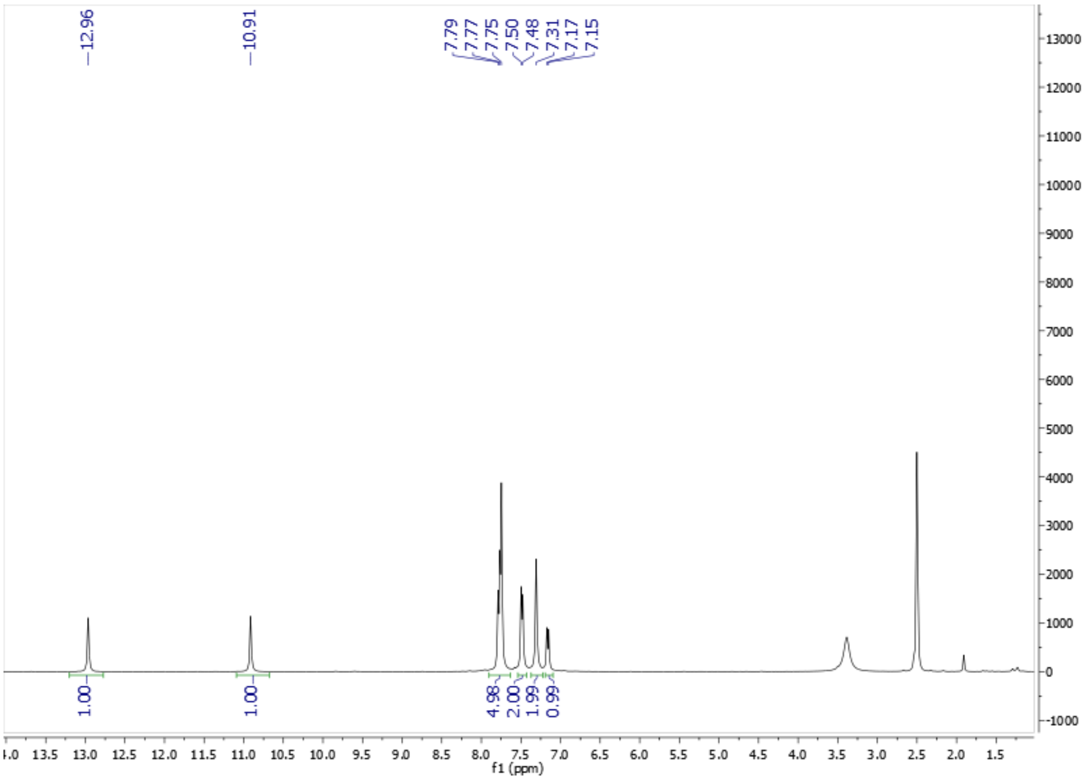

Figure S59. ^{1}H NMR of compound **32** at 400 MHz (DMSO-d_6)

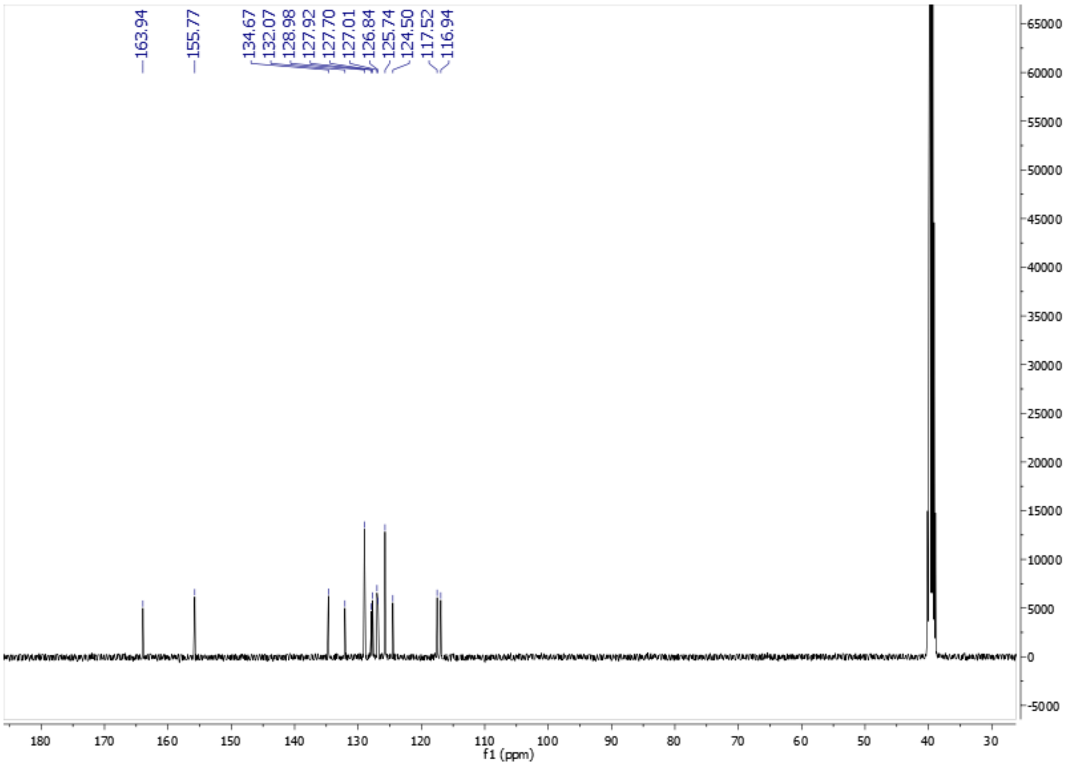

Figure S60. ^{13}C NMR of compound **32** at 101 MHz (DMSO-d_6)

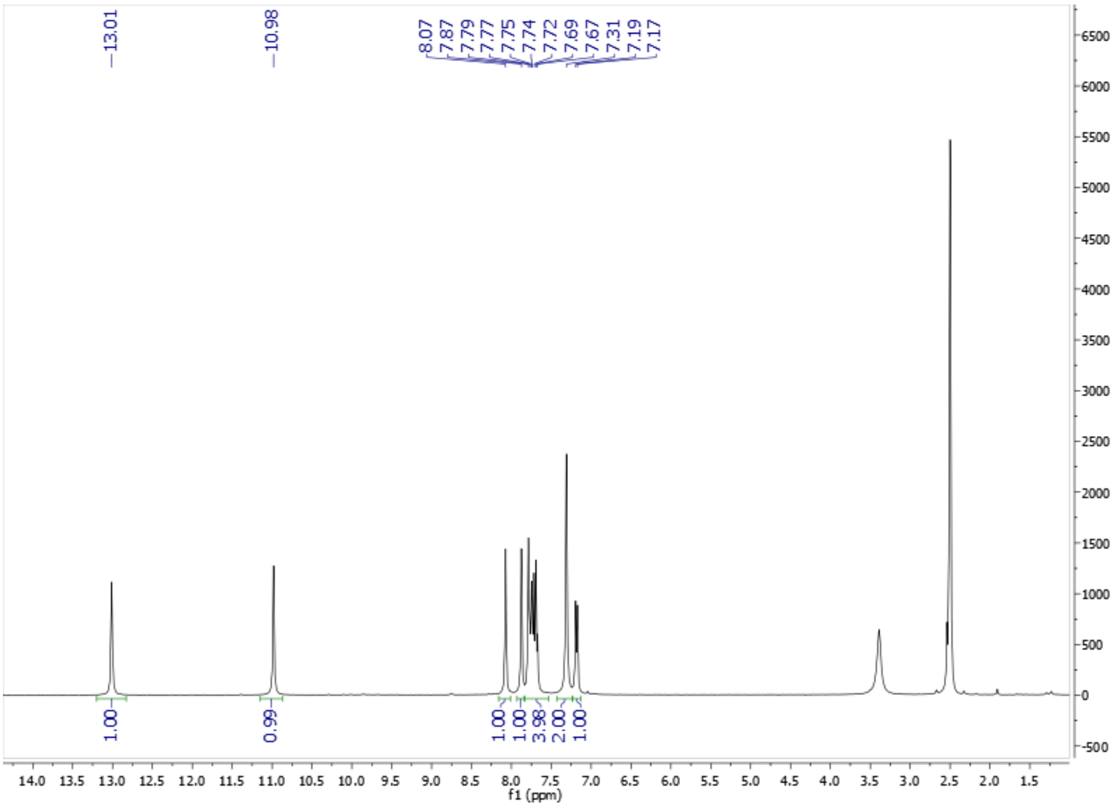

Figure S61. ^{1}H NMR of compound **33** at 400 MHz (DMSO-d_6)

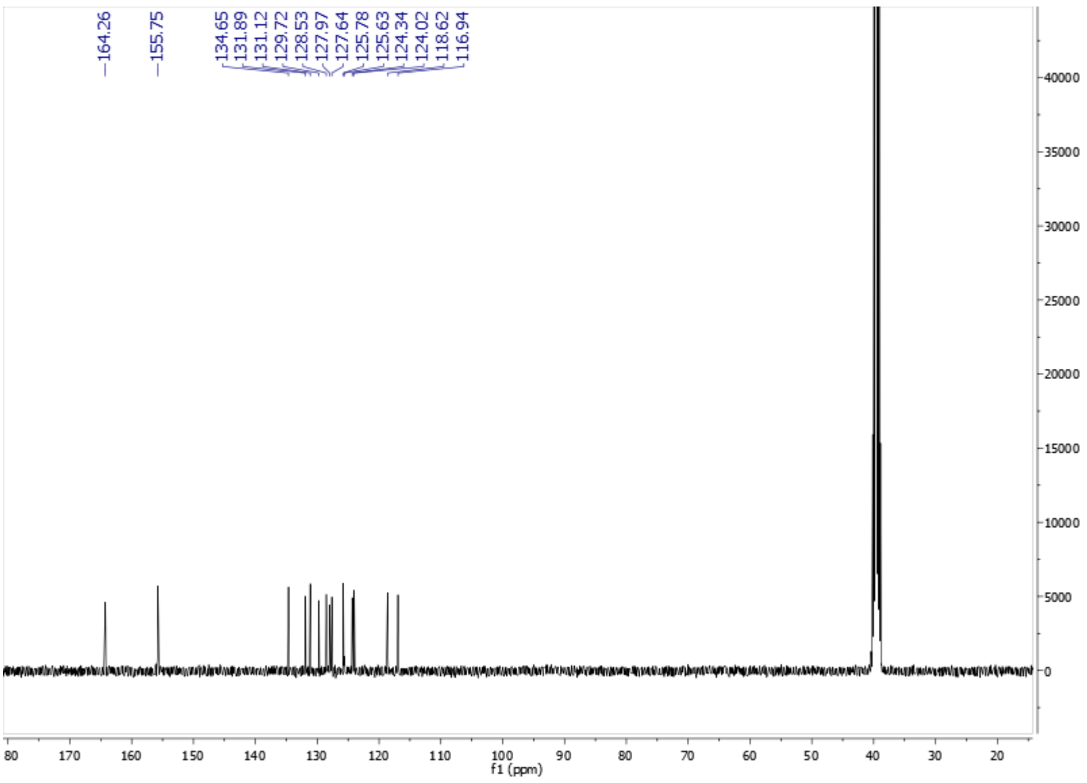

Figure S62. ^{13}C NMR of compound **33** at 101 MHz (DMSO-d_6)

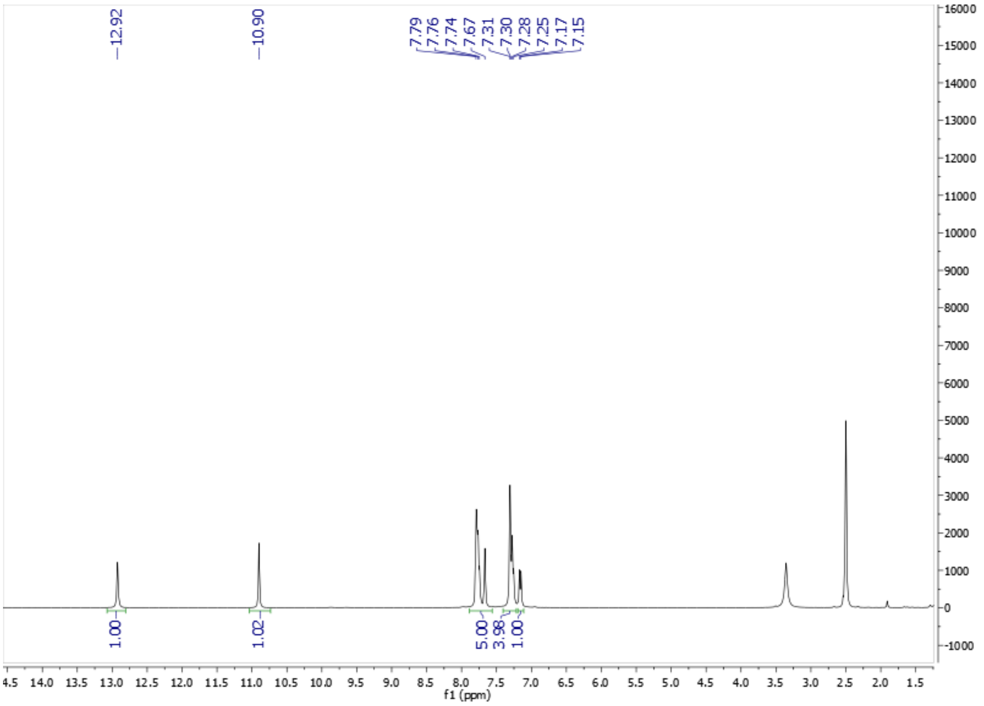

Figure S63. ^{1}H NMR of compound **34** at 400 MHz (DMSO-d_6)

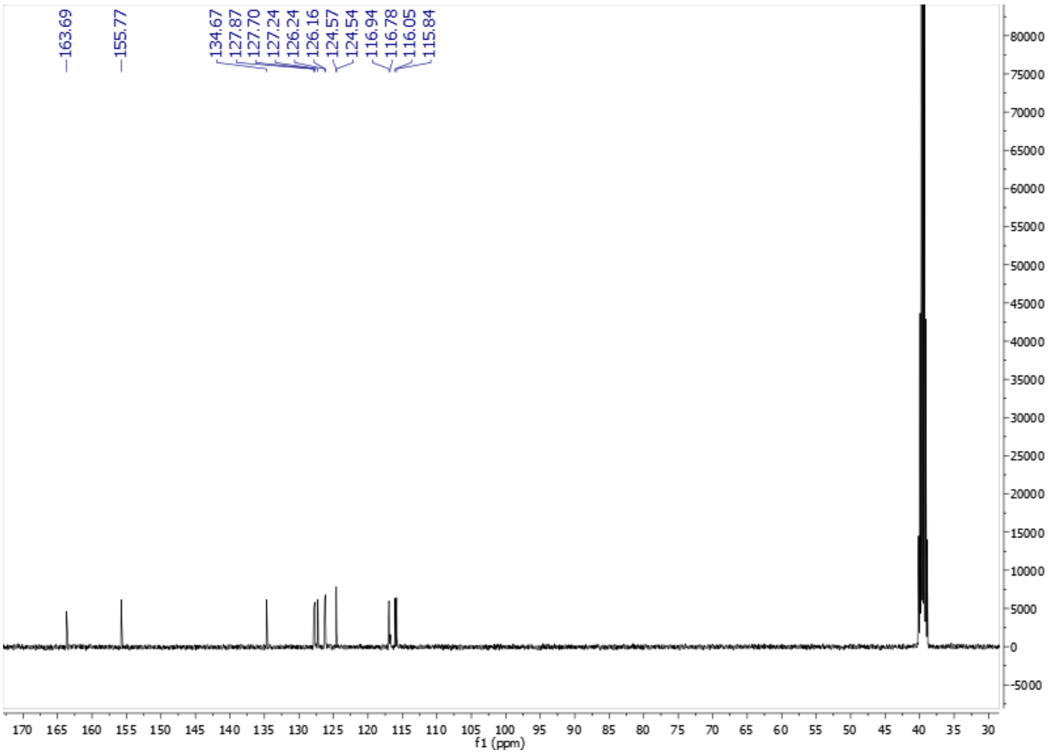

Figure S64. ^{13}C NMR of compound **34** at 101 MHz (DMSO-d_6)

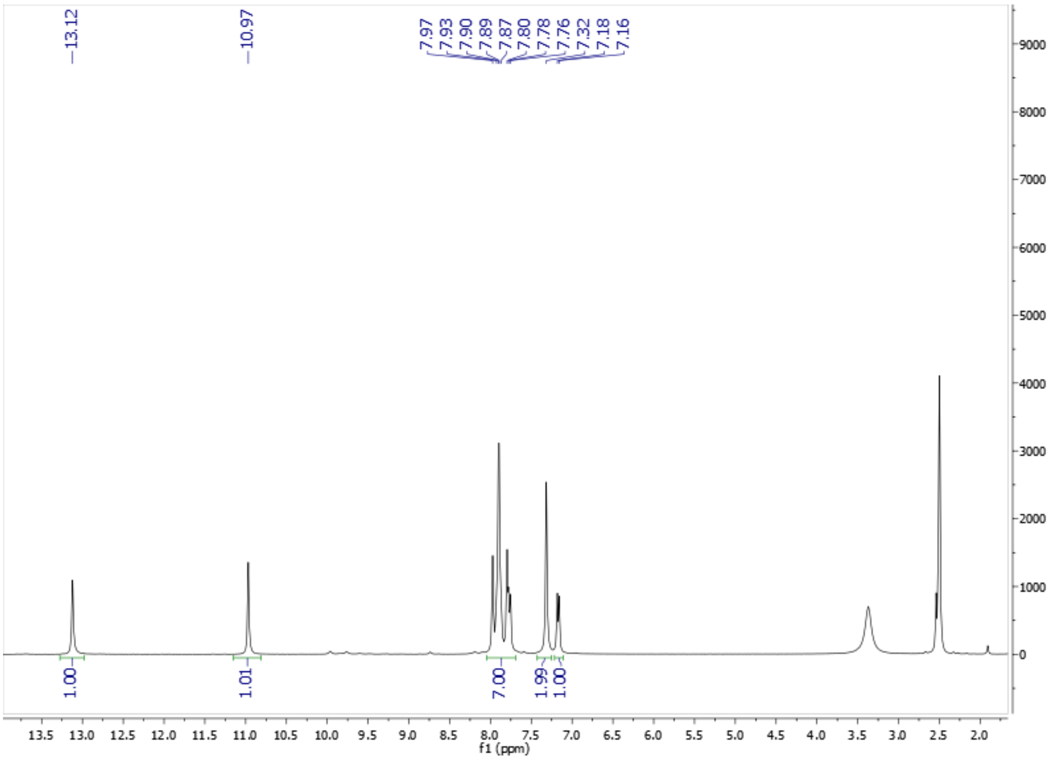

Figure S65. ^{1}H NMR of compound **35** at 400 MHz (DMSO-d_6)

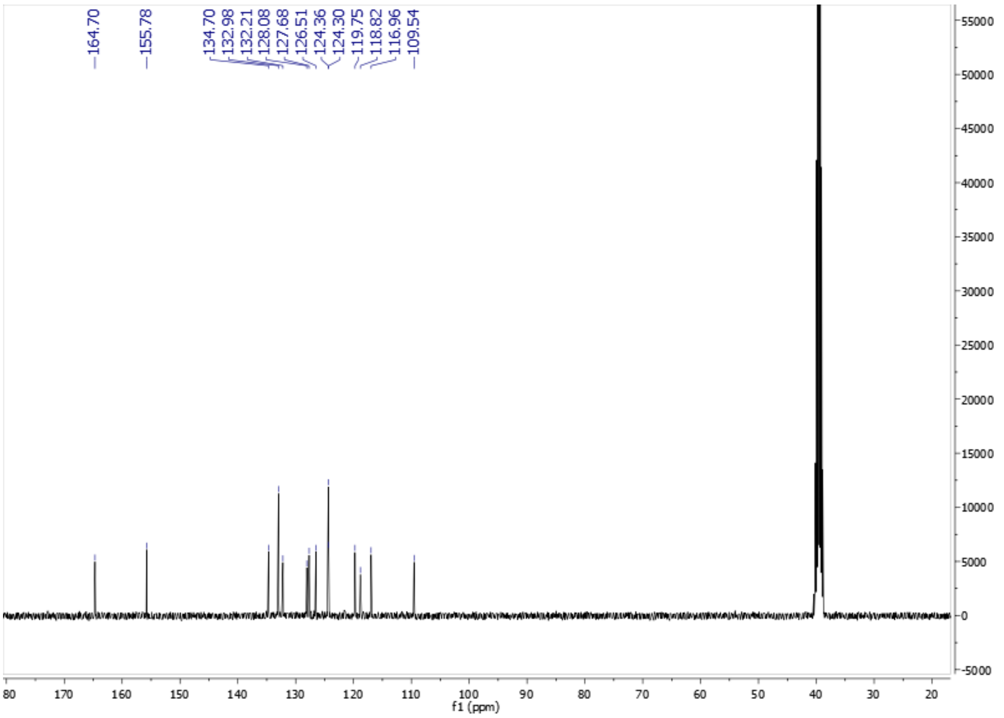

Figure S66. ^{13}C NMR of compound **35** at 101 MHz (DMSO-d_6)

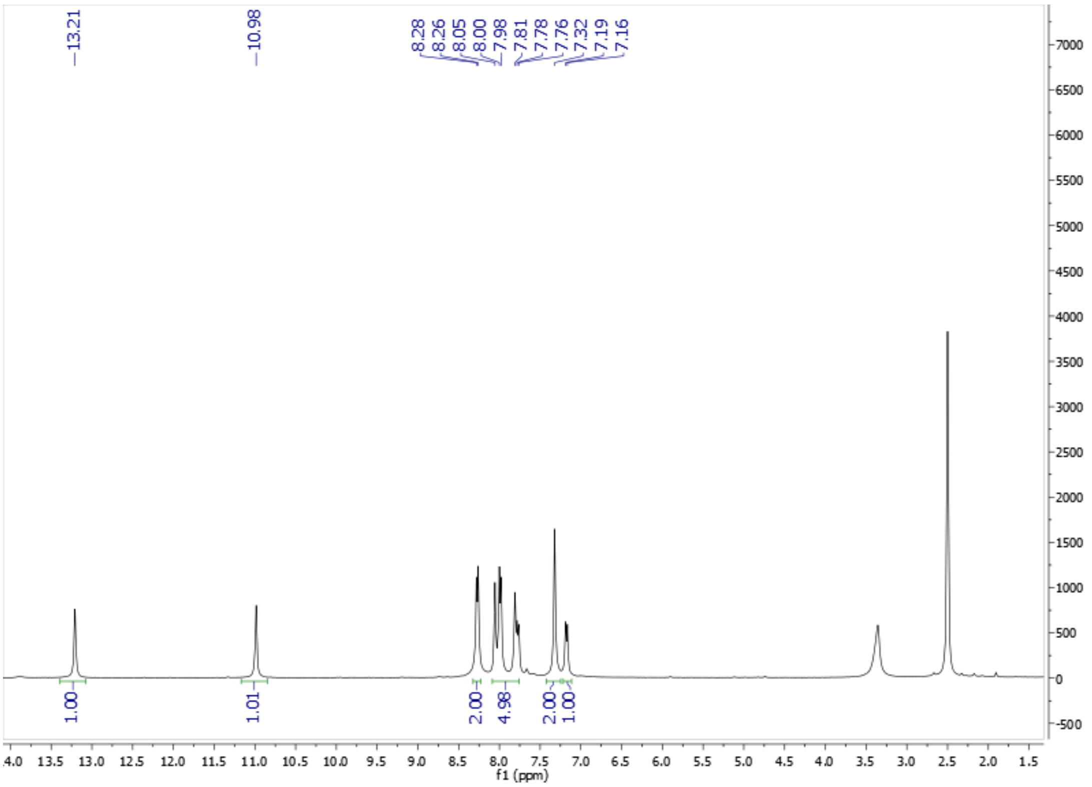

Figure S67. ^{1}H NMR of compound **36** at 400 MHz (DMSO-*d*6)

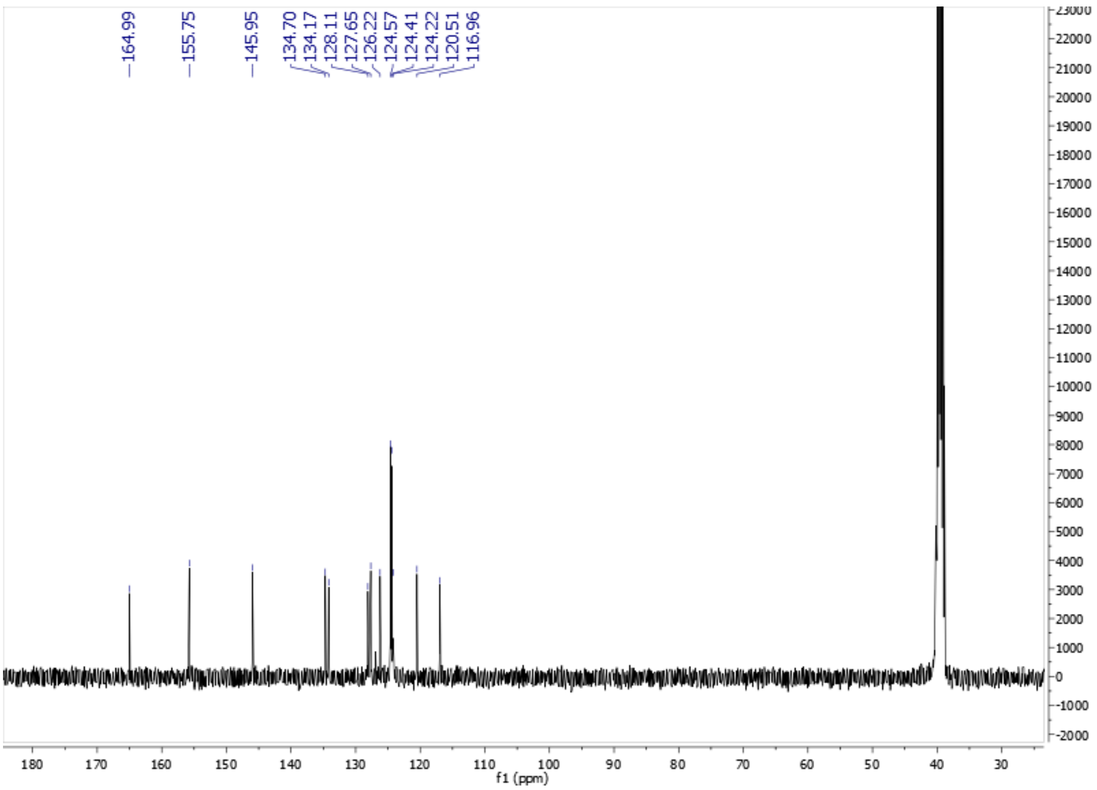

Figure S68. ^{13}C NMR of compound **36** at 101 MHz (DMSO-*d*6)

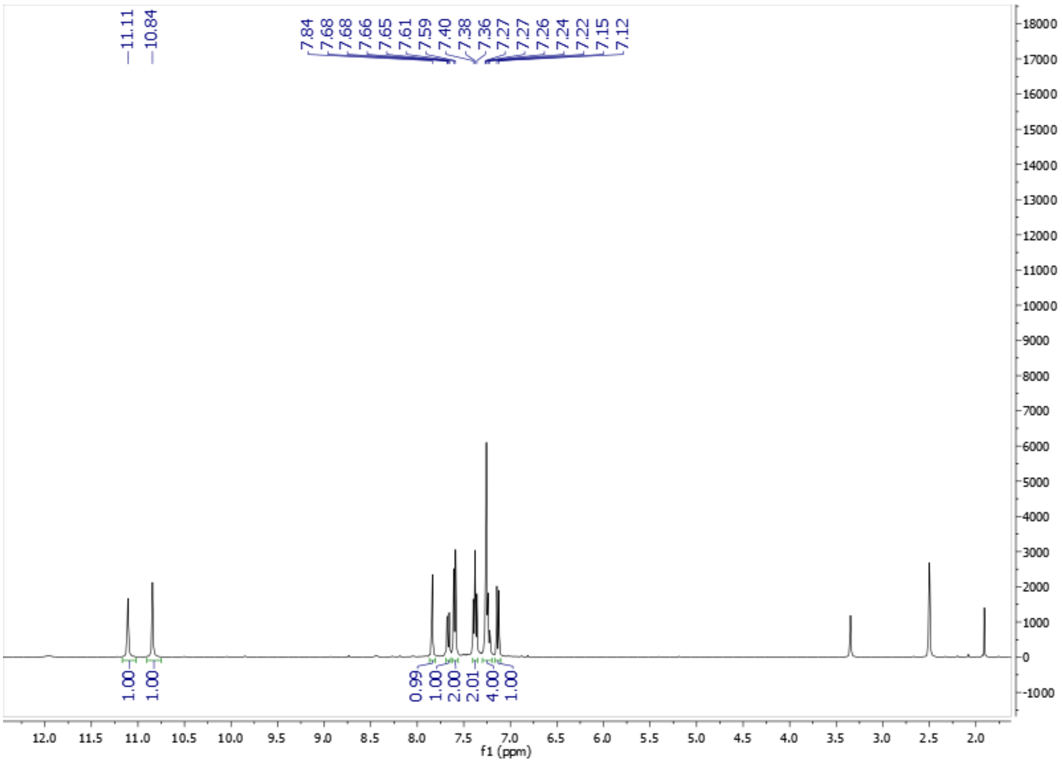

Figure S69. ^{1}H NMR of compound **37** at 400 MHz (DMSO-d_6)

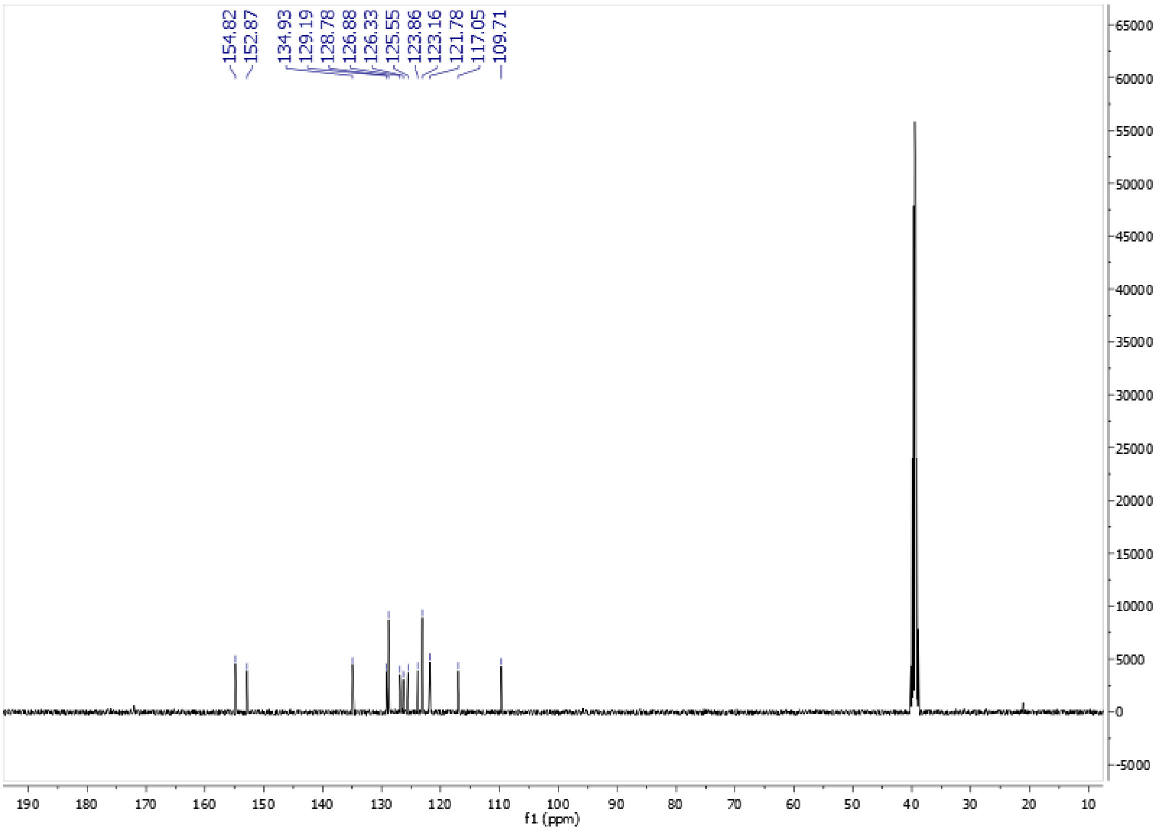

Figure S70. ^{13}C NMR of compound **37** at 101 MHz (DMSO-d_6)

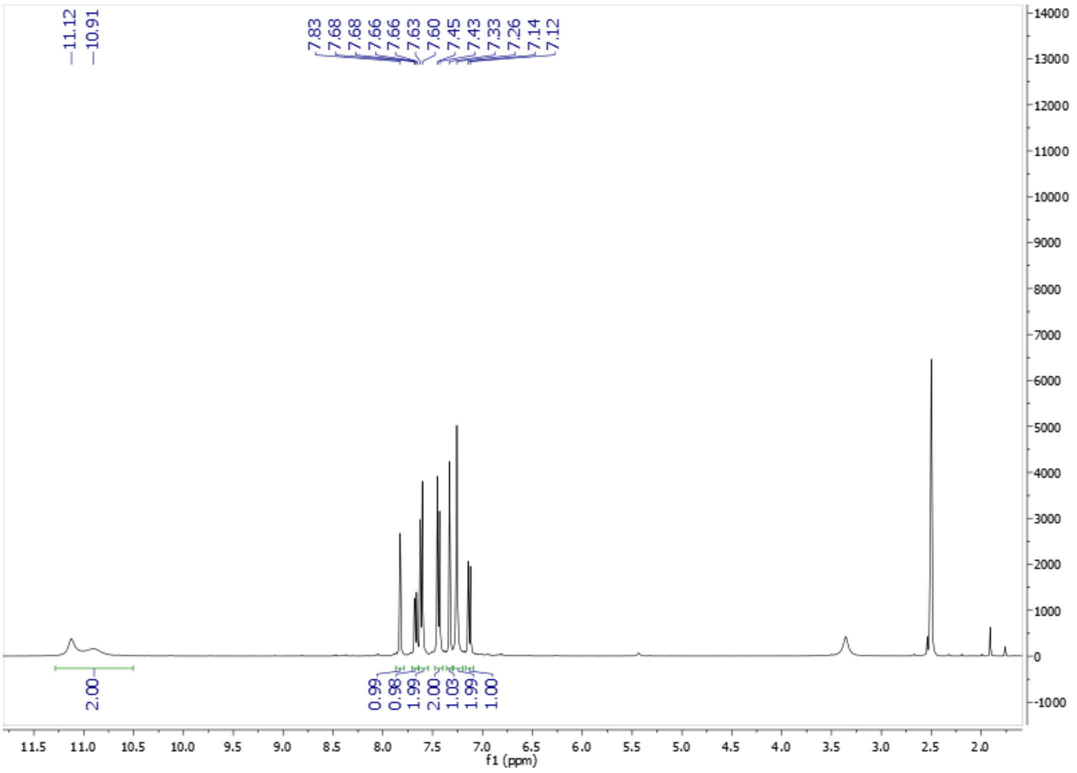

Figure S71. ^{1}H NMR of compound **38** at 400 MHz (DMSO-d_6)

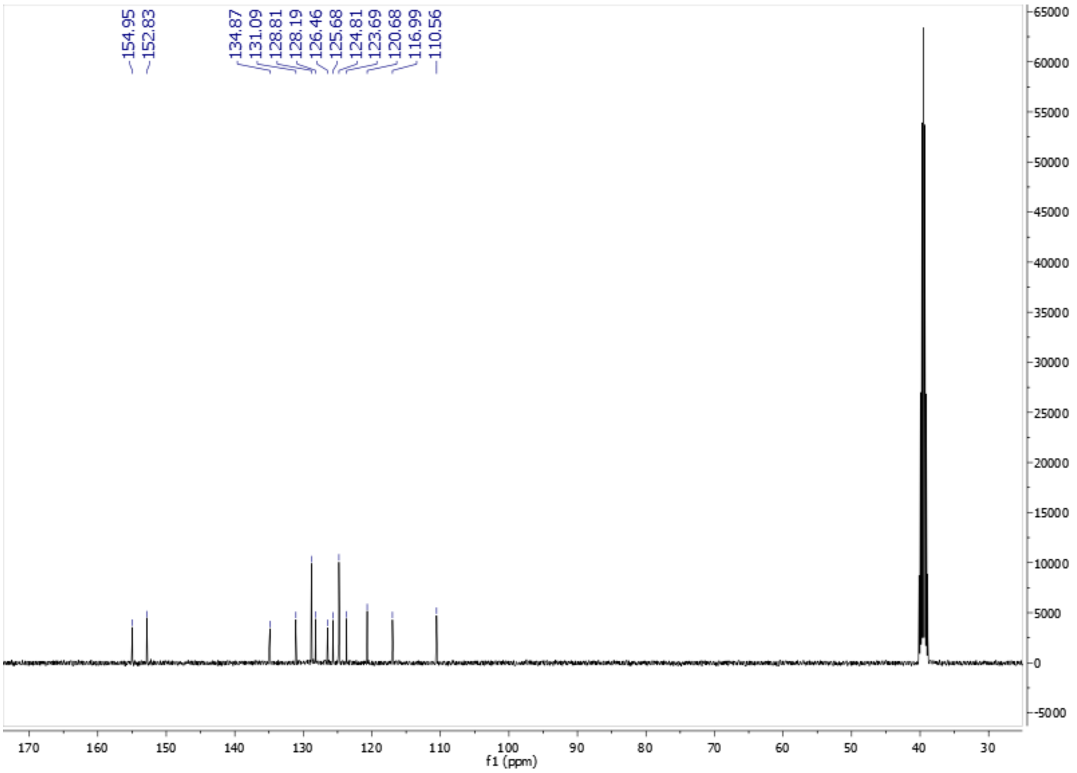

Figure S72. ^{13}C NMR of compound **38** at 101 MHz (DMSO-d_6)

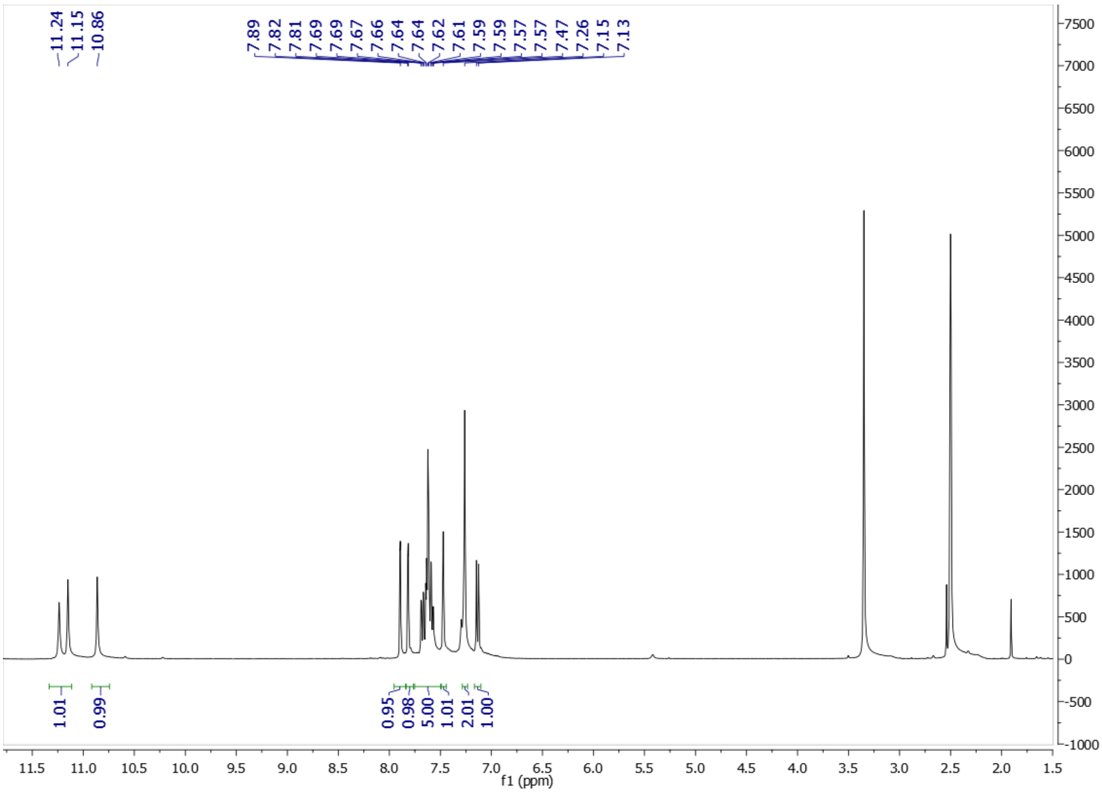

Figure S73. ^{1}H NMR of compound **39** at 400 MHz (DMSO-*d*6)

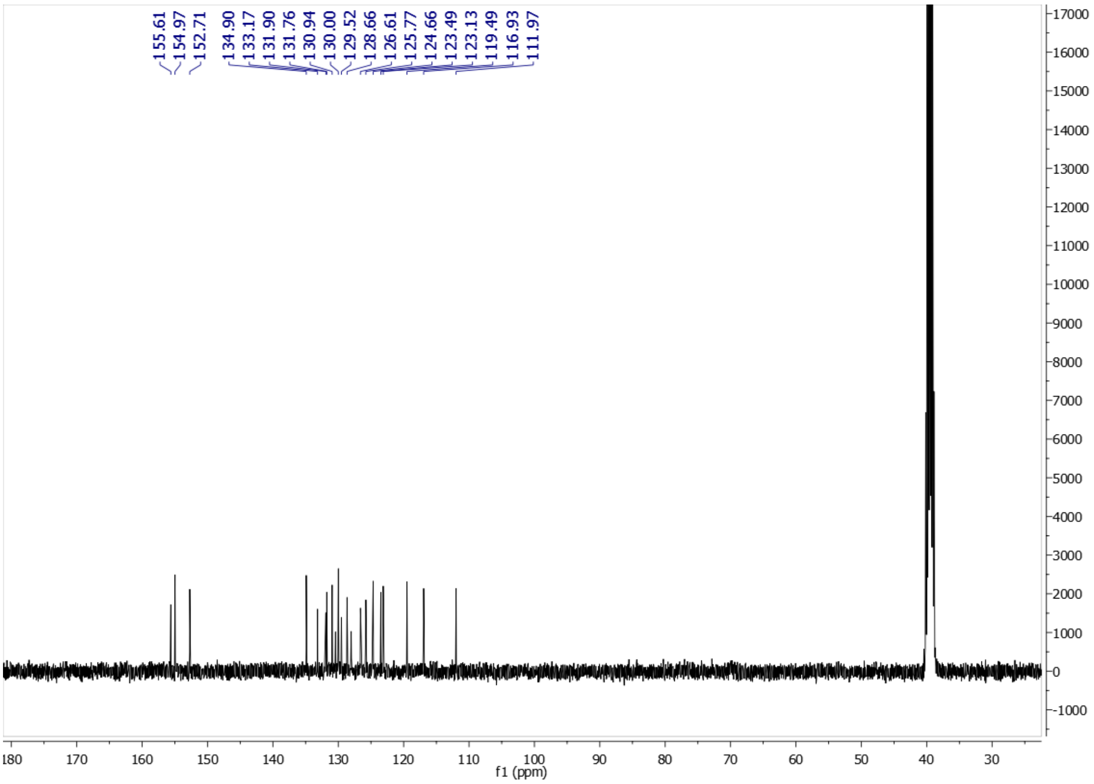

Figure S74. ^{13}C NMR of compound **39** at 101 MHz (DMSO-*d*6)

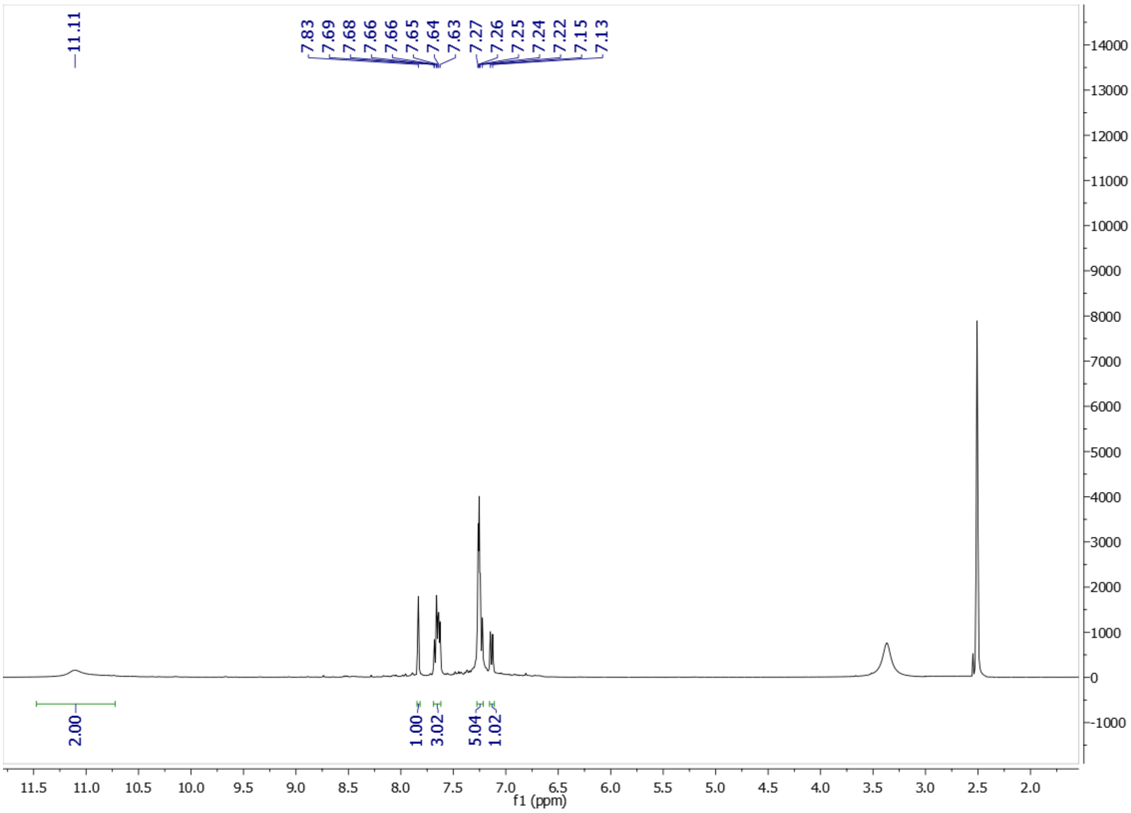

Figure S75. ^{1}H NMR of compound **40** at 400 MHz (DMSO-*d*$_6$)

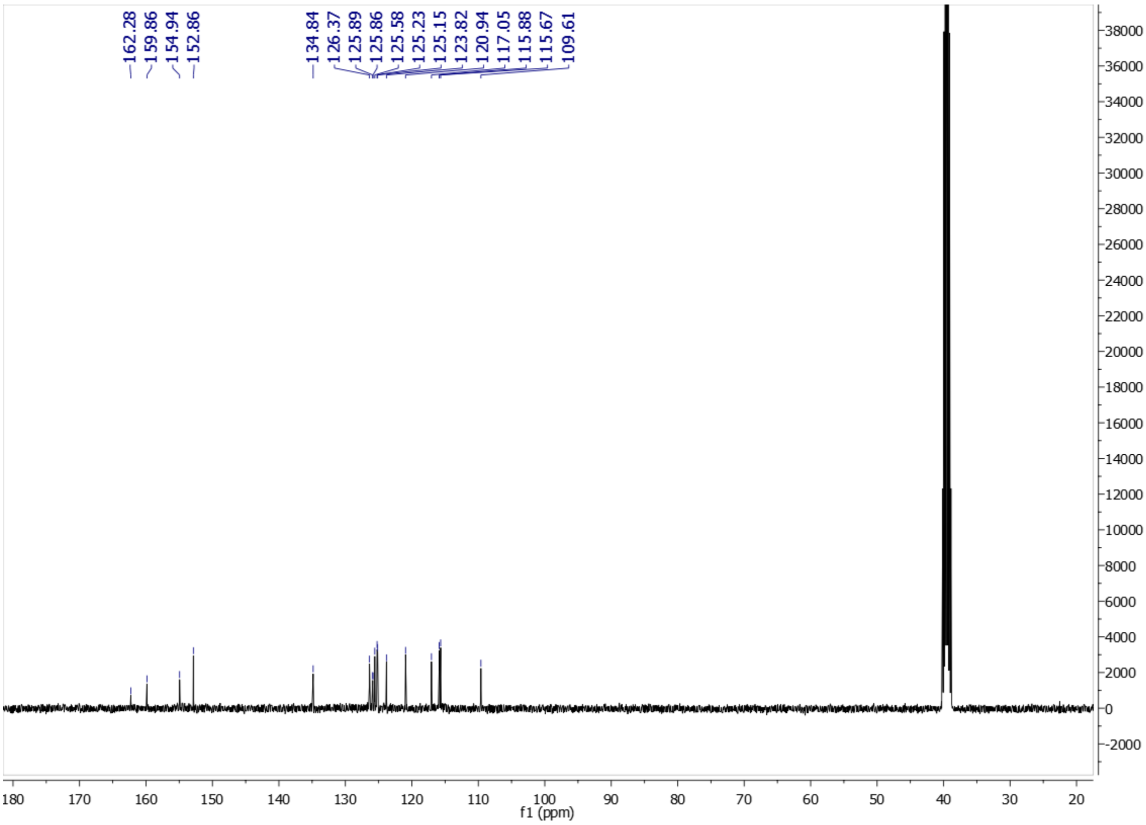

Figure S76. ^{13}C NMR of compound **40** at 101 MHz (DMSO-*d*$_6$)

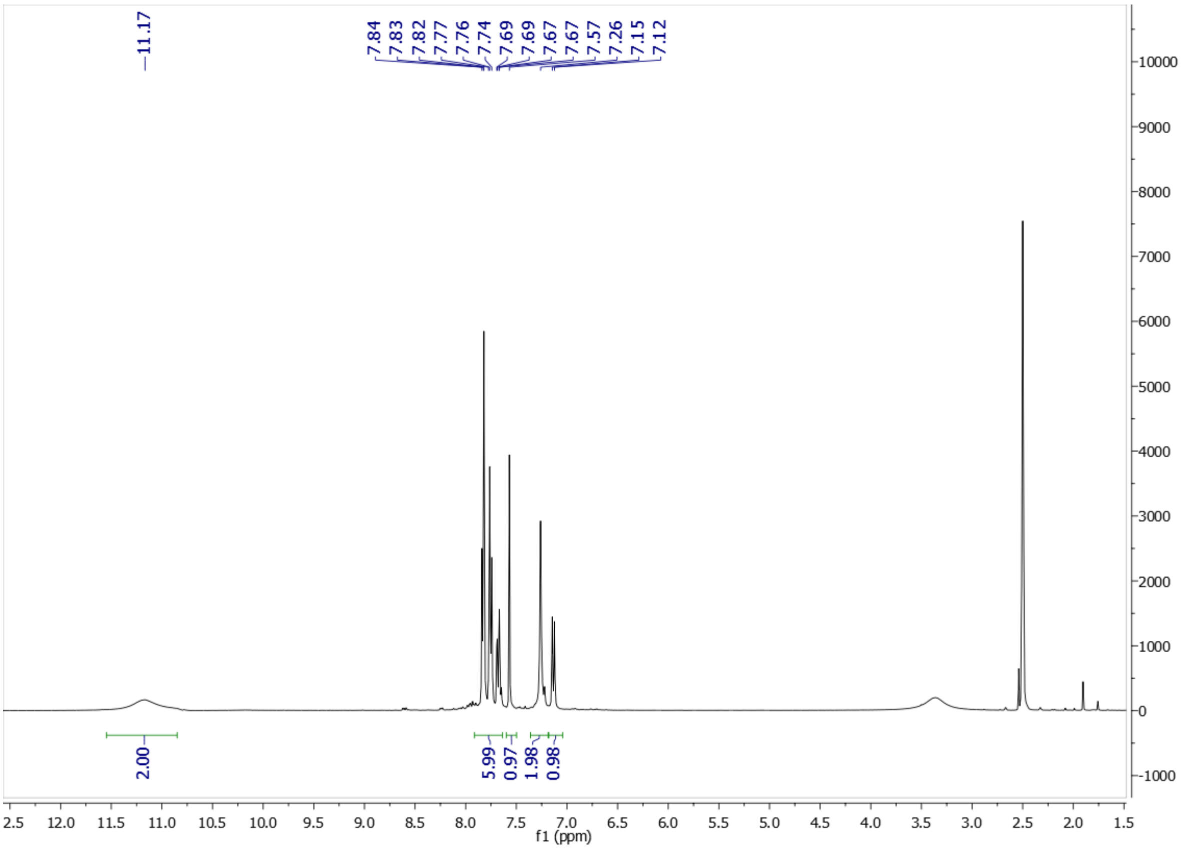

Figure S77. ^{1}H NMR of compound **41** at 400 MHz (DMSO-d_6)

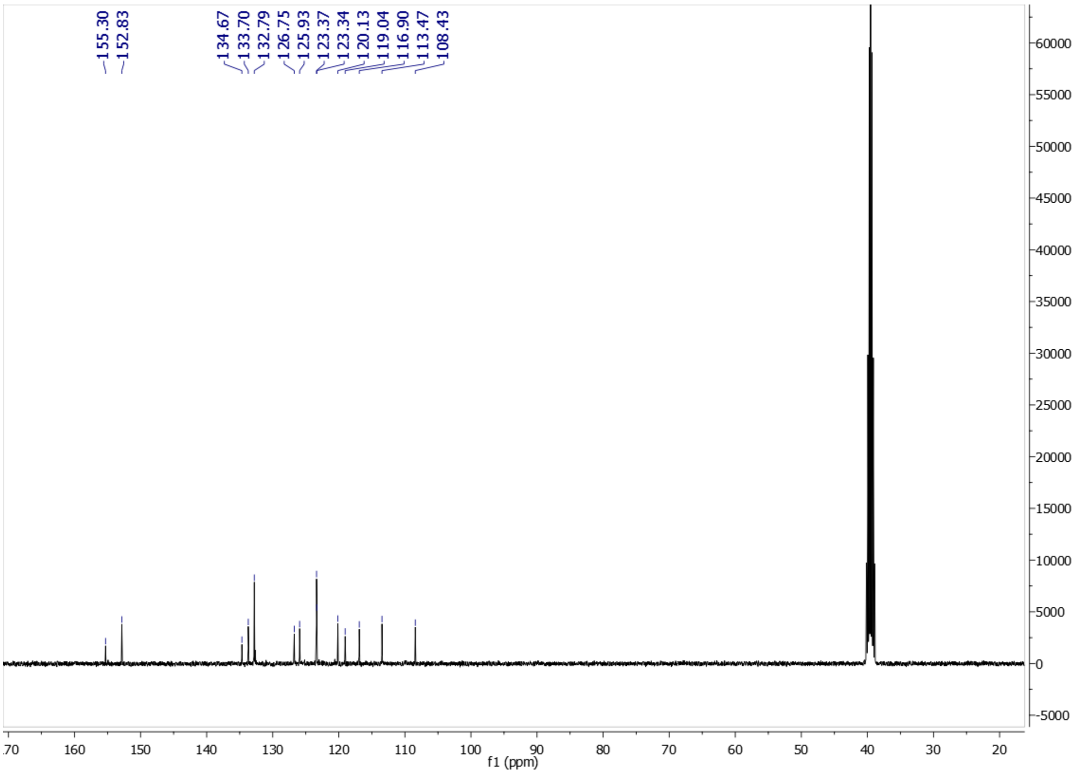

Figure S78. ^{13}C NMR of compound **41** at 101 MHz (DMSO-d_6)

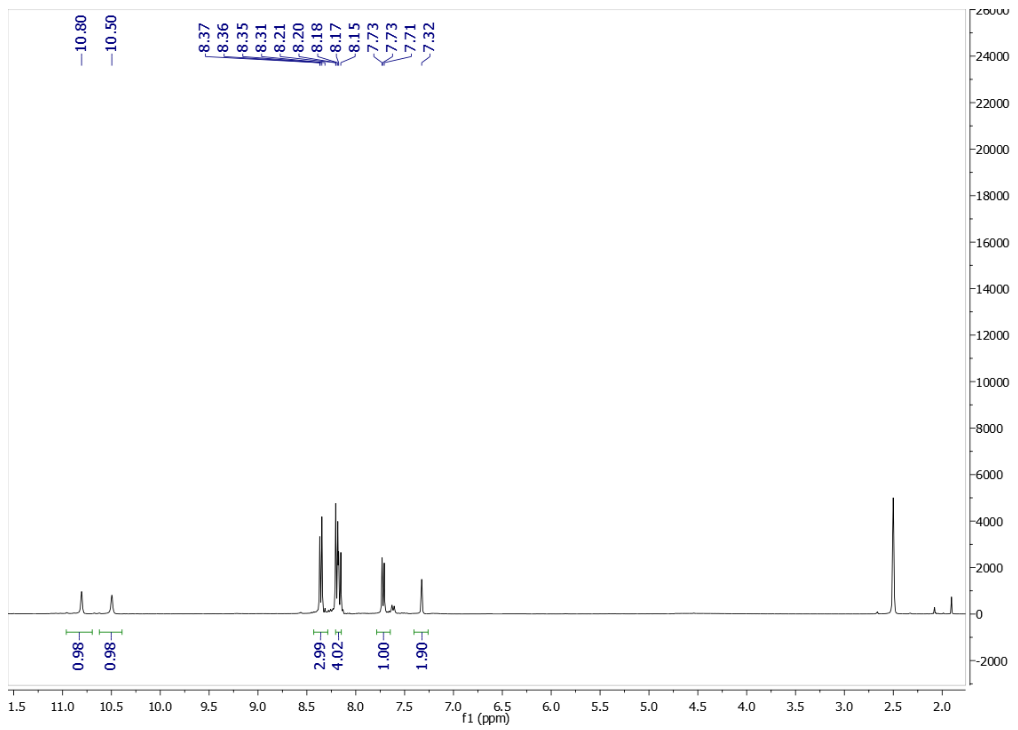

Figure S79. ^{1}H NMR of compound **42** at 400 MHz (DMSO-d_6)

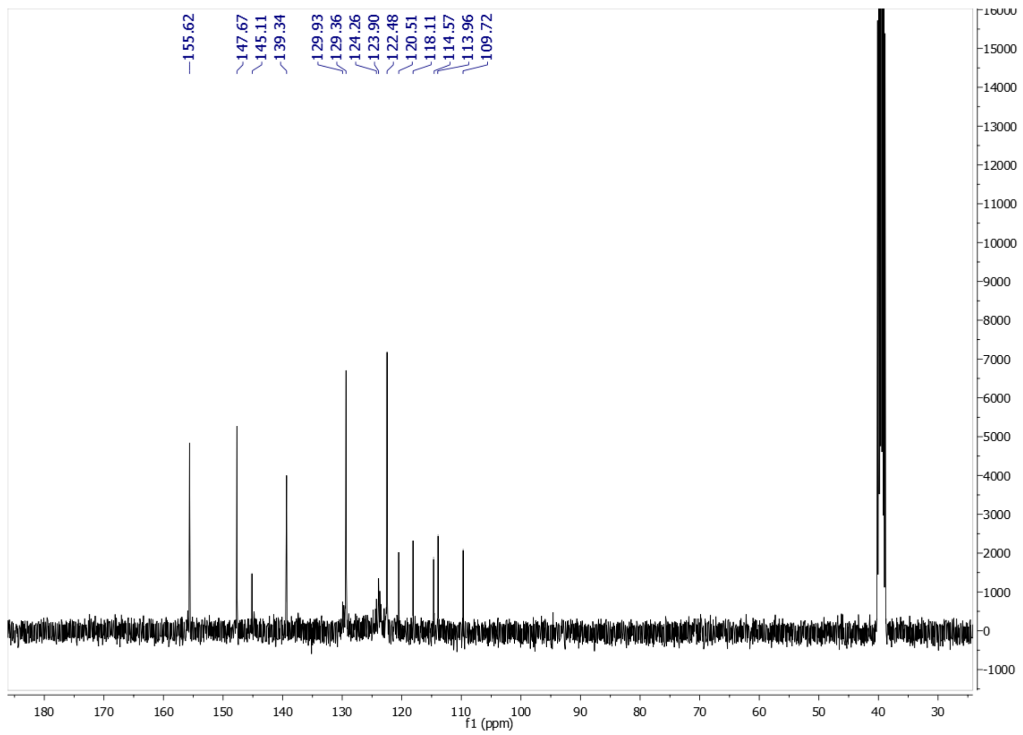

Figure S80. ^{13}C NMR of compound **42** at 101 MHz (DMSO-d_6)
